# Temporal Dynamics and Intermediate Product Formation in DOM Phototransformation Revealed by Liquid Chromatography Ultrahigh-Resolution Mass Spectrometry

**DOI:** 10.1021/acs.est.5c01986

**Published:** 2025-06-28

**Authors:** Peter Herzsprung, Aleksandr Sobolev, Wolf von Tümpling, Norbert Kamjunke, Michael Schwidder, Oliver J. Lechtenfeld

**Affiliations:** † UFZ − Helmholtz Centre for Environmental Research, Department Lake Research, Brückstraße 3a, Magdeburg D-39114, Germany; § UFZ − Helmholtz Centre for Environmental Research, Department River Ecology, Brückstraße 3a, Magdeburg D-39114, Germany; ‡ 9376 Otto-von-Guericke University Magdeburg, Universitätsplatz 2, Magdeburg D-39106, Germany; ∥ UFZ − Helmholtz Centre for Environmental Research, Department Analytical Chemistry, Research Group BioGeoOmics, Permoserstr. 15, Leipzig D-04318, Germany

**Keywords:** LC-FT-ICR-MS, intermediate products, dissolved organic matter, biogeochemical cycling, high time resolution, photo degradation, WWTP effluent

## Abstract

The complex composition of dissolved organic matter (DOM) has been extensively studied by modern high-resolution analytical methods. However, DOM reactivity is still enigmatic due to a lack of experimental data with sufficiently high temporal resolution to resolve the intrinsic dynamics within DOM. Likewise, extensive isomeric overlap prevents studying transformation of DOM components with respect to their chemical properties, e.g., molecular polarity. Online ultrahigh-performance liquid chromatography with ultrahigh-resolution mass spectrometry (UHPLC-UHRMS) increases the resolution of isomeric DOM composition across a wide range of polarity. We performed a TiO_2_-aided photo-irradiation experiment with wastewater treatment plant effluent with high temporal sampling resolution (8 time points, 5 h irradiation). Besides new products (<10%) and removed components (25–60%), intermediate products (*IntP*) were also found, representing 20–60% of components within distinct polarity fractions. The reaction time to reach the peak magnitude maximum was positively related to the H/C ratio of *IntP*. About 35% of the DOM components showed different reactivities for different polarity fractions. If applied to experiments in the future, our approach offers new perspectives for biogeochemical interpretation and provides important information for drinking water processing or wastewater treatment with respect to potential toxic *IntP*.

## Graphical abstract

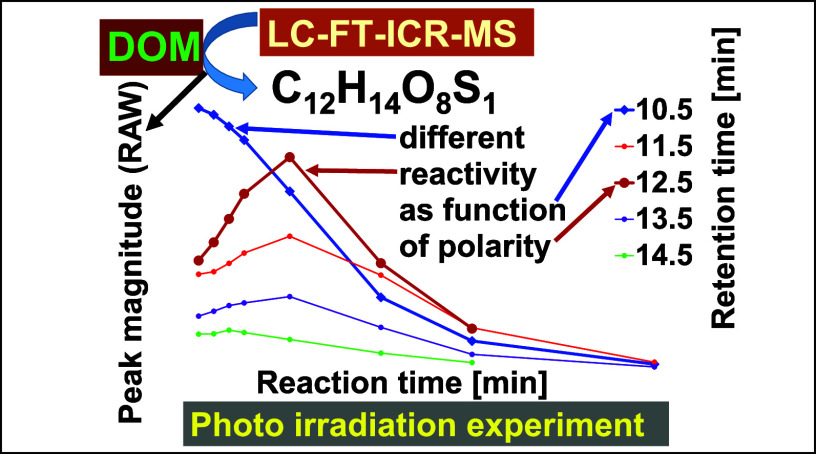

## 1 Introduction

Dissolved organic matter (DOM) is a highly complex mixture of degraded biomolecules containing a large diversity of molecular structures and isomers—the majority of compounds being still unresolved.^1,2^ DOM molecular composition has been extensively explored over the last decades with ultrahigh-resolution mass spectrometry (UHRMS like Orbitrap or Fourier transform ion cyclotron resonance, FT-ICR) using direct infusion (DI).^3–7^ However, shedding light on the isomeric composition of individual molecular formulas (MF), which will be crucial for explaining DOM reactivity, is still a great challenge. Combining NMR techniques with FT-ICR-MS^6,8,9^ or FTIR^10^ can provide insights into the structural features of DOM on a bulk level for biogeochemical classes. Trapped ion mobility separation and tandem MS provides structural information on DOM molecules but is far from providing comprehensive structural resolution for thousands of MF.^11,12^ Coupling ultrahigh performance liquid chromatography (UHPLC)—especially reversed phase—with UHRMS increases chemical resolution and provides more detailed information on DOM, enabling the separation of more polar/hydrophilic and less polar/hydrophobic fractions of individual *m*/*z* ratios, offering promising pathways for structural identification.^13–15^

Next to biogeochemically relevant compositional variation of DOM in different ecosystems, a major benefit of UHRMS is that it allows comprehensive, nontargeted investigations of DOM reactivity, e.g., along surface water transients or in engineered systems like wastewater treatment plants.^16–18^ Here, reactivity can relate to physicochemical processes like adsorption/desorption, photochemical transformations, or microbial production and degradation. In this context, DI-FT-ICR-MS was applied to describe DOM turnover in waters.^19–23^ Microbial DOM transformations were extensively studied in rivers and reservoirs.^20,23,24^

A typical approach to FT-ICR MS data evaluation (applicable to DI and LC data sets) is the calculation of average molecular descriptors (H/C, O/C, mass, aromaticity index, and others) in order to address DOM quality changes in response to a chemical or microbial process.^17,25^ Often, the absence of an MF after reaction was interpreted as degradation, while the new occurrence of the MF was interpreted as production.^20,25–29^ The drawback of this presence/absence (P/A) evaluation approach is that unique MFs often have low abundances near the signal/noise threshold,^30,31^ raising questions about their statistical robustness and quantitative importance.

More sophisticated approaches also evaluate intensity differences of the common MF at the start and end of the experiment, as well as considering intermediate reaction time points.^17,22,24,32^ This can help to identify MF with high or low reactivity and hence to extract key mass features, i.e., the most reactive MF within a given experiment (e.g., differential photo- and microbially reactive MF).^24^

The main disadvantage in comparing MF peak magnitudes using DI-FT-ICR-MS is the assumption that each MF has the same isomeric composition in all samples.^24,33^ LC-FT-ICR-MS enables chromatographic separation of DOM into more and less polar components of the same MF. Early studies showed that MFs in different samples do not have identical isomeric compositions, but the hydrophilic and hydrophobic parts were different and also showed different reactivities.^13,14^

Here, we present a strategy to overcome the disadvantages of using DI-FT-ICR-MS and the P/A evaluation of MF reactivities. Although no conclusive information on the exact isomeric structures can be obtained, LC-FT-ICR-MS provides information about the polarity of MF and, by high time resolution, the assignment of reactivity classes (production, degradation, resistance, etc.) can be improved.

To demonstrate the feasibility of this concept, we conducted a photo-irradiation experiment simulating natural sunlight with organic matter from a wastewater treatment plant (WWTP), discharging into the Holtemme River in Germany. Although we are focusing here on data evaluation strategies in the context of biogeochemical processing, wastewater treatment is increasingly gaining interest in addressing both emerging contaminants and organic matter.^17,28,34^

High time resolution enables—besides reliable assignment of product formation and degradation—direct monitoring of transformation dynamics. We propose an extension of the commonly used DOM reactivity classification—comprising “production,” “degradation,” and “resistant” compounds—by introducing a new category: “intermediate products (*IntP*).”

The objectives of this study are 3-fold. First, we aim to evaluate the variability of reactivity classes in relation to molecule polarity and molecular formula (MF) classes (CHO, CHNO, CHOS, and CHNOS). A particular focus is placed on quantifying the fractions of *IntP* compared to produced, degraded, and resistant compounds (Section 3.2). Second, we examine the reactivity of molecular formulas as a function of key chemical descriptors, including H/C and O/C ratios and molecular mass (Section 3.3). Third, we assess whether compounds with identical MF can be assigned to different reactivity classes depending on polarity, as determined via chromatographic separation coupled to FT-ICR MS (Section 3.4). In combination with high time resolution, this approach reveals that the widely adopted “start-and-end” model—based on compound P/A or peak magnitude differences—oversimplifies transformation dynamics and can lead to incorrect reactivity class assignments.

## 2 Materials and Methods

### 2.1 Photo Degradation Experiment

An effluent sample of 6 L was collected from the WWTP Silstedt before entering the Holtemme River in Germany on March 10, 2022 (Figure S1). The influence of this WWTP on DOM river quality had been previously studied.^23^ The sample was kept at 4 °C after transport to the laboratory until the start of the photo-irradiation experiment 4 days later. The water was filtered through precombusted (450 °C, 4 h) glass microfiber filters (47 mm diameter, Whatman GF/F, Cytiva, China) and distributed to two quartz glass bottles of 1 L each (denoted as samples “A” and “B” thereafter). Samples were irradiated for 5 h with a UVACUBE system (Figure S2, Hönle, Gilching, Germany), providing a dose of 28 mW/cm^2^ at the bottle bottom. Further experimental details are provided in SI1. As a limitation, DOM reactivity cannot be exclusively attributed to photochemical transformation since the temperature increased during the experiment (Figure S3).

### 2.2 LC-FT-ICR-MS Measurements and Data Processing

In total, 8 experimental time points (TPs) were measured for each of the two replicate samples, A and B: before the start of the experiment (*t*_0_) and after 10 (*t*_1_), 20 (*t*_2_), 30 (*t*_3_), 60 (*t*_4_), 120 (*t*_5_), 180 (*t*_6_), and 300 (*t*_7_) min.

Filtered samples (0.2 μm, Minisart RC4, Sartorius, Germany) were measured at their native concentrations without solid-phase extraction. A reversed-phase ultrahigh-performance liquid chromatography (UHPLC) method employing a postcolumn countergradient was used. LC-MS chromatograms were segmented into 13 one-minute segments between 5.5 and 20.5 min representing the elution according to molecule polarity. Mass spectra within each segment were averaged and MF assigned with the Lambda-Miner.^35^ Raw mass peak magnitudes (denoted as RAW) were directly used without further intensity normalization, as this has been shown to produce robust and reliable results for LC-FT-ICR-MS results of DOM.^36,37^ Further details of the chromatography and FT-ICR-MS methods, as well as raw data processing including molecular formula (MF) assignment, are described in SI2 and in the literature.^13–15^

### 2.3 Definition of Reactivity Classes

The reactivity classes are defined in Table 1 based on increased or decreased RAW values and their specific RAW differences. Reactivity classes were assigned for each MF and *RT_i_*. The relative peak magnitude difference (δRAW_i_) is calculated according to eq 1:

1 $$\delta RAW_{i}=\frac{RAW_{i}^{End}-RAW_{i}^{Start}}{RAW_{i}^{Start}}$$

**Table 1:** Definition of Reactivity Classes Used in This Manuscript^a^ ^b^ ^c^

Reactivity class^d^	Name	Short description	Definition	SI Figure
*Prod*	Product	δRAW positive (δRAW > 0.265)	STEP 3c,d	S4A,B
*Degr*	Degraded compound	δRAW negative (δRAW < −0.265)	STEP 3c,d	S4E,F
*IntP*	Intermediate product	increasing and later decreasing RAW,^f^ MF present at all eight TPs	STEP 3b	S5A,B
*<IntP>*	like *IntP*	MF not detected both at the start (*t*_0_) and at the end (*t*_7_) of experiment, maximum S/N*(p)* > 3 × S/N(4) threshold	STEP 3b	S5C,D
*IntP>*	like *IntP*	increasing^f^ from the start (*t*_0_); not detected at the end (*t*_7_) of experiment	STEP 3b	S5G,H
*<IntP*	like *IntP*	decreasing^f^ toward the end (*t*_7_); not detected at the start (*t*_0_) of experiment	STEP 3b	S5G,F
*<Prod*	like *Prod*	not detected at the start (*t*_0_), –0.265 < δRAW <^f^ 0.265; S/N*(p)* > 3 × S/N threshold^e^	STEP 3c,d	S4C,D
*Degr>*	like *Degr*	not detected at the end (*t*_7_), δRAW not negative,^f^ maximum S/N*(p)* > 3 × S/N threshold^e^	STEP 3c,d	S4G,H
*Res*	Resistant	no significant δRAW^f^ were found; present in all samples and/or all S/N*(p)* < 3 × S/N(4)	STEP 3c,d	S6A,B
*r.n.a.*	Reactivity not assigned	at least one data gap, or detected at only one of eight TPs	STEP 3a	S6 C,D,E,F
*n.d.*	Not detected	all S/N*(p)* < S/N(4) in all 8 samples at one RT (at all TPs)		-

a The relative change in raw mass peak magnitudes (δRAW) and peak signal-to-noise ratio (S/N­(*p*)) was used to distinguish reactivity classes.

b S/N­(4): Signal to noise ratio threshold = 4.

c S/N*(p)*: Peak signal-to-noise ratio.

d Abbreviation as used in main text and figures.

e The maximum S/N*(p)* must be larger than three times the peak picking threshold (here: S/N = 4, named as S/N(4)).

f Assuming that |δRAW| < 0.265 is not significant as this was the calculated threshold for accepting or excluding MF basing on experimental replicates (95^th^ percentile, see STEP 1, Section 2.4.1).

RAW_*i*_^Start^ is the peak magnitude value at the earlier time point; RAW_*i*_^End^ is the peak magnitude value at the later time point .

Examples of reactivity classes, showing time courses by plotting RAW versus time, are provided in SI5 (Figures S4–S6). Similar approaches, but without the definition of *IntP*, have already been reported.^24,38^ The calculation of the δRAW threshold (0.265) used in Table 1 is addressed in Section 2.4.1, STEP 1 and explained in detail in SI3.

### 2.4 Data Evaluation Procedure

A strategy was developed for the analysis and evaluation of the time-series data from the photoirradiation experiment (Figure 1).

**Figure 1:**
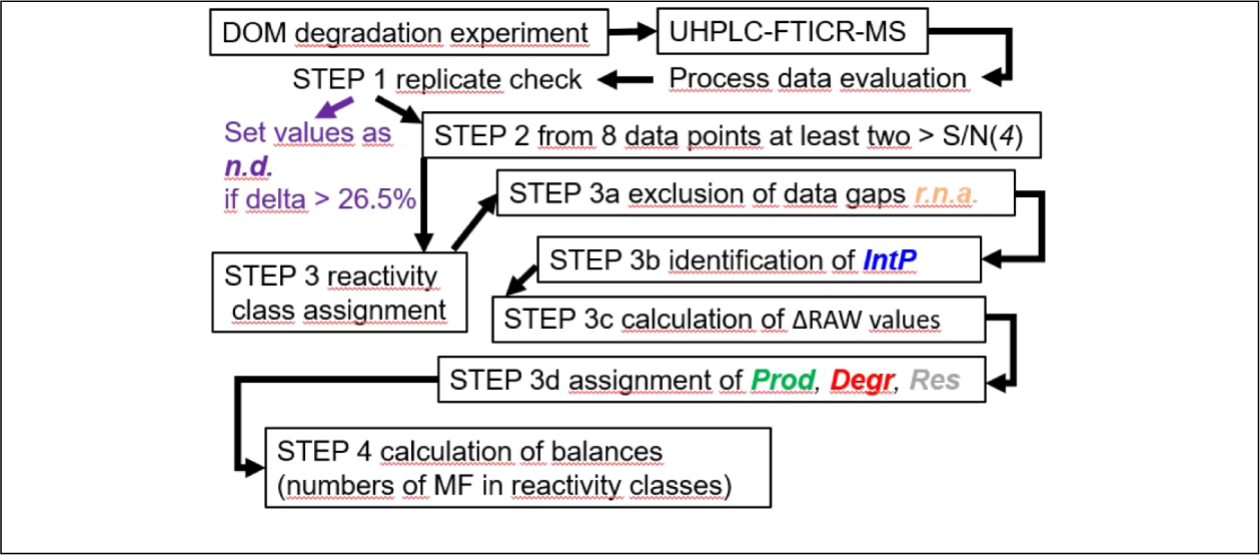
Flowchart for the experiment, mass spectrometry measurement and data evaluation steps.

#### 2.4.1 Check for Reproducibility

**STEP 1**: replicate check (SI3): The RAW peak magnitude reproducibility was evaluated for the two replicates (A and B) at each experimental TP. The absolute differences of the peak magnitudes δRAW_abs_ (δRAW_abs_ = |RAW_A_ – RAW_B_|) were calculated for each MF and each chromatographic segment (details in SI3). An average RAW value (RAW_A_ + RAW_B_)/2) was then calculated for each MF if their relative difference δRAW_rel_ = |RAW_A_ – RAW_B_|/[(RAW_A_ + RAW_B_)/2] was smaller than the 95th percentile of all δRAW_rel_. Otherwise, the RAW value for this MF was set to not determined (*n.d.*) (at the corresponding RT and TP) and excluded from further analysis. A full balance of valid and excluded MF is shown in Table S5. The 95th percentile was 0.265 (26.5%). In the following (STEP 3b,c), all δRAW_rel_ were regarded as significant (for different TPs) if larger than this 26.5% threshold. This peak magnitude difference threshold is comparable to Phungsai et al. (30%).^39^

#### 2.4.2 Evaluation of Reactivity Classes

**STEP 2**: check for minimum number of data points (SI4): For each DOM MF, 104 RAW values could potentially be detected (13 segments for each of the eight TPs; SI2.3, Table S3). Here, only MFs were considered where at least two RAW values (i.e., two TPs) were detected with S/N*(p)* > S/N(4) for at least one of the 13 RTs. S/N*(p)* is the peak signal-to-noise ratio with values provided in es5c01986_si_003.xlsx, Sheet S2, and S/N(4) is the peak picking threshold (=4).

**STEP 3:** reactivity class assignment: This is a sequence of specific search and calculation routines from data sheets in order to assign the defined reactivity classes in Table 1 to each MF at each RT.

STEP 3a: exclusion of data gaps (*r.n.a.*) (SI4.1)

A data gap is given if S/N*(p)* < S/N(4) between two S/N*(p)* values > S/N(4), for example, S/N*(p)*(*t*_0_) > S/N(4), S/N*(p)*(*t*_1_) < S*/*N­(4), S/N*(p)*(*t*_2_) > S/N(4). The complete search is explained in SI4.1. MFs having data gaps (or having only one S/N*(p)* > S/N(4) of eight S/N*(p)*(*t*)) are denoted as *r.n.a.* at the corresponding RT.

STEP 3b: identification of intermediate products (*IntP*, <*IntP*>, <*IntP*, *IntP*>) (SI4.2)

The reactivity class *IntP* is assigned to an MF whose RAW is significantly increased after the start of the experiment and afterward significantly decreased. All variations for intermediate products (*IntP*, <*IntP*>, <*IntP*, *IntP*>) are searched only for MF where no data gap was found (already assigned in STEP 3a). In principle, the intermediate data points RAW (*t*_1_ *– t*_6_) are checked versus the start RAW­(*t*_0_) and end RAW­(*t*_7_) if their maximum peak magnitude is significantly larger compared to that at the start and end. An example of *IntP* is provided in Figure 2.

**Figure 2:**
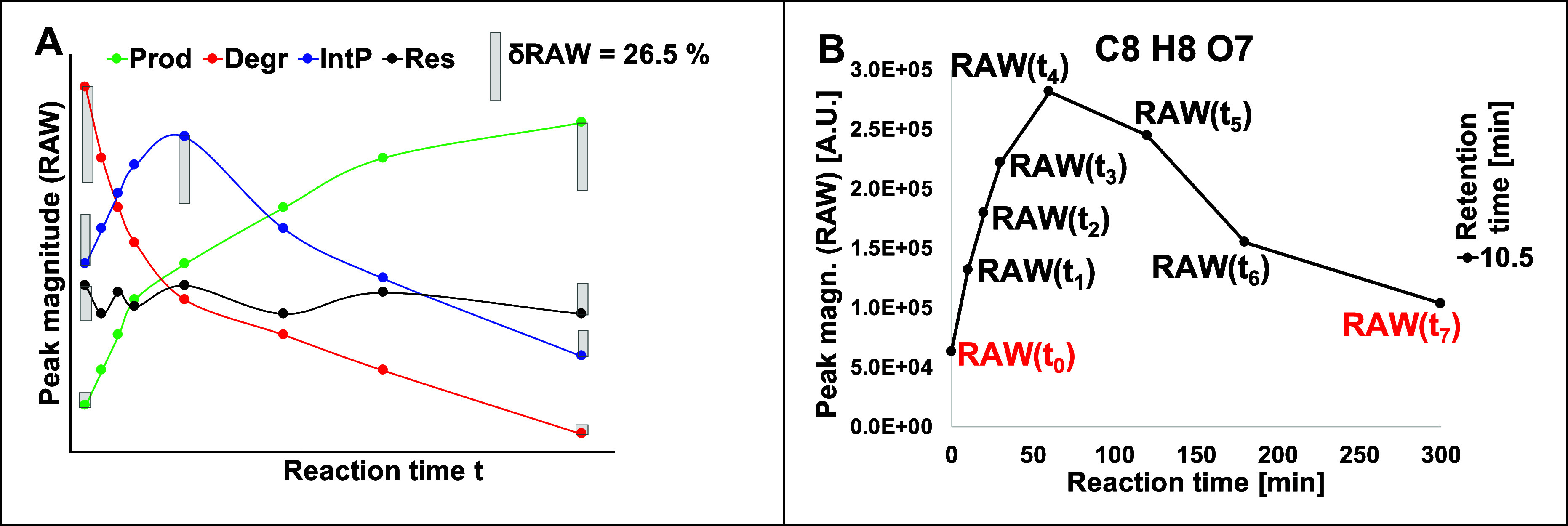
(A) Examples for reaction time courses for a molecular formula with reactivity classes produced (Prod, green), degraded (Degr, red), intermediate product (IntP, blue), and resistant (Res, black). δRAW = 0.265 = 26.5% is shown as the minimum threshold for significance. (B) Example for an IntP (with maximum RAW peak magnitude at t_4_) and designation of peak magnitudes at reaction time points.

STEP 3c: calculation of δRAW values (SI4.3)

The first and last valid RAW values (valid if S/N*(p)* > S/N(4)) are searched in the direction from *t*_0_ to *t*_7_. The search is applied to the complete data set independently if *r.n.a.* or *n.d.* or *IntP* had been already assigned to an MF. The δRAW values (eq 1) can be positive (suggesting product) or negative (degraded). This step is a preparation for the assignment of the reactivity classes *Prod*, *Degr*, and *Res*, which were not yet assigned in the earlier steps.

STEP 3d: assignment of *Prod*, *Degr*, and *Res* (SI4.4)

All MFs which are no *r.n.a* and no *IntP*, <*IntP*>, *IntP*>, <*IntP* are evaluated considering their δRAW values to be products (*Prod* or <*Prod*), degraded (*Degr* or *Degr*>) or resistant (*Res*). *Prod* has significantly positive δRAW. <*Prod* are defined if the maximum S/N*(p)* was three times larger than S/N(4) and if S/N*(p)* start (*t*_0_) < S/N(4), but δRAW was not significant as for *Prod*. If the δRAW is significantly negative, then it is assigned to be *Degr*. *Degr*> is assigned if the maximum S/N*(p)* was three times larger than S/N(4) and if S/N*(p)* end (*t*_7_) < S/N(4). *Res* is assigned to MF with no significant δRAW (all 8 time points with RAW > S/N(4)) or if the maximum S/N*(p)* was not three times larger than S/N(4).

STEP 4: calculation of balances

For each RT, the MF with assigned reactivities were counted, and fractions of reactivity classes were calculated. The sum of MF in the classes *Prod*, <*Prod*, *Degr*, *Degr*>, *IntP*, <*IntP*>, <*IntP*, *IntP*>, *Res*, and *r.n.a.* was set as 100%. The class *n.d.* was ignored in this relative balancing because, without any detected signal, no reactivity can be determined. The results were listed in Sheet SI and displayed in Figure S7A. The results for RT = 20.5 min were displayed but not discussed in detail due to the limited number of assigned MF.

For simplification of understanding the relations of reactivities, the classes *Prod* and <*Prod* were combined to *Prod*, *Degr,* and *Degr*> to *Degr*, *IntP* and <*IntP*> and <*IntP* and *IntP*> to *IntP* respectively (es5c01986_si_002.xlsx, Sheet SI x.1). In the simplified balance the sum of *Prod*, *Degr*, *IntP*, *Res* was set to 100% (all MF with assigned reactivity) (Figures 3 and S7C,D). The data were used for the LC model, which will be later compared to models without polarity resolution (Section 2.4.2) and without time resolution (Section 2.4.3).

**Figure 3:**
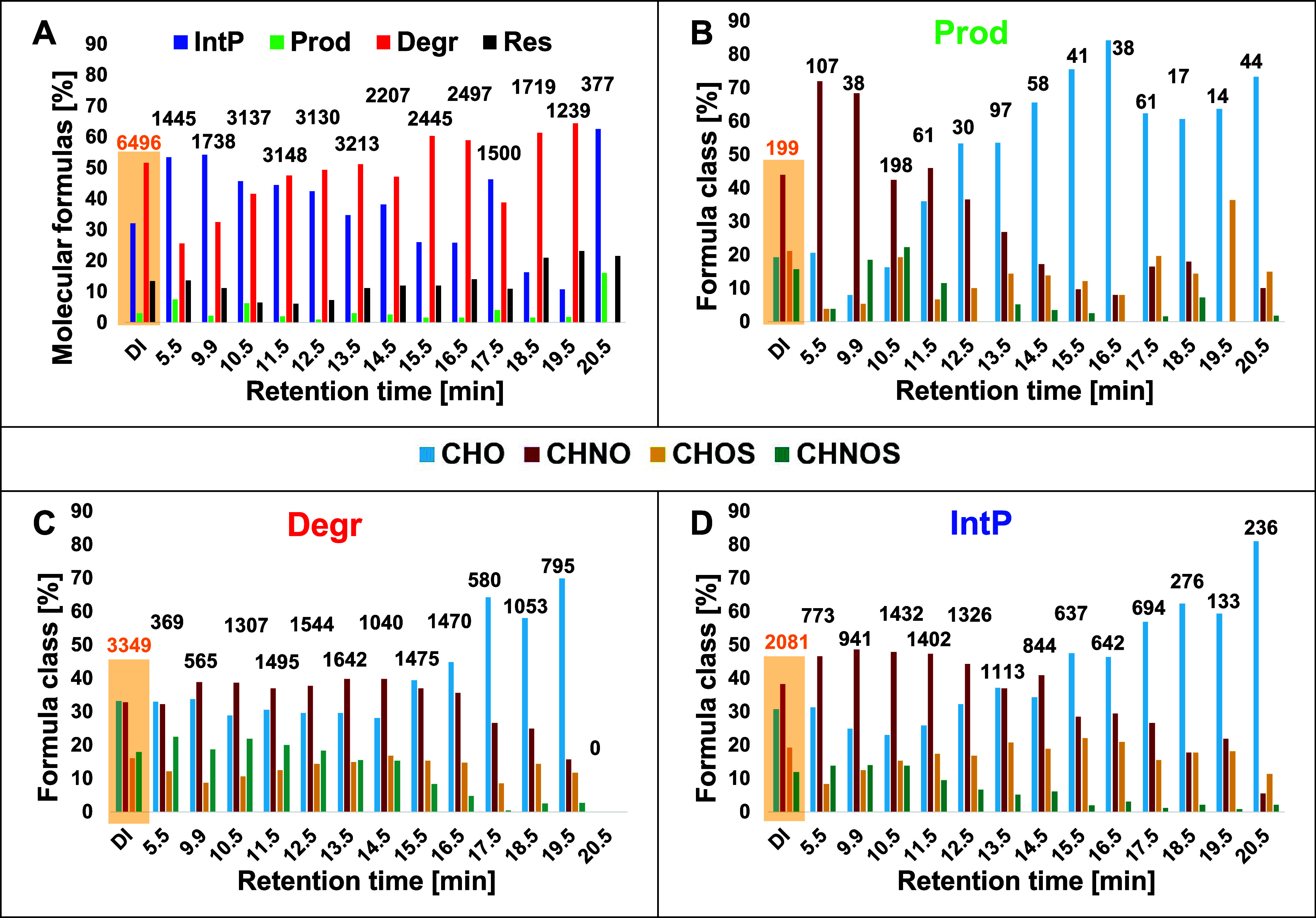
(A) Distribution of reactivity classes (as fraction of all molecular formula, MF) as a function of retention time (RT). Reactivity classes were combined as described in STEP 4. (B–D) Distribution of MF classes (CHO, CHNO, CHOS, CHNOS) of assigned products (B: Prod), degraded MF (C: Degr) and intermediate products (D: IntP). Note that segment at 20.5 min was excluded from further discussion due to the high number of MF classified as r.n.a. The corresponding data for simulated *pseudo*-DI-FT-ICR-MS data are shown in the orange box, as described in Section 2.4.2.

#### 2.4.3 Simulation of a DI Data Set

To test the effect of chromatographic separation (i.e., based on molecular polarity) on the assignment of reactivity classes for each MF, a *pseudo*-DI data set was simulated from the LC-FT-ICR-MS data. Considering MFs that were detected at least two times across all RT segments, an average RAW value was calculated. This resulted in a data set that reflects peak abundances of a DI mass spectrum but with higher MF coverage in the chemical space.^13^ These data are used for the DI 8TP model (i.e., with time resolution, without polarity resolution).

#### 2.4.4 Simulation of a Presence/Absence (P/A) and 2TP RAW Data Set

A test of the effect of higher experimental TP resolution with a simple start-to-end difference on the assignment of reactivity classes was done with the start S/N*(p)*(*t*_0_) and end S/N*(p)*(*t*_7_) values from the *pseudo*-DI data set. The MF is assigned as *Prod*, if S/N*(p)*(*t*_0_) < S/N(4) and S/N*(p)*(*t*_7_) > S/N(4), as *Degr*, if S/N*(p)*(*t*_0_) > S/N(4) and S/N*(p)*(*t*_7_) < S/N(4), and as *Res* if S/N*(p)*(*t*_0_) > S/N(4) and S/N*(p)*(*t*_7_) > S/N(4). This approach is here termed the presence–absence (P/A) model. Further considering the RAW values (as described in STEP 3c), the P/A model extends to a two-point peak magnitude (2TP RAW) model using eq 1 for δRAW calculation. Both models, P/A and 2TP RAW, do not provide time and polarity resolution. All four models P/A, 2TP RAW, DI 8TP, and LC are used and discussed in Section 3.4

## 3 Results and Discussion

### 3.1 DOC Concentration

The DOC concentration decreased from the start (*t*_0_: 7.4 mg/L) to the end of the experiment (*t*_7_: 5.3 mg/L; Table S1).

### 3.2 Polarity Resolved DOM Photoreactivity

A total of 9409 distinct MFs (of those 3000 CHO, 3318 CHNO, 1533 CHOS, 1530 CHNOS) were considered for the reactivity class evaluation after replicate averaging. The distribution of MF in the chemical space (molecular H/C, O/C, and mass) aligned with previous results from RPLC-FT-ICR-MS of wastewater effluent samples, showing high O/C values for high polarity and low O/C values for low polarity segments (Figures S13–25).^36^ In order to understand the reactivity of DOM on a polarity-based isomeric level, reactivity classes were aggregated for each RT (Figures 3 and S7). More than 50% (average: 64%) of MF could be assigned to a reactivity class at each RT (except for 20.5 min), supporting high coverage of DOM transformation within the sample (Sheet SI).

The fractions of each simplified reactivity class (the combination of reactivity classes is explained in STEP4) ranged from about 1% (RT = 12.5 min) to 7.5% (RT = 5.5 min) for *Prod*, from 26% (RT = 5.5 min) to 64% (RT = 19.5 min) for *Degr*, from 11% (RT = 19.5 min) to 54% (RT = 9.9 min) for *IntP*, and from 6% (RT = 11.5 min) to 23% (RT = 19.5 min) for *Res*. The *Degr* and *IntP* classes dominate over *Res* and *Prod* in line with the decrease in the DOC concentration. Notably, *IntP* represents a considerable fraction (up to 50%) of all MF which has achieved little attention so far. In contrast to *IntP* (characterized by first increasing and then decreasing RAW), a negligible number of MF was found with first decreasing and then increasing RAW values (cf. Section SI9).

Another large fraction of MF could not be assigned to a reactivity class (group *r.n.a.*: 26%–47% for RT = 5.5–19.5 min; 63% for RT = 20.5 min; Figure S7A) due to missing data points (valid RAW with S/N*(p)* > S/N(4)) *along* the reaction TP (STEP 3a). MFs of the *r.n.a.* class were often represented by low RAW values near the S/N(4) threshold (e.g., C_12_H_13_N_1_O_11_ in Figure S6F) or showed low reproducibility concerning the two replicates A and B (and were hence excluded by the reproducibility filter and set to <S/N(4), STEP1, i.e., *n.d.*). An example of this effect on data gaps is shown in Figure S6D and Screenshot 13. Such data gaps are a consequence of the stringent replicate intensity filter and high temporal resolution of the photoirradiation experiment (increasing likelihood of replicate intensity mismatches or below detection with increasing number of experimental time points). However, experimental replication and high temporal resolution increase the robustness of assigned reactivity classes and limit spurious and potentially wrong reactivity classification. As a limitation *r.n.a* might be overestimated and other reactivity classes underestimated.

The segment at 20.5 min (i.e., DOM components with the lowest polarity) had the largest fraction of *r.n.a* (63.3%, Figure S7A), and no MF in this segment was detected at all experimental TPs (e.g., with presence count 8). This indicates overall low detectability or reproducibility for the most hydrophobic DOM and justifies the exclusion of this segment from the discussion of DOM processing.

The fraction of the *IntP* class increased with increasing polarity (lower RT, except 17.5 min; Figure 3A). In contrast, the fraction of the *Degr* class was approximately 50% for the less polar segments (13.5–19.5 min) and showed a decreasing trend (except at 14.5 and 17.5 min) with increasing polarity. Similarly, MFs of the *Res* class were least abundant at RT = 10.5 −12.5 min and showed a maximum in the less polar segments (RT = 18.5, 19.5 min; 21–23% of MF). This indicates that less polar, more hydrophobic components are preferentially degraded possibly leading to the production of more polar hydrophilic molecules.

Evidently, many polar components (RT = 5.5–11.5 min: 44–53%) first increased and later decreased during the experiment, resulting in their assignment as *IntP*. These *IntP*s have different (and changing) apparent net reaction rates depending on the progression of the experiment. For instance, C_8_H_8_O_4_S_1_ monotonously increased from the start of the experiment (*t*_0_) until *t*_5_ and then decreased toward the end (*t*_7_, Figure 4C). This suggests that the precursor of C_8_H_8_O_4_S_1_ is diminished or fully consumed after *t*_5_ and that no further production of C_8_H_8_O_4_S_1_ can be observed. Likewise, with the increased abundance of C_8_H_8_O_4_S_1_ in the reaction mixture, subsequent degradation of this compound becomes more dominant, leading to a net decrease in the RAW magnitude.

**Figure 4:**
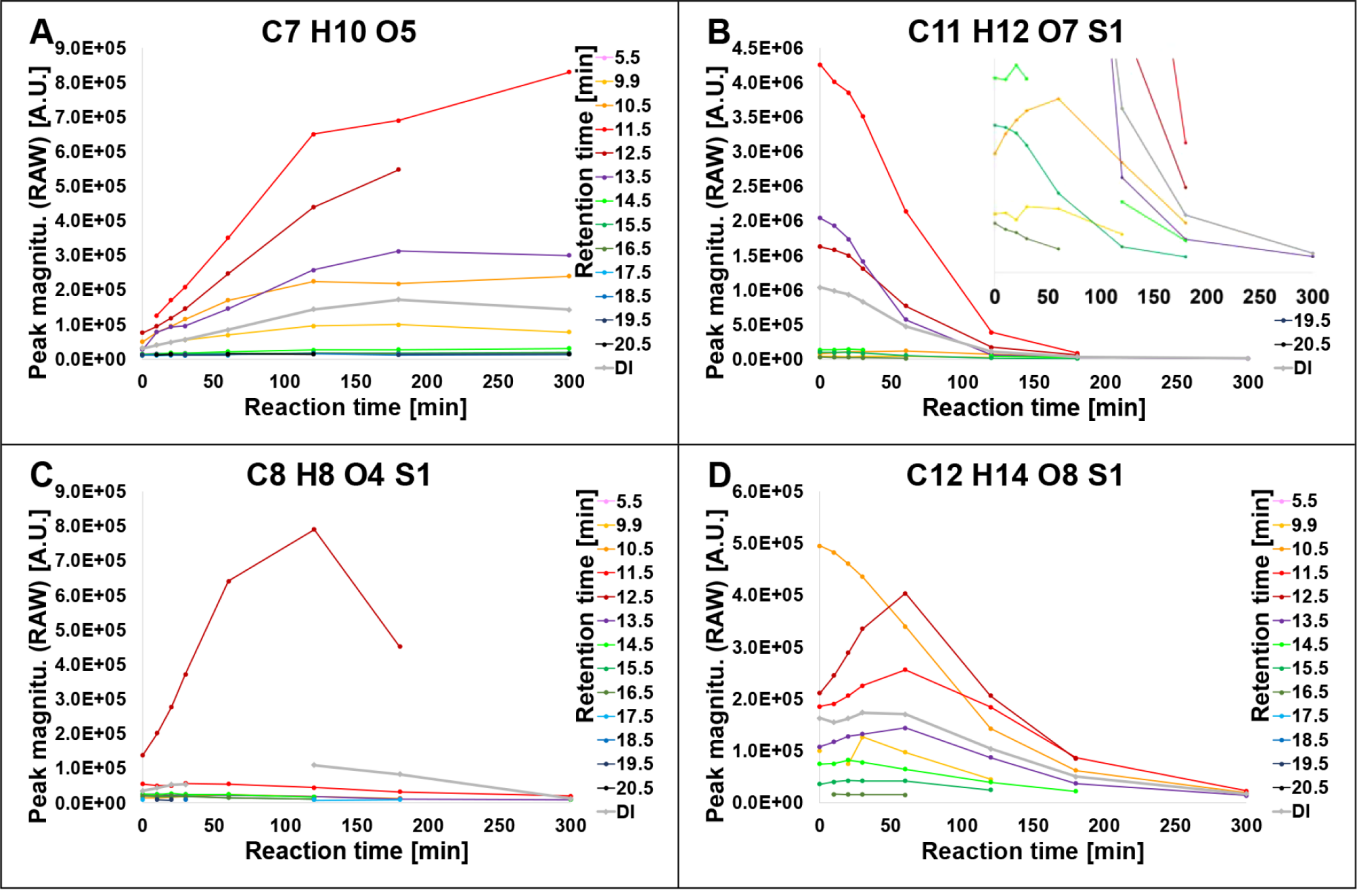
Reaction time courses of specific effluent DOM molecular formulas (MF) revealed by LC-FT-ICR-MS. (A) C_7_H_10_O_5_, classified as Prod (e.g., RT = 11.5 min), (B) C_11_H_12_O_7_S_1_, classified as Degr (e.g., RT = 11.5 min), (C) C_8_H_8_O_4_S_1_, classified as IntP (e.g., RT = 12.5 min), and (D) C_12_H_14_O_8_S_1_, with opposite reactivity at RT = 10.5 min (Degr) and at RT = 12.5 min (IntP).

In the studied effluent DOM sample, the polarity distribution of MF classes revealed an increase in the proportion of CHNO and CHNOS with polarity (CHNO from 27% at RT = 19.5 min to 44% at RT = 9.9 min, CHNOS from 3% at RT = 19.5 min to 19% at RT = 10.5 min), while that of CHO decreased (57% at RT = 19.5 min to 25% at RT = 10.5 min) and CHOS remained relatively unchanged (from 10% to 16%; Figure S7B). Importantly, the reactivity class distributions of each MF class (*Prod*., Figure 3B; *Degr*, Figure 3C; *IntP*, Figure 3D) clearly deviated from the overall distribution of MF classes (Figure S7B). The fraction of CHNO products strongly increased with polarity, reaching up to 70% of all *Prod* (Figure 3B). Such CHNO are mainly low molecular weight molecules (Figures S14–S16, H/C versus mass diagrams). The fraction of CHNO *IntP* (Figure 3D) was higher (5.5 min–12.5 min, hydrophilic) or lower (15.5 min–19.5 min, hydrophobic) compared to the initial MF class distribution (Figure S7B), while *Res* CHNO increased with RT (12.5 min–19.5 min, Figure S7C). The ratio between degraded CHNO and CHO was smaller than the overall ratio of CHNO to CHO components in the interval from 5.5 to 12.5 min (Figure 3C). Overall, we found a reversed reactivity pattern of the CHO class as compared to the CHNO class, suggesting that hydrophobic CHNO are more resistant to photodegradation than hydrophobic CHO (Figure S7C). At the same time, *Prod* and *IntP* were mainly characterized by polar CHNO components. Our results also show that CHO products are hydrophobic, while CHNO products are hydrophilic (Figure 3B).

Overall, the reactivity class assignment clearly differs depending on the polarity and the MF class (nonpolar MF mainly assigned as *Degr*, polar CHNO as *Prod* and *Intp*) during photoirradiation. Unexpectedly, the number of *IntP* is in the same order of magnitude compared to *Degr* but one order of magnitude higher compared to *Prod*. This is important for the overall reactivity assignment of DOM based on lab or mesoscale experiments with fixed duration, since the reactivity class may change depending on the progression of the experiment, which will depend on the specific configuration of the experiment (photochemical or microbial transformation, radiation source, DOM source, duration, and time resolution). As a limitation, the validity of the reactivity assignment is dependent on the used thresholds: the relative difference of replicate peak magnitudes (SI3), allowing or not allowing data gaps (SI4.1), exclusion of MF because of limited presence count (STEP 2), definition of *IntP* (SI 4.2), setting S/N limitation (SI 4.2), definition and calculation of δRAW (SI 4.3), and use of its threshold (SI 4.4) to search for *Prod* and *Degr*. The estimation of errors in counting the reactivity classes *Prod, Degr, IntP*, and *Res* is described on Page S24 and Figure S9.

### 3.3 Molecular Composition and Distribution of DOM Photoreactivity Classes

While polarity separation via LC proves useful for assigning a chemical property (here: polarity) to different photoreactivity classes among all DOM features, the molecular composition within each class also varies substantially. For the high polarity range, CHO and CHNO *IntP* showed mainly O/C > 0.5, and *Degr* CHO had higher O/C compared to *Degr* CHNO (RT = 10.5 min; Figure 5A,B). At the same time, *Degr* CHO were more unsaturated (i.e., lower H/C) whereas both saturated and unsaturated *Degr* CHNO were observed (Figure 5A,B). *Res* MF were mostly randomly distributed with respect to oxidation, saturation, and mass across segments (Figures 5A,B and S13–25). *Prod* of the CHNO and CHO classes showed low molecular masses (e.g., <300 Da for RT = 10.5 min, Figure S15). This is in accordance with observations that photoproducts are rather small molecules.^40^ Low molecular weight CHNO can contribute more to polar DOM fractions compared to CHO because the nitrogen atoms are potentially bound in amino- or amide groups. Hence, it is more probable for a CHNO precursor molecule to release a polar product compared to a polar CHO precursor, which can lose polarity by, for example, elimination of CO_2_.

**Figure 5:**
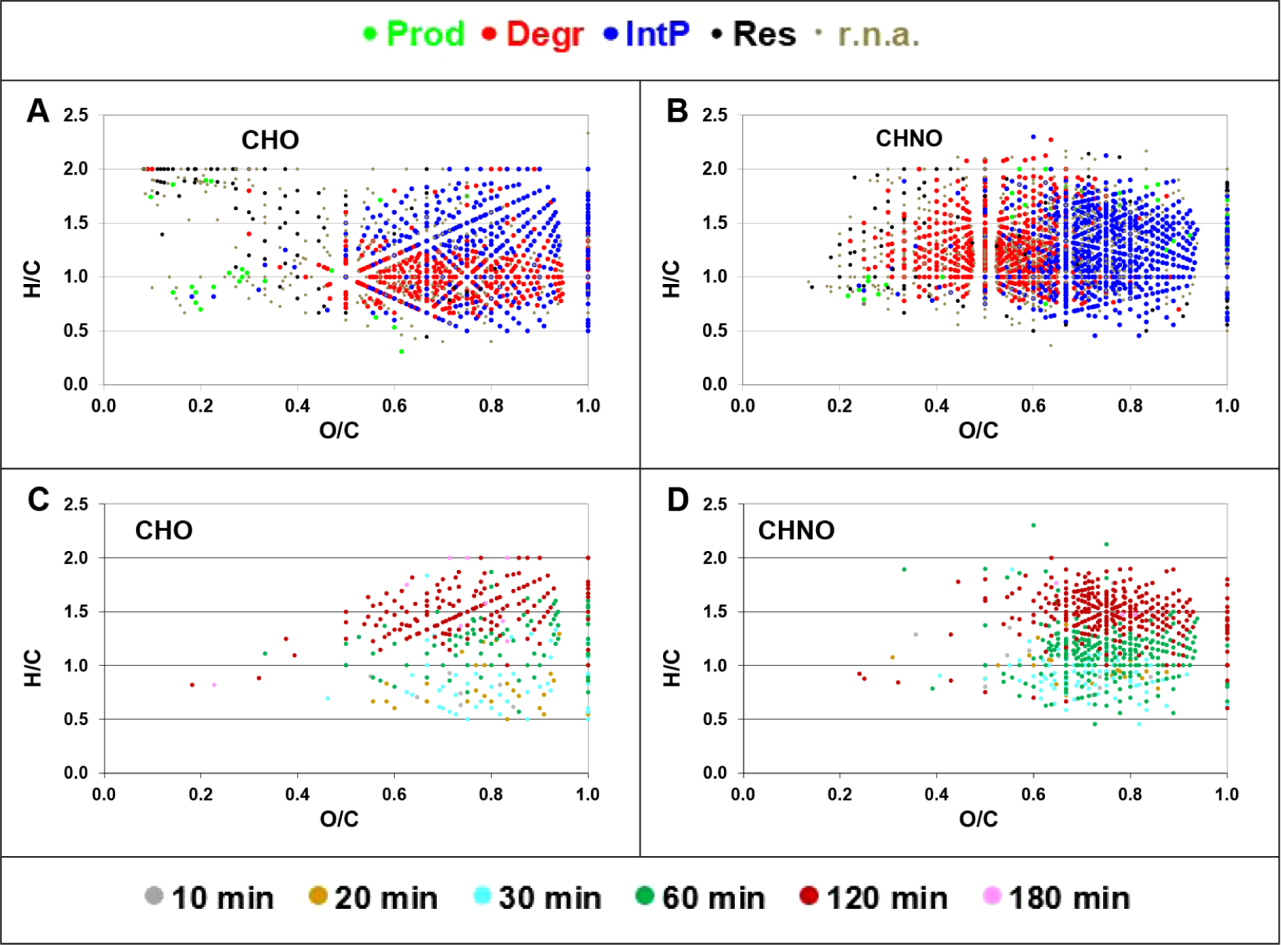
Distribution of assigned reactivity classes at RT = 10.5 min based on the molecular H/C vs O/C ratios. (A) CHO and (B) CHNO. Distribution of IntP as a function of the (reaction) time point where the maximum peak magnitude was observed for (C) CHO and (D) CHNO. The distributions of all reactivity classes of all RTs are shown in Section SI7.

However, *IntP* covered a wider mass range, involving larger molecules (Figure S15). This indicates that degradation (of the precursor molecules) does not always result in breaking of large molecules into several small molecules but instead transformations also result in small variations of the carbon skeleton, e.g., via reactions involving decarboxylation, desulfurization, deamination, oxidation of double bonds, and oxidation of aromatic rings without ring cleavage.

Due to the high temporal and chemical resolution, the time point of maximum magnitude for *IntP* could be estimated. As an example, all MF found as *IntP*s (*IntP* + <*IntP*> + *IntP*> + <*IntP*) were plotted in a van Krevelen diagram as a function of the reaction time of maximum RAW. Interestingly, CHO and CHNO MF with low H/C values (higher aromaticity) peaked early during the course of the reaction (e.g., at RT = 10.5 min; Figure 5C,D). This suggests that precursors of more aromatic components are more rapidly exhausted than precursors of more aliphatic MFs or that the products are also highly reactive. A more detailed analysis between the maximum time point and molecular descriptors is shown in SI8. Figure S26 shows examples of contrasting trends concerning the H/C. Particularly, the additional information on the *IntP* maximum abundance demonstrates the potential to better link molecular formula information with reactivity.

Overall, our results show that DOM photochemical reactivity is linked to both molecular composition and structure (defining their chemical properties and reactivity).

### 3.4 Novel Insights into Photochemical Reactivity of DOM

#### 3.4.1 Improved Reactivity Assignment by Higher Time Resolution

The example of C_11_H_12_O_7_S_1_ shows a clear degradation pathway along the experimental time course with S/N*(p)* < and S/N(4) at *t*_7_ (i.e., the compound was considered absent) for most segments (Figure 4B). However, at RT = 13.5 min, if only the P/A of C_11_H_12_O_7_S_1_ at the start and end of the experiment (two TP) was evaluated, this MF might have been classified as *Res*, because S/N­(*p*) at the end of the experiment is still > S/N(4) in this segment. Our high temporal resolution LC-FT-ICR-MS approach does identify it as degraded independently if the end S/N­(*p*) was > S/N(4) (RT = 13.5 min) or < S/N(4) (RT = 11.5 min, 12.5 min). The reaction time courses of C_8_H_8_O_4_S_1_ (Figure 4C), C_7_H_10_O_5_ (Figure 4A), and C_12_H_14_O_8_S_1_ (Figure 4D) further confirm the advantage of higher time resolution. If a two-time point experiment had been terminated at 60 min (*t*_4_), all three MF would have been identified as *Prod* (considering RAW differences by regarding RT = 12.5 min) or even as *Res*, if only their P/A had been considered. Termination of the experiment at 300 min (*t*_7_) or later would have identified these MF as *Degr* using a two TP approach. Although the high time resolution of our study also indicates S/N­(*p*) < S/N(4) at *t*_7_, the MF was further classified as *IntP*> at RT = 12.5 min. Note that the discussed effects are independent of the LC separation and are only related to the number of data points and the corresponding robustness/confidence in using peak magnitudes for the evaluation of MF reactivity.

Evaluation of the *pseudo-*DI data (using averaged RAW values from all segments) revealed 4456 MF (53% of a total of 8338) as *Degr* using the P/A model. Of those, the 2TP RAW model found only 3396 MF (41% of 8338) as *Degr*, but 1060 as *r.n.a.* because of start S/N­(*p*) < 3 × S/N(4). In the 2TP RAW model, we found in total 5447 (65% of 8338) MF to be degraded, of which the P/A model found 2051 (25% of 8338) as *Res* (Sheet SI). Of the 4456 MF identified as *Degr* in the P/A model 2621 (22% of 9394) were found again as *Degr* in the DI 8TP model, but 492 MF were found as *IntP*>, 479 as *Res* and 864 as *r.n.a.* (Sheet SI). Of the 5447 MF identified as *Degr* in the 2TP RAW model, 3700 (40% of 9394) were found as *Degr* in the DI 8TP model, but 799 MF were found as *IntP*, 194 as *Res,* and 754 as *r.n.a*.

These comparisons highlight that the choice of the experimental model (TPs/resolution) as well as decisions made during data processing (evaluation of peak magnitudes or only peak presence) greatly affect the proportions of the assigned reactivities. Potentially biased reactivity classes may be assigned by the oversimplified P/A model.

#### 3.4.2 Differentiation of MF Reactivity by Polarity Resolution

Chromatographic separation reveals more details about DOM component reactivity compared to the *pseudo*-DI data. The structural diversity of DOM is revealed by chromatographic separation, reflecting the distribution of an MF over several RT segments. It has been demonstrated that individual model compounds like vanillic acid show peak widths of less than 0.5 min.^15^ It cannot be completely excluded that compounds exist with lower chromatographic separation potential, and hence, the same isomer might be found in different segments. Likewise, an unknown number of isomers of an MF that were not chromatographically separated (e.g., due to very low abundances and very similar structures) may exist within each RT segment.

The DI 8TP model classified C_12_H_14_O_8_S_1_ (Figure 4D) as *Degr*. The polarity separation of isomers of this component uncovered different underlying reactivities. For instance, this MF decreased between start and 60 min (*t*_5_) at RT = 10.5 min, whereas the more hydrophobic fraction (RT = 12.5 min) strongly increased during this time interval. In agreement with the DI 8TP model, C_12_H_14_O_8_S_1_ was classified as *Degr* at RT = 10.5, 14.5, and 15.5 min. It was, however, classified as *IntP*/*IntP*> at RT = 11.5, 12.5, and 13.5 min, as *Res* at RT = 16.5 min and as *r.n.a.* at RT = 9.9 min. Using C_12_H_14_O_8_S_1_ as an example, known natural and synthetic compound entries in chemical databases (PubChem search 28.04.2024) already span four logP units (−2.5 to 1.5) and diverse structural units. It thus appears likely that a single MF spans different photochemical reactivities according to their RP-LC retention.

A substantial fraction of MF was assigned in both models to different reactivities (Figure 6). The 253 *Prod* MF in the pseudo-DI data were distributed to all reactivity classes using LC data (Figure 6A) with *Degr, IntP,* and *Res* showing a similar diversity of reactivity classes using LC (Figure 6B–D). These results clearly demonstrate that a single DOM component can exhibit different reactivities depending on the polarity of the unknown isomers contributing to the same molecular formula. Of note, while 1949 *r.n.a.* MF in the DI 8TP model could be assigned in part (on average to 190 MF from 5.5 to 19.5 min) to reactivity classes in the corresponding LC model (8 TPs, 13 segments), the overall fraction of *r.n.a.* MF was higher in the LC data due to more MF being close to the S/N(4) threshold.

**Figure 6:**
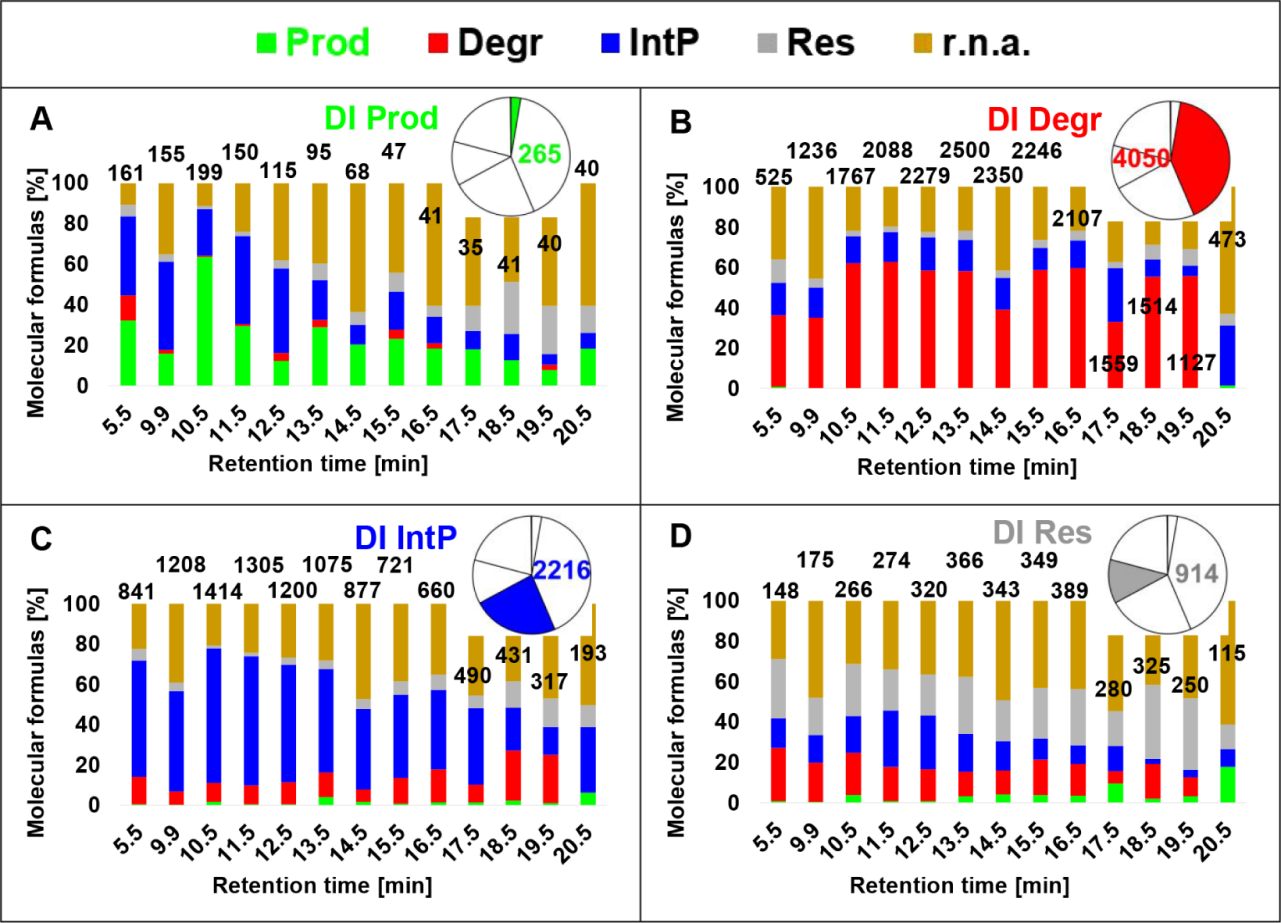
Comparison of reactivity class distribution, DI 8TP model vs LC model. (A) Distribution of reactivity classes in the LC model (Prod, green; Degr, red; IntP, blue; Res, gray; r.n.a., brown) for molecular formulas (MFs), which have been assigned as products in the DI 8TP model (“DI Prod), (B) distribution of degraded MF (”DI Degr”), (C) intermediate products (“DI IntP”), and (D) corresponding distribution of resistant MF (“DI Res”). Inset pie charts indicate the distribution of reactivity classes in the DI 8TP data set and values on bars indicate the number of MF of the respective DI 8TP reactivity class detected in each LC segment. Cf. data provided in es5c01986_si_002.xlsx, Sheet SI x.3.

Overall, about 30% (2781) of the 9409 distinct MF were assigned to two reactivity classes, e.g., in one segment as *Prod* and one as *Degr* or *Prod*/*IntP or Prod*/*Res* or *Degr*/*IntP* or *Degr*/*Res or IntP*/*Res* (Table 2). 5.3% (499) of all MF were assigned to three reactivity classes (for example, *Degr*, *IntP*, and *Res*), and 38 MF were assigned to all four classes (*Prod*, *Degr*, *IntP,* and *Res*, e.g., C_10_H_14_O_6_; Figure S28). These results demonstrate that a simple start and end experiment in combination with DI FT-ICR-MS does not provide sufficient resolution to robustly address the DOM reactivity. High time resolution in combination with UHPLC-FT-ICR-MS is thus required for a better description of biogeochemical DOM transformations.

**Table 2:** Balance of MF Assigned to Different^a^ Reactivity Classes Having Different Polarities (Different RT)

reactivity class assigned	*Prod*	*Degr*	*IntP*	*Res*
1 class	137	2062	1799	760
2 classes	28	28		
2 classes	106		106	
2 classes	32			32
2 classes		1792	1792	
2 classes		462		462
2 classes			361	361
3 classes	49	49	49	
3 classes	8	8		8
3 classes	62		62	62
3 classes		380	380	380
4 classes	38	38	38	38

a Note that *r.n.a.* has not been reconsidered in this balance as reactivity class. A balance including *r.n.a.* is shown in Table S7.

## 4 Implications

Data evaluation of DOM transformation experiments using LC-FT-ICR-MS is sophisticated with respect to the data amount and data structure complexity. The reaction dynamics of DOM components and their isomeric composition are still in their infancy. The recent LC-FT-ICR-MS developments now provide the opportunity to resolve different DOM polarity (or size) fractions. Our results demonstrate that the biogeochemical reactivity of DOM is very complex, and the current classification schema appears to be oversimplified with potential false conclusions about reactive DOM fractions. Monotonous RAW magnitude increases and decreases were observed for many MF, but much more complicated reaction time courses were also discovered, particularly intermediate products reflecting (potentially simultaneous) production and degradation processes. The relation of DOM reactivity to its chemistry was highlighted thoroughly by visualization of H/C versus O/C and molecular weight (Figures S10–S25) and discussed in detail in Section 3.3

Our approach using the simple classification of DOM reactivity might be the basis for standardized experiments for highly resolved classification of DOM reactivity from different sources. We tested one data set from the literature (shaded river water) using our data evaluation approach and found about 15% *IntP* compared to 30% *IntP* in the wastewater sample from the current study (SI 12 and Figure S31).^41^ The relationships between precursors and products by analysis of specific mass differences in a molecular network were not addressed in the present study. This can be achieved in the future using a temporal graph model to predict chemical transformations in complex DOM mixtures,^42^ coupled with structural information that may in future be adapted for LC-FT-ICR-MS to provide even more details (exchange between hydrophilic and hydrophobic fractions). Ultimately, this will help to better disentangle the DOM reactivity in complex environmental systems.

Addressing wastewater treatment, a closer investigation of reaction time courses is important, as the choice of reaction time can be critical. If the reaction time is too short, high concentrations of intermediate products may remain, some of which can be potentially toxic (e.g., as observed during chlorination disinfection).^43^ An effective process should therefore eliminate both the precursor molecules and most of the intermediate products. If toxicity data were available for DOM, this could help assess the degradation status and its relation to environmental toxicity.

## Supplementary Material







## Data Availability

Processed and quality checked data for all samples and segments are available from the UFZ Data Investigation Portal: 10.48758/ufz.15776. Raw MS files can be shared upon request.

## Notes

Detailed data evaluation description, additional visualization of reactivity distribution, and study site (PDF)
Data of reactivity distribution and reactivity evaluation model comparison (XLSX)
Molecular formula data with assigned reactivities and S/N­(*p*) data (XLSX)
