# Supplementary material for: Temporal Dynamics and Intermediate Product Formation in DOM Phototransformation Revealed by Liquid Chromatography Ultrahigh-Resolution Mass Spectrometry
Source: Environ Sci Technol. 2025 Jun 28;59(27):13787–97. doi: 10.1021/acs.est.5c01986 (PMC12269077; doi:10.1021/acs.est.5c01986)
Supplement: Supplementary file 1 [file es5c01986_si_001.pdf]

Supporting Information for

Temporal dynamics and intermediate product formation in DOM photo-transformation revealed by liquid chromatography ultrahigh-resolution mass spectrometry

Peter Herzsprung[*,†], *Aleksandr Sobolev*[&], *Wolf von Tümpling*[‡], *Norbert Kamjunke*[‡],

Michael Schwidder[&], *Oliver J. Lechtenfeld*[§]

AUTHOR ADDRESS

† UFZ – Helmholtz Centre for Environmental Research, Department Lake Research, Brückstraße 3a, D-39114 Magdeburg, Germany

‡ UFZ – Helmholtz Centre for Environmental Research, Department River Ecology, Brückstraße 3a, D-39114 Magdeburg, Germany

& Otto-von-Guericke University Magdeburg, Universitätsplatz 2, D-39106 Magdeburg

§ UFZ – Helmholtz Centre for Environmental Research, Department Analytical Chemistry, Permoserstr. 15, D-04318 Leipzig, Germany

*Corresponding author: e-mail address: peter.herzsprung@ufz.de

Number of pages: 49

Number of figures: 31

Number of screenshots: 13

Number of tables: 7

Files:

es5c01986_si_001.pdf (this file, all figures, screenshots and tables as listed above)

es5c01986_si_002.xlsx (data of reactivity distribution and reactivity evaluation model comparison)

es5c01986_si_003.xlsx (molecular formula data, 9409 MF, with assigned reactivities and S/N(*p*) data

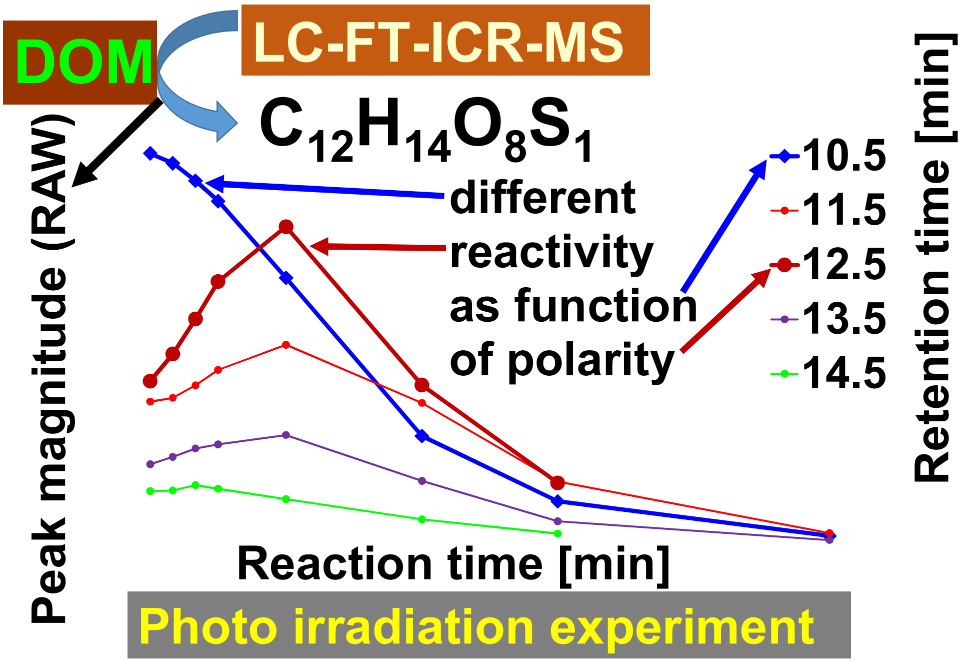

Contents

Nr	Title	Page
SI 1	Photo degradation experiment + study site	S5
Figure S1	Sampling at effluent of WWTP Silstedt	S5
Figure S2	UVACUBE 400 and the emitted spectrum of the irradiating lamp.	S6
Figure S3	Temperature as function of reaction time	S7
Table S1	DOC concentrations	S7
SI 2	Liquid chromatography coupled to Fourier transform ion cyclotron resonance mass spectrometry	S8
SI 2.1	Chromatography, mass spectrometry	S8
SI 2.2	Data processing	S8
SI 2.3	Data arrangement	S9
Table S2	Initial provided data structure from the UFZ data base	S9
Table S3	Calculation of pivot table mass versus sample number, output RAW value	S10
Table S4	Data arrangement required for evaluations of RAW as function of reaction time within each retention time segment	S10
SI 3	Analytical quality management using the two experimental replicates A and B	S11
Screensh. 1	Arrangement of data set for quality management calculations	S11
Screensh. 2	Calculation of relative differences of replicates A and B	S11
Screensh. 3	Excluding invalid RAW values and calculation of average RAW values	S12

Screensh. 4	Setting all invalid RAW values to S/N(p) < S/N(4) (displayed as “0”)	S12
Screensh. 5	Calculation of presence count (8 samples as function of reaction time)	S13
Table S5	Balance of valid and excluded RAW values	S13
SI 4	Calculation of parameters relevant for reactivity classification	S14
SI 4.1	Search for data gaps	S14
Screensh. 6	Presence counts (left) and calculated data gap free presence count (right)	S14
Screensh. 7	Presence counts list with marked data gaps	S15
Screensh. 8	Using marked data gaps list for assignment of *n.d.* and *r.n.a.* classes	S15
SI 4.2	Search for intermediate products	S16
Screensh. 9	Search for intermediate products	S16
Screens. 10	Assignment of MF to intermediate products	S17
SI 4.3	Calculation of relative (percentage) RAW differences δRAWs	S17
Screens. 11	Calculation of δRAW values, searching for the first end last valid RAW value	S18
SI 4.4	Final assignment of reactivity classes Prod, <Prod, *Degr*, *Degr*>, *Res*	S19
Screens. 12	Final assignment of reactivity classes	S19
SI 5	Examples for reaction time courses	S20
Figure S4	Examples for reaction time courses, Prod, <Prod, *Degr*, *Degr*>	S20
Figure S5	Examples for reaction time courses, *IntP*, <*IntP*>, <*IntP*, *IntP*>	S21
Figure S6	Examples for reaction time courses, *Res*, *r.n.a.*	S22
SI 6	Reactivity class balances	S23
Figure S7	Distribution of reactivity classes as function of retention time	S23
Figure S8	Comparison of reactivity classes distribution, DI 8P model versus LC model	S23
Figure S9	Calculated errors for counting the reactivity classes	S24
SI 7	Chemical distribution of DOM reactivity classes in van Krevelen diagrams	S25
Figure S10	Reactivity of MF, DI model, *Degr* and *Degr*> in vK diagrams	S25
Figure S11	Reactivity of MF, DI model, *Prod*, <*Prod*, *IntP*, <*IntP*>, <*IntP*, *IntP*> in vK diagrams	S26
Figure S12	Reactivity of MF, DI model, *Res*, *r.n.a.*, *n.d.* in vK diagrams	S27
Figure S13	Reactivity classes of MF, LC model in vK diagrams, RT = 5.5 min	S28
Figure S14	React. cl. of MF, LC model in vK diagrams, RT = 9.9 min	S29
Figure S15	React. cl. of MF, LC model in vK diagrams, RT = 10.5 min	S30
Figure S16	React. cl. of MF, LC model in vK diagrams, RT = 11.5 min	S31

Figure S17	React. cl. of MF, LC model in vK diagrams, RT = 12.5 min	S32
Figure S18	React. cl. of MF, LC model in vK diagrams, RT = 13.5 min	S33
Figure S19	React. cl. of MF, LC model in vK diagrams, RT = 14.5 min	S34
Figure S20	React. cl. of MF, LC model in vK diagrams, RT = 15.5 min	S35
Figure S21	React. cl. of MF, LC model in vK diagrams, RT = 16.5 min	S36
Figure S22	React. cl. of MF, LC model in vK diagrams, RT = 17.5 min	S37
Figure S23	React. cl. of MF, LC model in vK diagrams, RT = 18.5 min	S38
Figure S24	React. cl. of MF, LC model in vK diagrams, RT = 19.5 min	S39
Figure S25	React. cl. of MF, LC model in vK diagrams, RT = 20.5 min	S40
SI 8	Maximum RAW for *IntP*s as function of reaction time	S41
Screens. 13	Relation matrix between *IntP* maximum time point and different molecular descriptors	S41
Figure S26	*IntP* maximum time point in relation to H/C	S42
SI 9	Search for the opposite of *IntP*, an intermediate minimum	S43
Table S6	Number of MF showing an intermediate minimum	S43
Figure S27	MF showing intermediate minimum	S43
SI 10	Totalling opposite reactivity classes of the same MF	S44
Table S7	Which reactivity classes were found for MF, a balance	S44
Figure S28	Example for an MF showing five different reactivity classes for different RTs	S45
SI11	Degradation of potential surfactant metabolites	S45
Figure S29	Reaction time courses for potential linear alkylsulfonates metabolites	S46
Figure S30	Reaction time courses for potential dialkyl tetralin sulfonates metabolites	S47
SI12	Comparison to data sets from the literature	S48
Figure S31	Comparison of DI-FT-ICR-MS photo irradiation data from waste water and river water	S48
References		S49

SI 1 Study site and photo degradation experiment

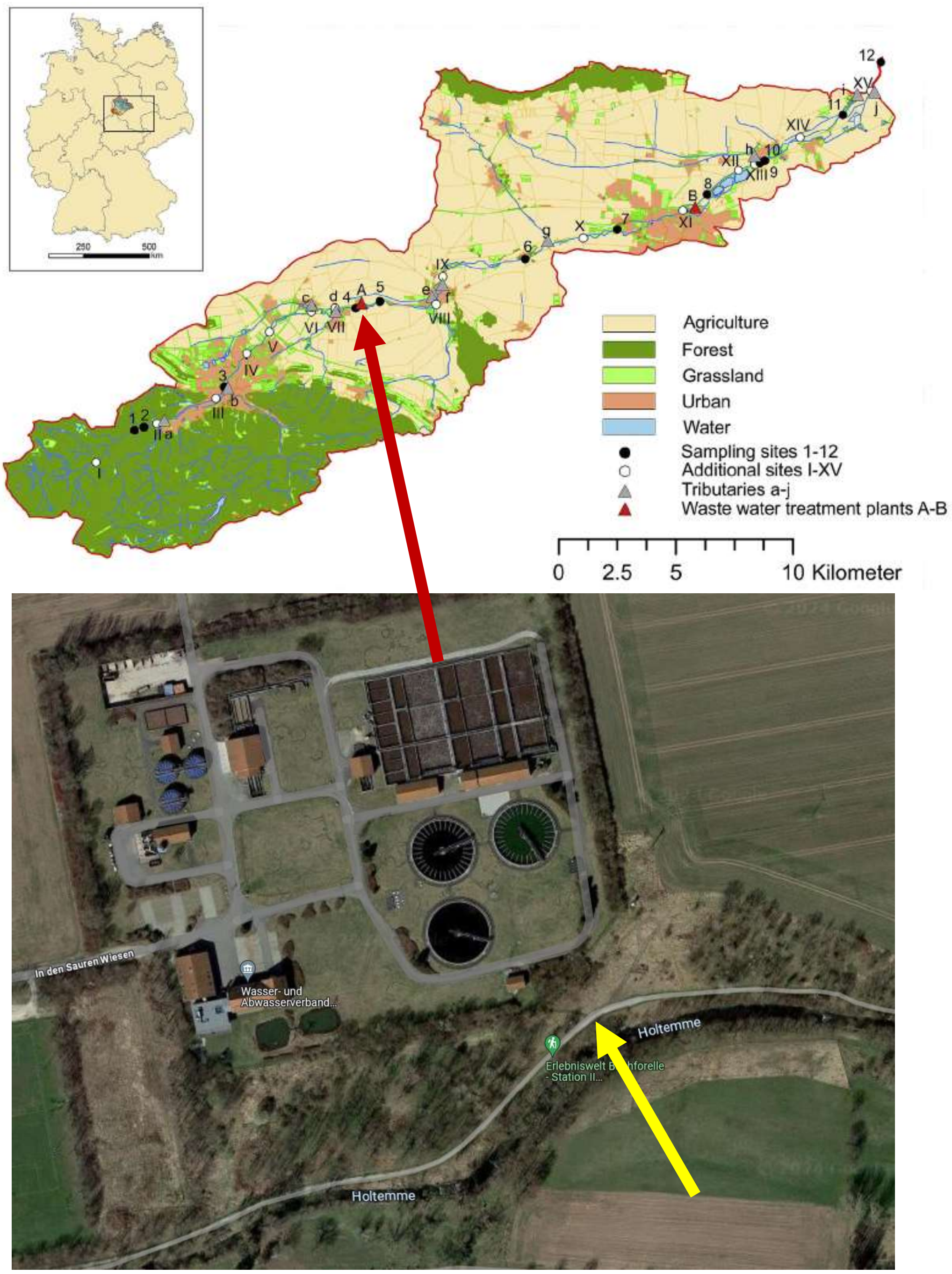

Fig. S1 Sampling at effluent of WWTP Silstedt, Saxony Anhalt, Germany

The experiments were performed in a one-liter quartz round bottom flask (further referred to as "reaction flask") with one liter sample mixed with 0.5 g/L of TiO2 powder (Degussa, P25, Evonik). The chosen catalyst concentration represents a balance under the reasons mentioned below:

1. The conversion rate of the reaction should be sufficient for the appropriate time limits of the experiment (a few hours)
2. The reaction should not proceed so quickly, so that the transformation of the educts (DOM) is complete only in a short period of time, otherwise little to no change of the species in the samples can be quantified nor qualified
3. The catalyst concentration should not exceed a certain threshold to cause such turbidity of the suspension, that would significantly impair the irradiation of the sample [1].

The TiO2 powder was first put to the flasks and the effluent-water was slowly added under stirring (using magnetic stir bar) to avoid agglomeration of the catalyst particles. The irradiating apparatus used was the "UVACUBE 400" from the company "Hönle". The apparatus consisted of a light-insulated cabinet, on top of which sat the lamp, simulating the natural sunlight, separated from the cabinet by a shutter-mechanism (Fig. S1). The provided irradiation dose is 28 mW/cm2 at the bottle bottom.

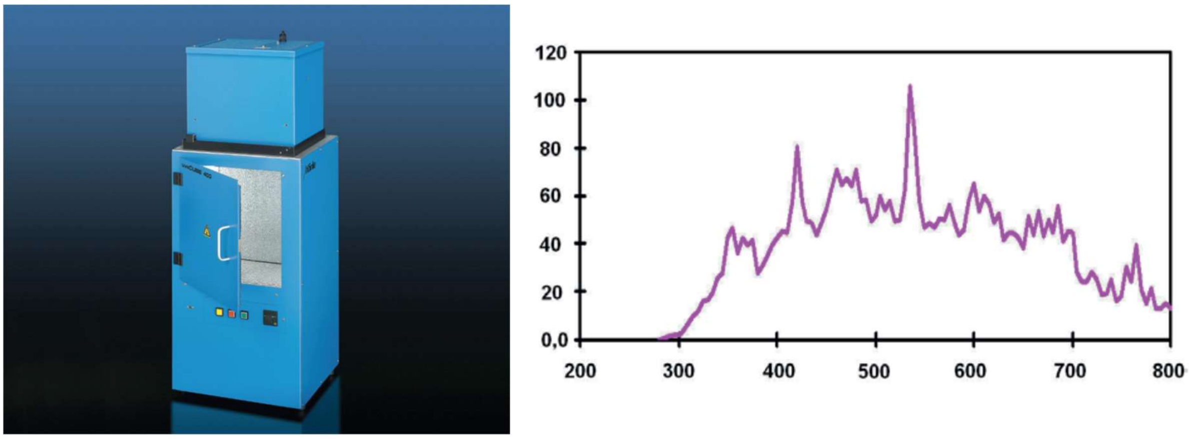

Fig. S2 UVACUBE 400 and the emitted spectrum of the irradiating lamp.

For each experiment a total of eight samples at the following timestamps were taken: 0 min, 10 min, 20 min, 30 min, 60 min, 120 min, 180 min and 300 min. The samples were taken directly from the reaction mixture with a syringe, then filtered through polyvinylidene fluoride (PVDF) syringe filters into small 60 mL brown-glass vials. Each filter was conditioned with a few mL of the sample water prior to the filling of the vials. The temperature readings were taken with a clean thermometer submerged directly into the vials. A total volume of the water extracted from the reaction flask was around 480 mL. It is important to note, that the catalyst was extracted from the reaction flask along with the effluent-water, which, given the perfect mixture, should not have increased the catalyst concentration as the reaction progressed.

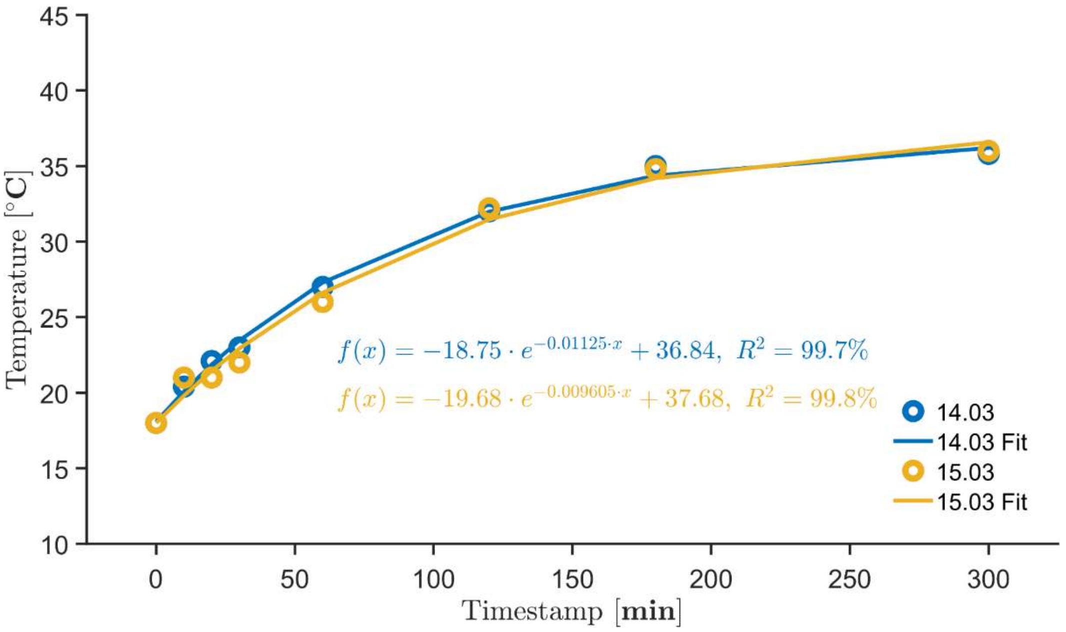

Fig. S3 Temperature as function of reaction time

About 15 mL of each sample were supplied for LC-FTICR – MS analysis at the Helmholtz-Zentrum Leipzig on the 17th of March 2022.

Table S1 DOC concentrations

Reaction time (min)		DOC (A) mg/L	DOC (B) mg/L
t_0	0	7.24	7.53
t_1	10	7.28	7.66
t_2	20	7.25	7.28
t_3	30	7.91	7.9
t_4	60	7.72	7.33
t_5	120	6.8	7.05
t_6	180	5.59	6.22
t_7	300	5.21	5.4

SI 2 Liquid chromatography coupled to Fourier transform ion cyclotron resonance mass spectrometry

SI 2.1 Chromatography, mass spectrometry

Samples were measured at their native concentrations without extraction to enable direct comparison of each sample. An ultra-high performance liquid chromatography (UHPLC) method employing a post-column counter-gradient was used as described previously (Han et al. 2020). Briefly, a reversed phase polar end-capped C18 column (ACQUITY HSS T3, 1.8 µm, 100 Å, 150 × 3 mm, Waters, Milford, U.S.A.) equipped with guard column (ACQUITY UPLC HSS T3 VanGuard, 100 Å, 1.8 µm, 2.1 mm × 5 mm, Waters) was used for the separation of DOM. Mobile phases were ultrapure water with 0.05% formic acid (FA) to reach pH 3 and methanol (MeOH; LC-MS-grade, Biosolve, Valkenswaard, Netherlands) to which the same amount of FA was added. In the counter gradient pump both mobile phases (MeOH and ultrapure water), were used without FA. Suwannee River Fulvic Acid (SRFA) from the International Humic Substances Society (SRFA II; 2S101F) and select model compounds were used for quality control of the LC system (Han et al. 2020).
An FT-ICR mass spectrometer equipped with a dynamically harmonized analyzer cell (solariX XR, Bruker Daltonics, Billerica, U.S.A.) and a 12 T refrigerated actively shielded superconducting magnet (Bruker Biospin, Wissembourg, France) was used for all LC-FT-ICR MS measurements in negative mode with electrospray ionization source (Apollo II, Bruker Daltonics). More details about the FT-ICR-MS settings for data acquisition can be found elsewhere (Han et al. 2020). The ion accumulation time (IAT) was set to 500 ms, and 2M data size was used to enhance peak resolution. The acquisition mass range was set to m/z 147-1000.

SI 2.2 Data processing

All full profile LC-FT-ICR MS chromatograms were segmented into 13 one-minute wide segments one at 5-6 min (covering the most polar DOM) and twelve between 9.7 and 21 minutes. Between 6 and 9.7 min, only little DOM eluted, resulting in mass spectra with only few DOM peaks. The segments were then processed and treated in the same way as DI-FT-ICR MS spectra as described previously (Han et al. 2020). Briefly, the signal to noise threshold was set to 4, and the spectra were internally recalibrated with a mass list of commonly found DOM masses (150 < m/z < 978; 8 < calibrants < 218, rmse < 0.28 ppm, n = 208). Mass spectral averaging and internal recalibration of segments was done in DataAnalysis 5.0 (Bruker Daltonics). After calibration, molecular formulas (MF) were calculated for the mass range 150 – 1000 Da with an error threshold of 1 ppm using the Lambda-Miner considering the following elements $^{12}C_{0-60}$, $^{13}C_{0-1}$, $^{1}H_{0-122}$, $^{16}O_{0-40}$, $^{14}N_{0-2}$, $^{32}S_{0-1}$, $^{34}S_{0-1}$.

Only formulas with 0.3 ≤ H/C ≤ 2.5, 0 ≤ O/C ≤ 1, 0 ≤ N/C ≤ 0.5, 0 ≤ DBE ≤ 20 (double bound equivalent, DBE = 1 + 1/2 (2C-H + N) and −10 ≤ DBE-O ≤ +10 were considered for further data evaluation. Isotopologue formulas (^{13}C, ^{34}S) were used for quality control but removed from the final data set as they represent duplicate chemical information.

SI 2.3 Data arrangement

The intensities were listed for each sample at each retention time with increasing mass. The retention time segments are: 5.51, 9.86, 10.5, 11.5, 12.5, 13.5, 14.5, 15.5, 16.5, 17.5, 18.5, 19.5, 20.5 min.

Table S2 Initial provided data structure from the UFZ data base

Sample ID	Sample data sub set number	Peak int	mass	formula	parameters calculated from mass and / or formula
t_0 5-6 Min	1	$RAW_1(m_1)$	m_1 (t_0)	$C_cH_hN_nO_oS_s$	DBE, AImod, NOSC, H/C, O/C…
…	…	$RAW_1(m_1)$	m_1 (t_0)		
t_0 20-21 Min	13	$RAW_1(m_1)$	m_1 (t_0)		
t_0 5-6 Min	1	$RAW_2(m_2)$	m_2 (t_0)		
…	…	$RAW_2(m_2)$	m_2 (t_0)		
t_0 20-21 Min	13	$RAW_2(m_2)$	m_2 (t_0)		
…	…	$RAW_x(m_x)$	m_x (t_0)		
t_1 5-6 Min	14	$RAW_x(m_x)$	m_x (t_1)		
…	…	…	…		
t_1 20-21 Min	26	…	…		
…	…	…	…		
…	…	…	…		
t_7 5-6 Min	92	$RAW_x(m_x)$	m_x (t_7)		
…		…	…		
t_7 20-21 Min	104	…	…		

rows only for mass peaks with S/N(p) > S/N(4)

Table S3 Calculation of pivot table mass versus sample number, output RAW value

mass	Sa 1	…	Sa 13	Sa 14	…	Sa 26	…	…	Sa 82	…	Sa 104
m_1	RAW_1 (m_1)	…	RAW_{13} (m_1)	RAW_{14} (m_1)	…	RAW_{26} (m_1)	…	…	RAW_{92} (m_1)	…	RAW_{104} (m_1)
m_2	RAW_1 (m_2)	…	RAW_{13} (m_2)	RAW_{14} (m_2)	…	RAW_{26} (m_2)	…	…	RAW_{92} (m_2)	…	RAW_{104} (m_2)
m_3	RAW_1 (m_3)	…	RAW_{13} (m_3)	RAW_{14} (m_3)	…	RAW_{26} (m_3)	…	…	RAW_{92} (m_3)	…	RAW_{104} (m_3)
…	…	…	…	…	…	…	…	…	…	…	…

In order to combine the intensities as function of reaction time, the samples are copied one below the other in a new data sheet. The masses are combined with the assigned formulas and calculated parameters (not shown). The complete data table is then first assorted to the mass and second to the reaction time. In that way the intensities can be compared retention time wise (for different sections) for the 8 reaction times in the columns.

Table S4 Data arrangement required for evaluations of RAW as function of reaction time within each retention time segment

	A or B		Retention time RT				
mass	sample data sub set	Reaction time	S1 5.5 MRAW	S2 9.9 MRAW	…	S12 19.5 MRAW	S13 20.5 MRAW
m_1	1-13	t_0	$RAW_{1}(m_1)$	$RAW_{2}(m_1)$	…	$RAW_{12}(m_1)$	$RAW_{13}(m_1)$
m_1	14-26	t_1	$RAW_{14}(m_1)$	$RAW_{15}(m_1)$	…	$RAW_{25}(m_1)$	$RAW_{26}(m_1)$
m_1	27-39	t_2	$RAW_{27}(m_1)$	$RAW_{28}(m_1)$	…	$RAW_{38}(m_1)$	$RAW_{39}(m_1)$
m_1	40-52	t_3	$RAW_{40}(m_1)$	$RAW_{41}(m_1)$	…	$RAW_{51}(m_1)$	$RAW_{52}(m_1)$
m_1	53-65	t_4	$RAW_{53}(m_1)$	$RAW_{54}(m_1)$	…	$RAW_{64}(m_1)$	$RAW_{65}(m_1)$
m_1	66-78	t_5	$RAW_{66}(m_1)$	$RAW_{67}(m_1)$	…	$RAW_{77}(m_1)$	$RAW_{78}(m_1)$
m_1	79-91	t_6	$RAW_{79}(m_1)$	$RAW_{80}(m_1)$	…	$RAW_{90}(m_1)$	$RAW_{91}(m_1)$
m_1	92-104	t_7	$RAW_{92}(m_1)$	$RAW_{93}(m_1)$	…	$RAW_{103}(m_1)$	$RAW_{104}(m_1)$
m_2	1-13	t_0	$RAW_{1}(m_2)$	$RAW_{2}(m_2)$	…	$RAW_{12}(m_2)$	$RAW_{13}(m_2)$
m_2	14-26	t_1	$RAW_{14}(m_2)$	$RAW_{15}(m_2)$	…	$RAW_{25}(m_2)$	$RAW_{26}(m_2)$
m_2	27-39	t_2	$RAW_{27}(m_2)$	$RAW_{28}(m_2)$	…	$RAW_{38}(m_2)$	$RAW_{39}(m_2)$
m_2	40-52	t_3	$RAW_{40}(m_2)$	$RAW_{41}(m_2)$	…	$RAW_{51}(m_2)$	$RAW_{52}(m_2)$
m_2	53-65	t_4	$RAW_{53}(m_2)$	$RAW_{54}(m_2)$	…	$RAW_{64}(m_2)$	$RAW_{65}(m_2)$
m_2	66-78	t_5	$RAW_{66}(m_2)$	$RAW_{67}(m_2)$	…	$RAW_{77}(m_2)$	$RAW_{78}(m_2)$
m_2	79-91	t_6	$RAW_{79}(m_2)$	$RAW_{80}(m_2)$	…	$RAW_{90}(m_2)$	$RAW_{91}(m_2)$
m_2	92-104	t_7	$RAW_{92}(m_2)$	$RAW_{93}(m_2)$	…	$RAW_{103}(m_2)$	$RAW_{104}(m_2)$
m_3	…	…	…	…	…	…	…
…	…	…	…	…	…	…	…

SI 3 Analytical quality management using the two experimental replicates A and B

The data sets are assorted in separate for the replicates A and B.

Screenshot 1 Arrangement of data set for quality management calculations

A	B	C	D	E	F	G	H	I	J	K	L	M	N	O	P
parameters			replicate A							replicate B					
mass	formula	t(min)	5.5	9.9	10.5	11.5	12.5	13.5	...	5.5	9.9	10.5	11.5	12.5	13.5
292.13	C11 H20 N2 O7	0	12878	69889.1	66162.7	14648.1	0	0	...	11550	89369	70460.4	15547.4	16129.6	17817.8
292.13	C11 H20 N2 O7	10	13602	65613.1	74227	15830.6	16399.8	14027	...	13591.5	67843.8	74412.4	15687.6	0	14787.9
292.13	C11 H20 N2 O7	20	16080.6	84531.9	85531.6	17644.3	0	0	...	15624.6	80905.4	82651.4	18346.4	17040.2	18300.5
292.13	C11 H20 N2 O7	30	15491.8	109675	97053.9	20052.6	16935.3	17887.6	...	14763.7	92383.4	96860.7	19531.9	16781.9	17522.2
292.13	C11 H20 N2 O7	60	20062.6	138958	124283	27503.1	22307.4	19729.2	...	16000.4	102445	123939	28073.3	20697.6	28286
292.13	C11 H20 N2 O7	120	13969.1	130942	147740	33521.8	21922	0	...	0	135867	160046	30990.2	21799.1	18418.8
292.13	C11 H20 N2 O7	180	0	154572	125176	29738.4	19913	13169	...	0	155906	123203	30850.2	19507.5	19774.7
292.13	C11 H20 N2 O7	300	0	116019	95193.2	0	13099.5	15252.3	...	0	103210	91119.8	0	13215.4	0

For each combination A and B as function of mass, retention time RT and reaction time t the percentage deviation from the average is calculated from the intensities:

Eq.1: Relative Difference (A;B) = δRAW_A

$$\frac{|A - B| \cdot 100\,\%}{(A + B) / 2}$$

This value is only calculated under the condition that both replicate A and B show S/N(p) values > S/N(4) at the same position in the data matrix. If S/N(A) < S/N(4) and S/N(B) > S/N(4), then it is displayed: A=0_B>0. If S/N(B) < S/N(4) and S/N(A) > S/N(4), then it is displayed: A>0_B=0. If both replicates S/N(A) < S/N(4) and S/N(B) < S/N(4), then it is displayed: A,B = 0.

Screenshot 2 Calculation of relative differences of replicates A and B

A	B	C	D	E	F	G	H	I	J
parameters			relative differences replicates A, B [%]						
mass	formula	t(min)	5.5	9.9	10.5	11.5	12.5	13.5	...
292.13	C11 H20 N2 O7	0	10.87	24.46	6.29	5.96	A=0_B>0	A=0_B>0	
292.13	C11 H20 N2 O7	10	0.08	3.34	0.25	0.91	A>0_B=0	5.28	
292.13	C11 H20 N2 O7	20	2.88	4.38	3.43	3.90	A=0_B>0	A=0_B>0	
292.13	C11 H20 N2 O7	30	4.81	17.12	0.20	2.63	0.91	2.06	
292.13	C11 H20 N2 O7	60	22.53	30.25	0.28	2.05	7.49	35.64	
292.13	C11 H20 N2 O7	120	A>0_B=0	3.69	8.00	7.85	0.56	A=0_B>0	
292.13	C11 H20 N2 O7	180	A,B = 0	0.86	1.59	3.67	2.06	40.10	
292.13	C11 H20 N2 O7	300	A,B = 0	11.69	4.37	A,B = 0	0.88	A>0_B=0	

Of all available relative differences the 95 percentile is calculated:
=QUANTIL.INKL($B$2:$N$189785;95/100)

The result was 0.265 = 26.5%

Screenshot 3 Excluding invalid RAW values and calculation of average RAW values

=IF(Quantil!D2<0.265;(replicates!D2+Q2)/2);IF(Quantil!D2>0.265;"exclude";Quantil!D2))

(D2+Q2)/2): average value for the replicates A and B.

A	B	C	D	E	F	G	H	I	J
parameters			average intensity value A, B						
mass	formula	t(min)	5.5	9.9	10.5	11.5	12.5	13.5	...
292.13	C11 H20 N2 O7	0	12214	79629	68312	15098	A=0_B>0	A=0_B>0	
292.13	C11 H20 N2 O7	10	13597	66728	74320	15759	A>0_B=0	14407	
292.13	C11 H20 N2 O7	20	15853	82719	84092	17995	A=0_B>0	A=0_B>0	
292.13	C11 H20 N2 O7	30	15128	101029	96957	19792	16859	17705	
292.13	C11 H20 N2 O7	60	18032	exclude	124111	27788	21503	exclude	
292.13	C11 H20 N2 O7	120	A>0_B=0	133405	153893	32256	21861	A=0_B>0	
292.13	C11 H20 N2 O7	180	A,B = 0	155239	124189	30294	19710	exclude	
292.13	C11 H20 N2 O7	300	A,B = 0	109614	93157	A,B = 0	13157	A>0_B=0	

This scheme shows all excluded values. If the relative difference A;B is smaller than the 95 percentile, then we consider the replicate measurement for this data pair as valid and display the average value. If the difference is larger than the 95 percentile, then the data pair is excluded in the data matrix as shown above. All the A>0_B=0 / A=0_B>0 values are considered as well as invalid and are excluded. If a data pair is A,B=0, then it is insofar valid as both A and B replicates are under the limit of detection (< S/N(4)).
For simplification we regard all these values A>0_B=0 / A=0_B>0, A,B=0, “exclude” as invalid and set them as <S/N(4) (displayed as “0” or text depending on the calculation usage). This setting gives the corresponding cell the status of “absent”. The valid values receive the status “present”. This perspective is necessary for calculation of presence counts for each MF at each RT.

Screenshot 4 Setting all invalid RAW values to S/N(p) < S/N(4) (displayed as “0”)

A	B	C	D	E	F	G	H	I	J
parameters			average intensity value A, B						
mass	formula	t(min)	5.5	9.9	10.5	11.5	12.5	13.5	...
292.13	C11 H20 N2 O7	0	12214	79629	68312	15098	0	0	
292.13	C11 H20 N2 O7	10	13597	66728	74320	15759	0	14407	
292.13	C11 H20 N2 O7	20	15853	82719	84092	17995	0	0	
292.13	C11 H20 N2 O7	30	15128	101029	96957	19792	16859	17705	
292.13	C11 H20 N2 O7	60	18032	0	124111	27788	21503	0	
292.13	C11 H20 N2 O7	120	0	133405	153893	32256	21861	0	
292.13	C11 H20 N2 O7	180	0	155239	124189	30294	19710	0	
292.13	C11 H20 N2 O7	300	0	109614	93157	0	13157	0	

The next step is to calculate the presence count in the matrix which contains the average RAW values and the <S/N(4) values.

Screenshot 5 Calculation of presence count (8 samples as function of reaction time)

A	B	C	D	E	F	G	H	I	J
parameters			presence count						
mass	formula	t(min)	5.5	9.9	10.5	11.5	12.5	13.5	...
292.127051	C11 H20 N2 O7	0	5	7	8	7	5	2	
292.127051	C11 H20 N2 O7	10	5	7	8	7	5	2	
292.127051	C11 H20 N2 O7	20	5	7	8	7	5	2	
292.127051	C11 H20 N2 O7	30	5	7	8	7	5	2	
292.127051	C11 H20 N2 O7	60	5	7	8	7	5	2	
292.127051	C11 H20 N2 O7	120	5	7	8	7	5	2	
292.127051	C11 H20 N2 O7	180	5	7	8	7	5	2	
292.127051	C11 H20 N2 O7	300	5	7	8	7	5	2	

For each MF the maximum of presence counts is then calculated. If the maximum presence count is smaller than 2, then it makes evidently no sense to consider the corresponding component for reactivity evaluation (calculation of slopes or relative RAW differences, see literature). Hence all MF with maximum presence count < 2 (in 8 reaction time dependent samples) were excluded from further calculations. Here the maximum presence count = 8.

Table S5 Balance of valid and excluded RAW values

Retention time [min]	total (as a control)	valid values	A,B=0	additionally set as < S/N(4)	A=0_B>0	A>0_B=0	excluded δRAW_{AB} > 0.265
5.5	129784	7462	116391	5931	2567	3260	104
9.9	129784	14944	105966	8874	3644	4005	1225
10.5	129784	22125	99021	8638	3977	4480	181
11.5	129784	21912	99382	8490	4232	3995	263
12.5	129784	21681	99386	8717	4106	4179	432
13.5	129784	23010	97545	9229	4417	4229	583
14.5	129784	18737	100154	10893	5256	4239	1398
15.5	129784	18253	101640	9891	4493	4551	847
16.5	129784	17379	102999	9406	4385	4073	948
17.5	129784	10962	109243	9579	4235	4368	976
18.5	129784	11869	108992	8923	4170	3536	1217
19.5	129784	9184	111807	8793	4273	2962	1558
20.5	129784	2554	118107	9123	3728	4596	799
total		200072	1370633	116487	53483	52473	10531

Additionally set as < S/N(4) column means the sum of A>0_B=0 + A=0_B>0 + "excluded"; A,B=0 means S/N(A,B) < S/N(4); A=0_B>0 means S/N(A) < S/N(4), S/N(B) > S/N(4); A>0_B=0 means S/N(A) > S/N(4), S/N(B) < S/N(4)

SI 4 Calculation of parameters relevant for reactivity classification

SI 4.1 Search for data gaps

For each MF and each RT it is searched if 2 RAW values at t_x and t_{x+1} exist with S/N(p). > S/N(4).

The search begins with RAW ($t_0^{\rightarrow}$ and $t_1^{\rightarrow}$) and ends with RAW ($t_6^{\rightarrow}$ and $t_7^{\rightarrow}$). If any of such RAW pairs is found then the cell value is "1", otherwise "#value!" The name of the search table is "search2"

For 8 $t_x^{\rightarrow}$ 8 cells are calculated. In a second data sheet the presence counts (see screenshot 4) are prepared.

A new data sheet is created in order to check if the presence count is 2 and the value of "search2" is 1

If(and(pres!D2=2;MAX(search2!D2:D9)=1);2;0).

The search is continued by proofing if 3 RAW values exist at t_x and t_{x+1} and t_{x+2} with S/N(p) > S/N(4)

➔ If(and(pres!D2=3;MAX(search3!D2:D9)=1);2;0)

The search is continued with proof for 4, 5, 6, 7 data points without data gap.

…

➔ If(and(pres!D2=7;MAX(search3!D2:D9)=1);7;0)

All these data are combined in one new data sheet ("pres*"). For search the 7 calculated (above described) data sheets are used and the presence count data sheet. Presence count 8 means that there cannot exist a data gap.

=IF(pres!C2=8;8;IF('2'!C2=2;2;IF('3'!C2=3;3;IF('4'!C2=4;4;IF('5'!C2=5;5;IF('6'!C2=6;6;IF('7'!C2=7;7;0)))))))

The pres* data sheet contains only presence counts where no data gap exists.

Screenshot 6 presence counts (left) and calculated data gap free presence count (right)

A	B	C	D	E	F	G	H	I	J	K	L	M	N	O	P
parameters			presence count							presence count, free of data gaps					
mass	formula	t(min)	5.5	9.9	10.5	11.5	12.5	13.5	...	5.5	9.9	10.5	11.5	12.5	13.5
292.127051	C11 H20 N2 O7	0	5	7	8	7	5	2		5	0	8	7	5	0
292.127051	C11 H20 N2 O7	10	5	7	8	7	5	2		5	0	8	7	5	0
292.127051	C11 H20 N2 O7	20	5	7	8	7	5	2		5	0	8	7	5	0
292.127051	C11 H20 N2 O7	30	5	7	8	7	5	2		5	0	8	7	5	0
292.127051	C11 H20 N2 O7	60	5	7	8	7	5	2		5	0	8	7	5	0
292.127051	C11 H20 N2 O7	120	5	7	8	7	5	2		5	0	8	7	5	0
292.127051	C11 H20 N2 O7	180	5	7	8	7	5	2		5	0	8	7	5	0
292.127051	C11 H20 N2 O7	300	5	7	8	7	5	2		5	0	8	7	5	0

Both tables, presence count and “presence, free of data gaps” are mathematically compared (simply using the differences of cell values) and combined in one data matrix where all presence counts are marked with an asterisk, if a data gap has been found.

Screenshot 7 presence counts list with marked data gaps

A	B	C	D	E	F	G	H	I	J	K	L	M	N	O	P
parameters			modified presence count							replicate corrected data set					
mass	formula	t(min)	5.5	9.9	10.5	11.5	12.5	13.5	...	5.5	9.9	10.5	11.5	12.5	13.5
292.127051	C11 H20 N2 O7	0	5	7 *	8	7	5	2 *		12214	79629.1	68311.6	15097.8	0	0
292.127051	C11 H20 N2 O7	10	5	7 *	8	7	5	2 *		13596.8	66728.5	74319.7	15759.1	0	14407.5
292.127051	C11 H20 N2 O7	20	5	7 *	8	7	5	2 *		15852.6	82718.7	84091.5	17995.4	0	0
292.127051	C11 H20 N2 O7	30	5	7 *	8	7	5	2 *		15127.8	101029	96957.3	19792.3	16858.6	17704.9
292.127051	C11 H20 N2 O7	60	5	7 *	8	7	5	2 *		18031.5	0	124111	27788.2	21502.5	0
292.127051	C11 H20 N2 O7	120	5	7 *	8	7	5	2 *		0	133405	153893	32256	21860.6	0
292.127051	C11 H20 N2 O7	180	5	7 *	8	7	5	2 *		0	155239	124189	30294.3	19710.3	0
292.127051	C11 H20 N2 O7	300	5	7 *	8	7	5	2 *		0	109614	93156.5	0	13157.5	0

All MF were set as *r.n.a.* for RTs where a data gap was found. This can be simply done because the marked cells are regarded as text by the excel software.
Finally the first two reactivity classes can be assigned:
If the presence count is 0 → *n.d.*
If in the cell a number is found → display number (presence count free of data gaps)
If not, then display cell content → *r.n.a.*

Screenshot 8 using marked data gaps list for assignment of *n.d.* and *r.n.a.* classes

A	B	C	D	E	F	G	H	I	J	K	L	M	N	O	P	Q
parameters			modified presence count							assigned reactivity class						
mass	formula	t(min)	5.5	9.9	10.5	11.5	12.5	13.5	14.5	5.5	9.9	10.5	11.5	12.5	13.5	14.5
292.127051	C11 H20 N2 O7	0	5	7 *	8	7	5	2 *	0	5	r.n.a.	8	7	5	r.n.a.	n.d.
292.127051	C11 H20 N2 O7	10	5	7 *	8	7	5	2 *	0	5	r.n.a.	8	7	5	r.n.a.	n.d.
292.127051	C11 H20 N2 O7	20	5	7 *	8	7	5	2 *	0	5	r.n.a.	8	7	5	r.n.a.	n.d.
292.127051	C11 H20 N2 O7	30	5	7 *	8	7	5	2 *	0	5	r.n.a.	8	7	5	r.n.a.	n.d.
292.127051	C11 H20 N2 O7	60	5	7 *	8	7	5	2 *	0	5	r.n.a.	8	7	5	r.n.a.	n.d.
292.127051	C11 H20 N2 O7	120	5	7 *	8	7	5	2 *	0	5	r.n.a.	8	7	5	r.n.a.	n.d.
292.127051	C11 H20 N2 O7	180	5	7 *	8	7	5	2 *	0	5	r.n.a.	8	7	5	r.n.a.	n.d.
292.127051	C11 H20 N2 O7	300	5	7 *	8	7	5	2 *	0	5	r.n.a.	8	7	5	r.n.a.	n.d.

SI 4.2 Search for intermediate products

For this search the S/N data are required from the FTICR-MS raw data tables. Note that only signals with S/N(p) > S/N(4) have been considered for RAW display. The S/N data table was in the same manner modified as the RAW data table (proofing A,B replicates, search for data gaps).

IntP: If the presence count is 8, it is proofed (like for RT = 10.5 min in screenshot 9, column N):

1. δRAW_{max} = [max RAW(t_1- t_6) - RAW(t_0)] / (RAW(t_0) > 0.265
2. δRAW_{max} = [max RAW(t_1- t_6) - RAW(t_7)] / (RAW(t_7) > 0.265

δRI_{max} is calculated as shown RAW screenshot 9.

Screenshot 9 search for intermediate products (*IntPs*)

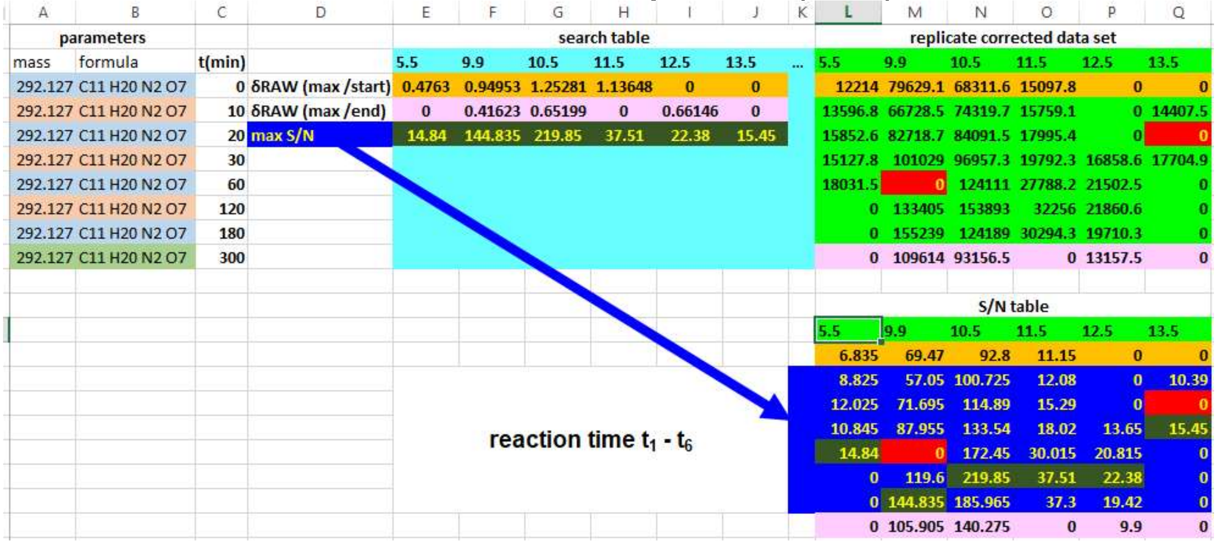

A	B	C	D	E	F	G	H	I	J	K	L	M	N	O	P	Q
parameters				search table							replicate corrected data set					
mass	formula	t(min)		5.5	9.9	10.5	11.5	12.5	13.5	...	5.5	9.9	10.5	11.5	12.5	13.5
292.127	C11 H20 N2 O7	0	δRAW (max /start)	0.4763	0.94953	1.25281	1.13648	0	0		12214	79629.1	68311.6	15097.8	0	0
292.127	C11 H20 N2 O7	10	δRAW (max /end)	0	0.41623	0.65199	0	0.66146	0		13596.8	66728.5	74319.7	15759.1	0	14407.5
292.127	C11 H20 N2 O7	20	max S/N	14.84	144.835	219.85	37.51	22.38	15.45		15852.6	82718.7	84091.5	17995.4	0	0
292.127	C11 H20 N2 O7	30									15127.8	101029	96957.3	19792.3	16858.6	17704.9
292.127	C11 H20 N2 O7	60									18031.5	0	124111	27788.2	21502.5	0
292.127	C11 H20 N2 O7	120									0	133405	153893	32256	21860.6	0
292.127	C11 H20 N2 O7	180									0	155239	124189	30294.3	19710.3	0
292.127	C11 H20 N2 O7	300									0	109614	93156.5	0	13157.5	0

S/N table

	L	M	N	O	P	Q
	5.5	9.9	10.5	11.5	12.5	13.5
	6.835	69.47	92.8	11.15	0	0
reaction time t_1 - t_6	8.825	57.05	100.725	12.08	0	10.39
	12.025	71.695	114.89	15.29	0	0
	10.845	87.955	133.54	18.02	13.65	15.45
	14.84	0	172.45	30.015	20.815	0
	0	119.6	219.85	37.51	22.38	0
	0	144.835	185.965	37.3	19.42	0
	0	105.905	140.275	0	9.9	0

<*IntP*>, <*IntP*, *IntP*>: If the presence count is < 8, it is proofed (like for column L,O,P):

1. S/N(p)(t_0) < S/N(4), S/N(p)(t_7) < S/N(4)
2. max S/N(p)(t_1- t_6) > 12 → <*IntP*>
3. S/N(p)(t_0) < S/N(4), S/N(p)(t_7) > S/N(4)
4. max S/N(p)(t_1- t_6) > 12
5. δRAW_{max} = [max RAW(t_1- t_6) - RAW(t_7)] / (RAW(t_7) > 0.265 → <*IntP*
6. S/N(p)(t_0) > S/N(4), S/N(p)(t_7) < S/N(4)
7. max S/N(p)(t_1- t_6) > 12
8. δRAW_{max} = [max RAW(t_1- t_6) - RAW(t_0)] / (RAW(t_0) > 0.265 → *IntP*>

Security factor: > 12: means > three times S/N(4)
As a rule of thumb the limit of quantification is about three times the limit of detection.

The reactivity classes *n.d.* and *r.n.a.* are already assigned. With calculation for criteria of intermediate products the classes *IntP*, < *IntP* >, < *IntP* and *IntP* > can now be assigned as follows.

Screenshot 10 assignment of MF to intermediate products

A	B	C	D	E	F	G	H	I	J	K	L	M	N	O	P	Q
parameters			assigned reactivity class							replicate corrected data set						
mass	formula	t(min)	5.5	9.9	10.5	11.5	12.5	13.5	14.5	5.51	9.86	10.5	11.5	12.5	13.5	14.5
292.13	C11 H20 N2 O7	0	IntP>	r.n.a.	IntP	IntP>	<IntP	r.n.a.	n.d.	12214	79629	68312	15098	0	0	0
292.13	C11 H20 N2 O7	10	IntP>	r.n.a.	IntP	IntP>	<IntP	r.n.a.	n.d.	13597	66728	74320	15759	0	14407	0
292.13	C11 H20 N2 O7	20	IntP>	r.n.a.	IntP	IntP>	<IntP	r.n.a.	n.d.	15853	82719	84092	17995	0	0	0
292.13	C11 H20 N2 O7	30	IntP>	r.n.a.	IntP	IntP>	<IntP	r.n.a.	n.d.	15128	101029	96957	19792	16859	17705	0
292.13	C11 H20 N2 O7	60	IntP>	r.n.a.	IntP	IntP>	<IntP	r.n.a.	n.d.	18032	0	124111	27788	21503	0	0
292.13	C11 H20 N2 O7	120	IntP>	r.n.a.	IntP	IntP>	<IntP	r.n.a.	n.d.	0	133405	153893	32256	21861	0	0
292.13	C11 H20 N2 O7	180	IntP>	r.n.a.	IntP	IntP>	<IntP	r.n.a.	n.d.	0	155239	124189	30294	19710	0	0
292.13	C11 H20 N2 O7	300	IntP>	r.n.a.	IntP	IntP>	<IntP	r.n.a.	n.d.	0	109614	93157	0	13157	0	0

SI 4.3 Calculation of relative (percentage) RAW differences δRAWs

The δRAW values are calculated as described by Herzsprung et al:
δRAW = [RAW (sample X) – RAW (sample Y)] / RAW (sample Y)
where sample Y is the start sample and sample X is the end sample.
δRAW =

$$\delta RAW_i^{End} = \frac{RAW_i^{End} - RAW_i^{Start}}{RAW_i^{Start}}$$

The calculation is adapted to a kinetic (function of time) sample series with no necessary total common presence of MF. Beginning with t = 0 Min, the first sample with S/N(p) > S/N(4) is searched and taken as start sample (RAW_i^{Start}). In opposite direction, beginning with t = 300 Min, the first sample with S/N(p) > S/N(4) is searched and taken as end sample (RAW_i^{End}).

RAW_i^{Start}=E3=IF(table!L3>0;table!L3;IF(table!L4>0;table!L4;IF(table!L5>0;table!L5;IF(table!L6>0;table!L6;IF(table!L7>0;table!L7;IF(table!L8>0;table!L8;IF(table!L9>0;table!L9;L10)))))))
RAW_i^{End}=E4=IF(table!L10>0;table!L10;IF(table!L9>0;table!L9;IF(table!L8>0;table!L8;IF(table!L7>0;table!L7;IF(table!L6>0;table!L6;IF(table!L5>0;table!L5;IF(table!L4>0;table!L4;L3)))))))
δRAW=E5=((E4-E3)/E3)

Screenshot 11 Calculation of δRAW values, searching for the first end last valid RAW value

A	B	C	D	E	F	G	H	I	J	K	L	M	N	O	P	Q	R
parameters				search table							replicate corrected data set						
mass	formula	t(min)		5.5	9.9	10.5	11.5	12.5	13.5	14.5	5.5	9.9	10.5	11.5	12.5	13.5	14.5
174.1	C7 H10 O5	0	start RAW	15062	31747	49407	125676	76140	26945	14517	15062	31747	49407	0	76140	26945	14517
174.1	C7 H10 O5	10	end RAW	18638	78322	239958	829864	548167	299568	31203	16572	40191	78357	125676	95311	77782	15372
174.1	C7 H10 O5	20	δRAW	0.2374	1.4671	3.8568	5.6032	6.1995	10.118	1.1494	16385	0	95094	170210	118150	92875	18276
174.1	C7 H10 O5	30									18039	55541	115446	208921	145686	96161	16445
174.1	C7 H10 O5	60									18638	69745	169950	350819	247718	145923	21146
174.1	C7 H10 O5	120									0	95463	224798	651000	439575	257796	26683
174.1	C7 H10 O5	180									0	99677	218402	690422	548167	312597	26889
174.1	C7 H10 O5	300									0	78322	239958	829864	0	299568	31203
274.1	C11 H14 O8	0	start RAW	35162	268359	517163	655406	549140	216880	60240	35162	268359	517163	655406	549140	216880	60240
274.1	C11 H14 O8	10	end RAW	44024	147773	415806	511974	412178	253877	78316	40408	276296	552385	726814	581934	239147	67183
274.1	C11 H14 O8	20	δRAW	0.252	-0.449	-0.196	-0.219	-0.249	0.1706	0.3001	42015	288588	581038	799177	623295	265655	71540
274.1	C11 H14 O8	30									41090	330828	622287	822200	624060	262904	69684
274.1	C11 H14 O8	60									44024	323582	691543	898450	685274	287283	72064
274.1	C11 H14 O8	120									0	296479	684700	902028	689784	299020	91635
274.1	C11 H14 O8	180									0	259586	543517	735403	638297	284662	85171
274.1	C11 H14 O8	300									0	147773	415806	511974	412178	253877	78316
292.1	C11 H20 N2 O7	0	start RAW	12214	79629	68312	15098	16859	14407	0	12214	79629	68312	15098	0	0	0
292.1	C11 H20 N2 O7	10	end RAW	18032	109614	93157	30294	13157	17705	0	13597	66728	74320	15759	0	14407	0
292.1	C11 H20 N2 O7	20	δRAW	0.4763	0.3766	0.3637	1.0065	-0.22	0.2289	DIV/0!	15853	82719	84092	17995	0	0	0
292.1	C11 H20 N2 O7	30									15128	101029	96957	19792	16859	17705	0
292.1	C11 H20 N2 O7	60									18032	0	124111	27788	21503	0	0
292.1	C11 H20 N2 O7	120									0	133405	153893	32256	21861	0	0
292.1	C11 H20 N2 O7	180									0	155239	124189	30294	19710	0	0
292.1	C11 H20 N2 O7	300									0	109614	93157	0	13157	0	0

If only one value from 8 time points is larger than 0, then $RAW_i^{End} = RAW_i^{Start}$ and δRAW=0

If no S/N(p) from 8 time points is larger than S/N(4), then #DIV/0! will be displayed.

Both cases δRAW=0 and #DIV/0! will be generally disregarded for further calculations or evaluations and the corresponding cells set as text as needed.

The search is independently of existing data gaps. The δRAW values will be used for the final search for products, degraded and resistant MF. The classes *n.d.*, *r.n.a.*, and intermediate products are searched previously and will be marked in the search table. Only MF which are not member of the previously searched classes are proofed for being product, degraded or resistant.

This search strategy offers the advantage that excel equations can be copied throughout the data tables for the δRAW values calculation.

SI 4.4 Final assignment of reactivity classes *Prod*, *<Prod*, *Degr*, *Degr>*, *Res*

The reactivity classes are finally completed. As shown in Screenshot 10, the classes *IntP*, < *IntP* >, < *IntP*, *IntP* >, n.r.a., *n.d.* were assigned first. Only MF with no assignment are further proofed for the classes *Prod*, *<Prod*, *Degr*, *Degr>*, *Res*. The δRAW values shown in Screenshot 11 are used for assignment of these classes. If -0.265 < δRAW < 0.265, then it is finally proofed, if RAW_{max} > 12 (three times S/N(4)).
If δRAW < -0.265; → *Degr*
If -0.265 < δRAW < 0.265 and S/N(p)(t_7) < S/N(4) and max S/N > 12; → *Degr>*
If -0.265 < δRAW < 0.265 and S/N(p)(t_7) < S/N(4) and max S/N(p) < 12; → *Res*
If δRAW > 0.265; → *Prod*
If -0.265 < δRAW < 0.265 and S/N(p)(t_0) < S/N(4) and max S/N(p) > 12; → *<Prod*
If -0.265 < δRAW < 0.265 and S/N(p)(t_0) < S/N(4) and max S/N(p) < 12; → *Res*
If -0.265 < δRAW < 0.265 and presence count = 8; → *Res*

Screenshot 12 Final assignment of reactivity classes

A	B	C	D	E	F	G	H	I	J	K	L	M	N	O	P	Q	R
parameters				search table							replicate corrected data set						
mass	formula	t(min)		5.5	9.9	10.5	11.5	12.5	13.5	14.5	5.5	9.9	10.5	11.5	12.5	13.5	14.5
174.1	C7 H10 O5	0	start RAW	Degr>	r.n.a.	Prod	Prod	IntP>	Prod	Prod	15062	31747	49407	0	76140	26945	14517
174.1	C7 H10 O5	10	end RAW	Degr>	r.n.a.	Prod	Prod	IntP>	Prod	Prod	16572	40191	78357	125676	95311	77782	15372
174.1	C7 H10 O5	20	δRAW	Degr>	r.n.a.	Prod	Prod	IntP>	Prod	Prod	16385	0	95094	170210	118150	92875	18276
174.1	C7 H10 O5	30		Degr>	r.n.a.	Prod	Prod	IntP>	Prod	Prod	18039	55541	115446	208921	145686	96161	16445
174.1	C7 H10 O5	60		Degr>	r.n.a.	Prod	Prod	IntP>	Prod	Prod	18638	69745	169950	350819	247718	145923	21146
174.1	C7 H10 O5	120		Degr>	r.n.a.	Prod	Prod	IntP>	Prod	Prod	0	95463	224798	651000	439575	257796	26683
174.1	C7 H10 O5	180		Degr>	r.n.a.	Prod	Prod	IntP>	Prod	Prod	0	99677	218402	690422	548167	312597	26889
174.1	C7 H10 O5	300		Degr>	r.n.a.	Prod	Prod	IntP>	Prod	Prod	0	78322	239958	829864	0	299568	31203
274.1	C11 H14 O8	0	start RAW	Degr>	Degr	IntP	IntP	Res	Res	Prod	35162	268359	517163	655406	549140	216880	60240
274.1	C11 H14 O8	10	end RAW	Degr>	Degr	IntP	IntP	Res	Res	Prod	40408	276296	552385	726814	581934	239147	67183
274.1	C11 H14 O8	20	δRAW	Degr>	Degr	IntP	IntP	Res	Res	Prod	42015	288588	581038	799177	623295	265655	71540
274.1	C11 H14 O8	30		Degr>	Degr	IntP	IntP	Res	Res	Prod	41090	330828	622287	822200	624060	262904	69684
274.1	C11 H14 O8	60		Degr>	Degr	IntP	IntP	Res	Res	Prod	44024	323582	691543	898450	685274	287283	72064
274.1	C11 H14 O8	120		Degr>	Degr	IntP	IntP	Res	Res	Prod	0	296479	684700	902028	689784	299020	91635
274.1	C11 H14 O8	180		Degr>	Degr	IntP	IntP	Res	Res	Prod	0	259586	543517	735403	638297	284662	85171
274.1	C11 H14 O8	300		Degr>	Degr	IntP	IntP	Res	Res	Prod	0	147773	415806	511974	412178	253877	78316
292.1	C11 H20 N2 O7	0	start RAW	IntP>	r.n.a.	IntP	IntP>	<IntP	r.n.a.	n.d.	12214	79629	68312	15098	0	0	0
292.1	C11 H20 N2 O7	10	end RAW	IntP>	r.n.a.	IntP	IntP>	<IntP	r.n.a.	n.d.	13597	66728	74320	15759	0	14407	0
292.1	C11 H20 N2 O7	20	δRAW	IntP>	r.n.a.	IntP	IntP>	<IntP	r.n.a.	n.d.	15853	82719	84092	17995	0	0	0
292.1	C11 H20 N2 O7	30		IntP>	r.n.a.	IntP	IntP>	<IntP	r.n.a.	n.d.	15128	101029	96957	19792	16859	17705	0
292.1	C11 H20 N2 O7	60		IntP>	r.n.a.	IntP	IntP>	<IntP	r.n.a.	n.d.	18032	0	124111	27788	21503	0	0
292.1	C11 H20 N2 O7	120		IntP>	r.n.a.	IntP	IntP>	<IntP	r.n.a.	n.d.	0	133405	153893	32256	21861	0	0
292.1	C11 H20 N2 O7	180		IntP>	r.n.a.	IntP	IntP>	<IntP	r.n.a.	n.d.	0	155239	124189	30294	19710	0	0
292.1	C11 H20 N2 O7	300		IntP>	r.n.a.	IntP	IntP>	<IntP	r.n.a.	n.d.	0	109614	93157	0	13157	0	0

As an example, the component $C_{11}H_{20}N_2O_7$ showed δRAW = 1.0065 (Screenshot 11, RT = 11.5 min, column H), assuming to be potentially a product. However, before it has been already identified as *IntP* >. By performing this assignment sequence, the δRAW value as calculated in Screenshot 11 was not relevant for reactivity class assignment in this case.

SI 5 Examples for reaction time courses

For better understanding of the reactivity classes, examples of RAW versus reaction time plots are provided. In each plot the DI data are provided in addition. The reactivity classes are explained from the LC data (at selected RT).

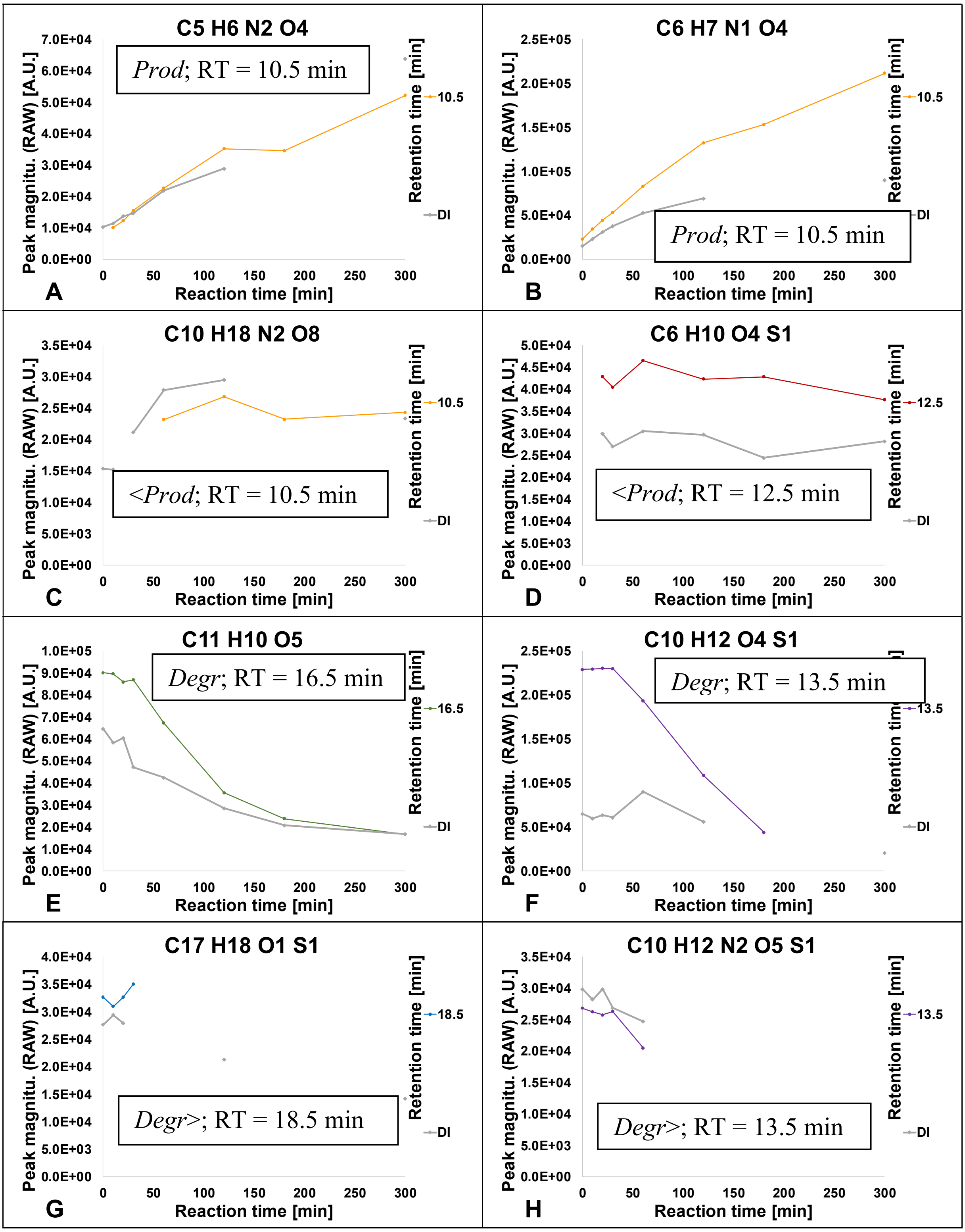

Fig. S4 **Examples for reaction time courses, *Prod*, *<Prod*, *Degr*, *Degr>***

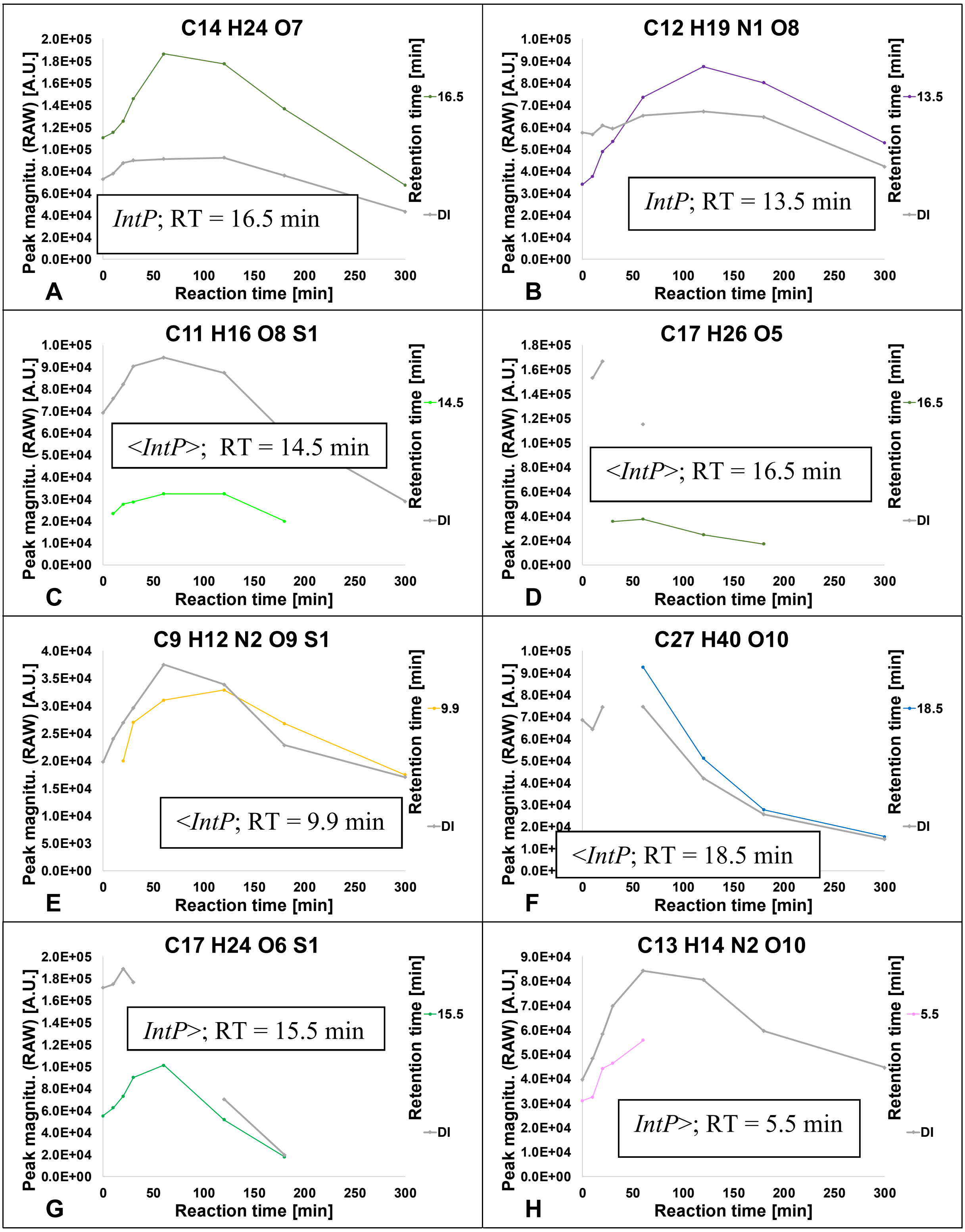

Fig. S5 **Examples for reaction time courses, *IntP*, < *IntP* >, < *IntP*, *IntP* >**

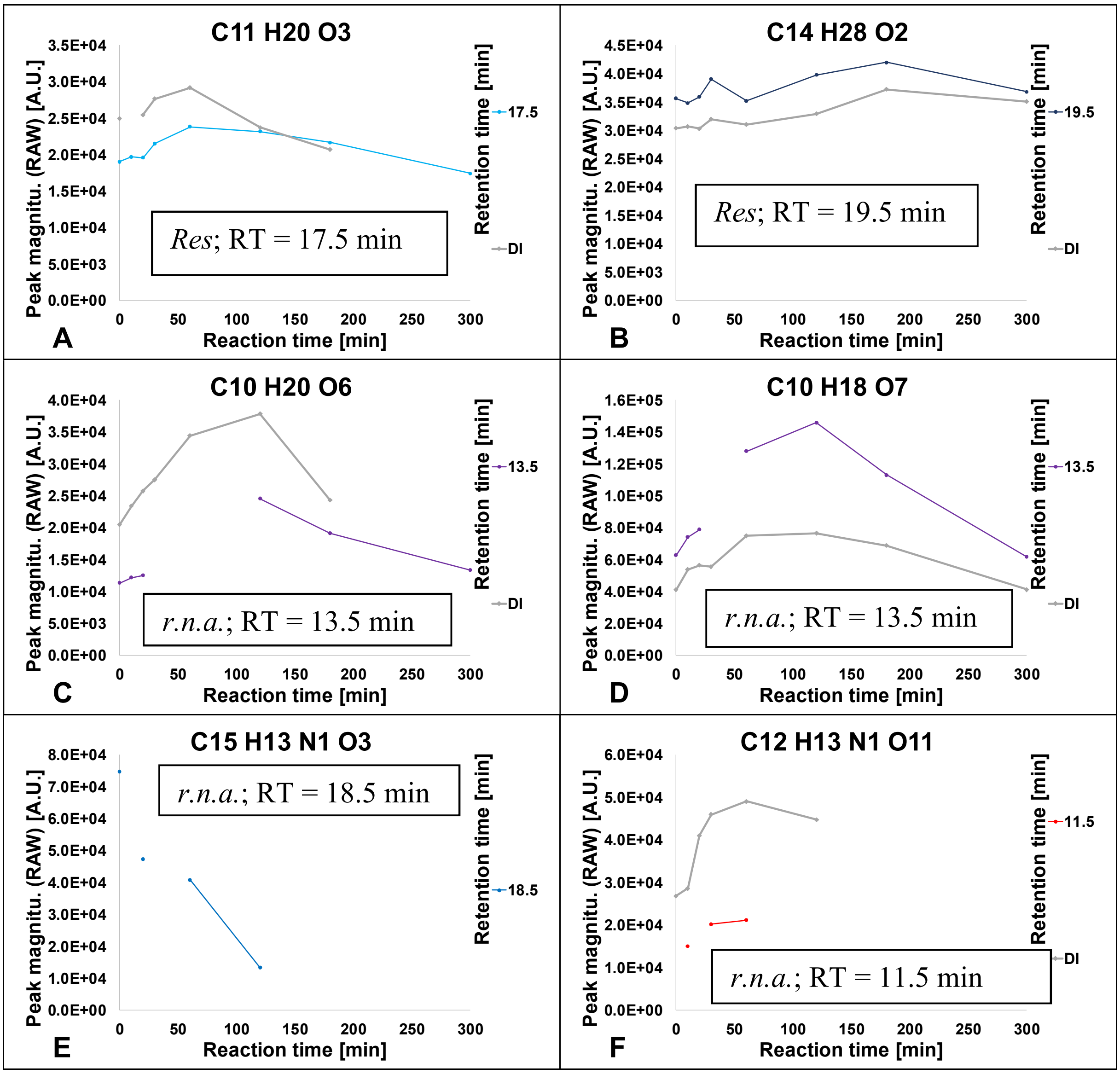

Fig. S6 Examples for reaction time courses, *Res*, *r.n.a.*

Screenshot 13 RAW values for replicates A and B of $C_{10}H_{18}O_7$ (Fig. S6 D)

	RT = 13.5 min	A,B average	A	B	relative
t (min)	formula	peak magnitude	peak magnitude	peak magnitude	difference A;B
0	C10 H18 O7	62679.45	60569.6	64789.3	6.73
10	C10 H18 O7	74003	82852.7	65153.3	23.92
20	C10 H18 O7	78720.2	83369.8	74070.6	11.81
30	C10 H18 O7	exclude	96098	70501.9	30.73
60	C10 H18 O7	127889.15	133862.3	121916	9.34
120	C10 H18 O7	145815.85	142568	149063.7	4.45
180	C10 H18 O7	112949.65	111363.1	114536.2	2.81
300	C10 H18 O7	61605.45	62199	61011.9	1.93

The reactivity class *r.n.a.* was assigned here because the RAW value at 30 min was excluded because of too large relative A,B peak magnitude difference.

The reactivity class *r.n.a.* was assigned to $C_{12}H_{13}N_1O_{11}$ (Fig. S6 F) at RT = 11.5 min because both A and B replicates showed data gaps at different reaction times. Here low abundance seems be the reason for release of data gaps.

SI 6 Reactivity class balances

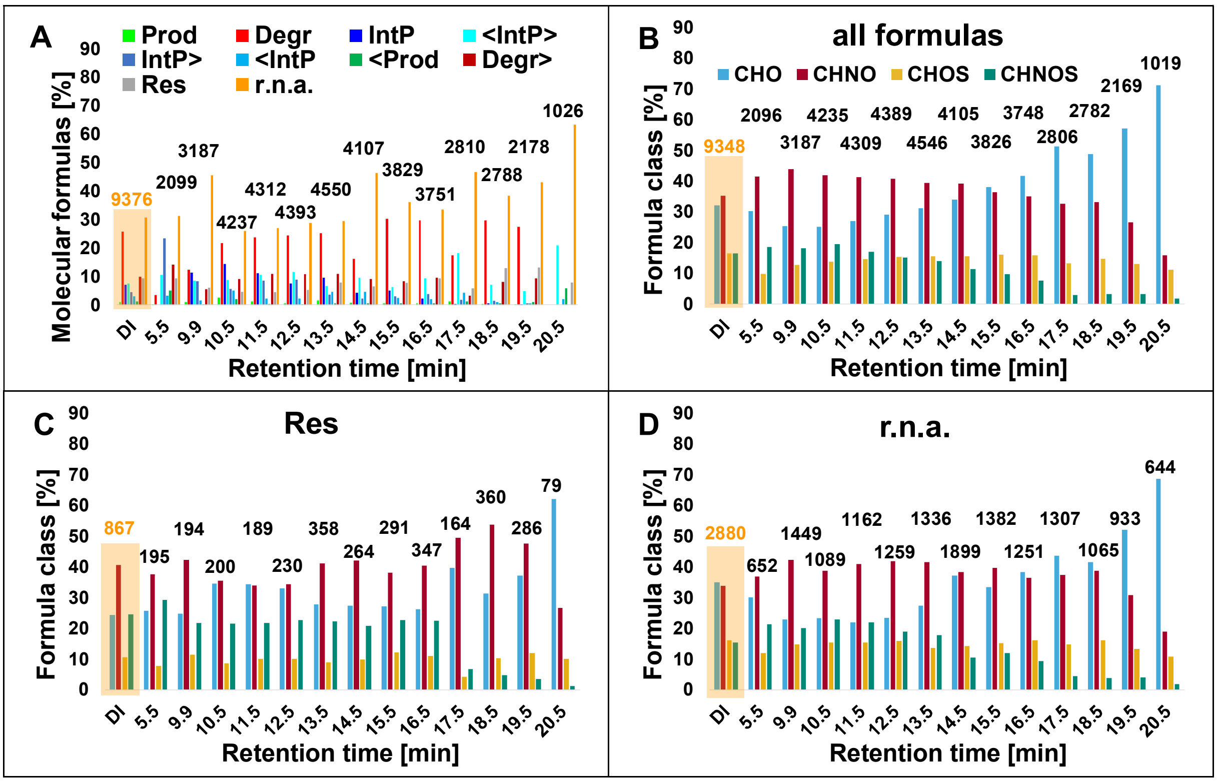

Fig. S7 Distribution of reactivity classes as function of retention time (RT), A: broken down to all reactivity classes (except for *n.d.*), B: total part of component classes; C: resistant MF normalized to the sum of components; D: reactivity not assigned MF

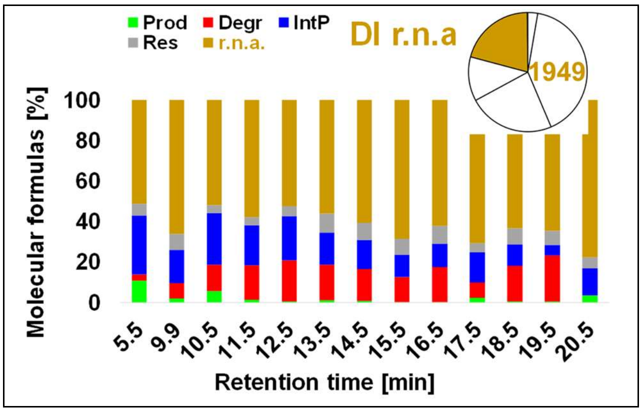

Fig. S8 Comparison of reactivity classes distribution, DI 8TP model versus LC model. Distribution of not assigned MF (*r.n.a.*)

The errors of counting MFs in specific reactivity classes can be calculated empirically by using the two replicate sample peak magnitude (RAW) values (from A and B) in separate and comparing them to the data calculated from the average RAW. Only RAW were used for error calculation after exclusion of A and B RAW with A-B differences larger than 26.5 % (95 % percentile). This means that for r.n.a. no errors are calculated because this is a function of data gap exclusion and would strongly depend on the 95 % percentile threshold.

Only the errors for the reactivity classes Prod (and <*Prod*), *Degr* (and *Degr*>), *IntP* (and <*IntP*>, <*IntP*, *IntP*>), Res were calculated. The number of each reactivity class is the result of a complex data evaluation and hence the estimation is only possible on an empirical level.

The error was calculated: [Max (A, B, average sample) - Min (A, B, average sample)] / Median (A, B, average sample) in %.

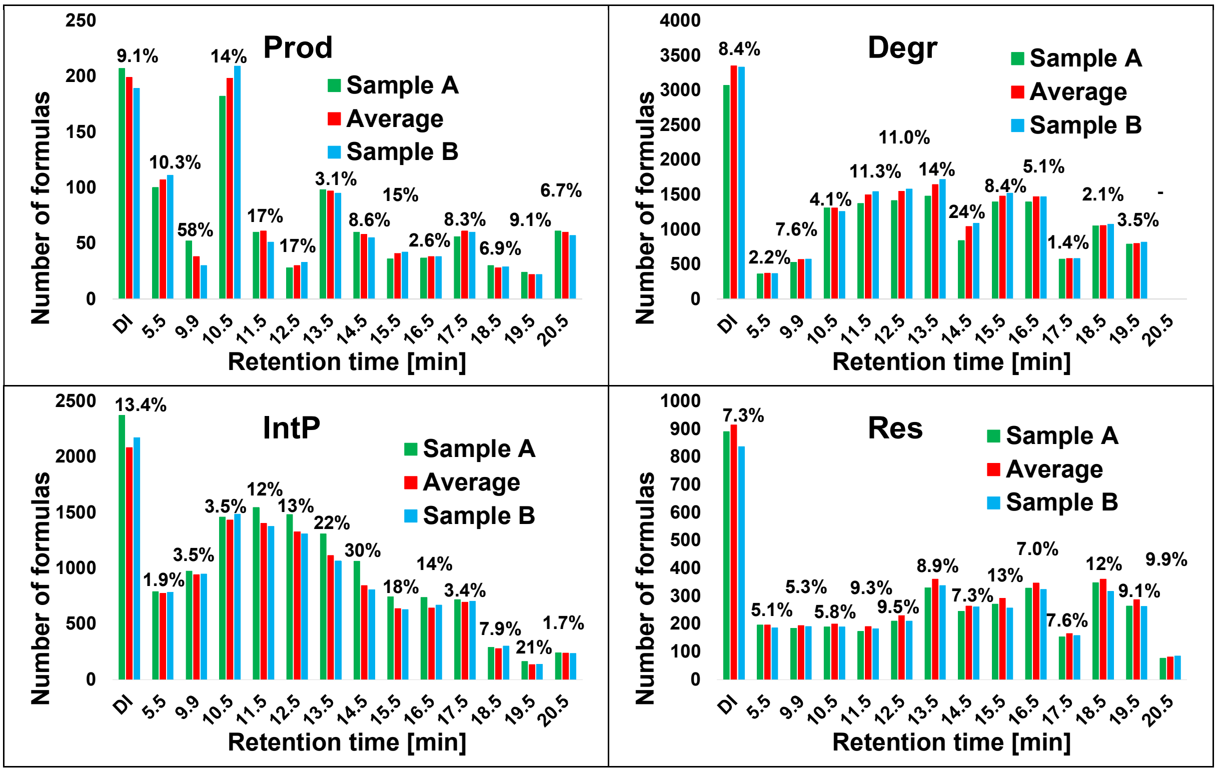

Fig. S9 **Calculated errors for counting the reactivity classes *Prod, Degr, IntP, Res.* Samples A and B and the evaluation of A-B average RAW values are compared**

SI7 Chemical distribution of DOM reactivity classes in van Krevelen diagrams

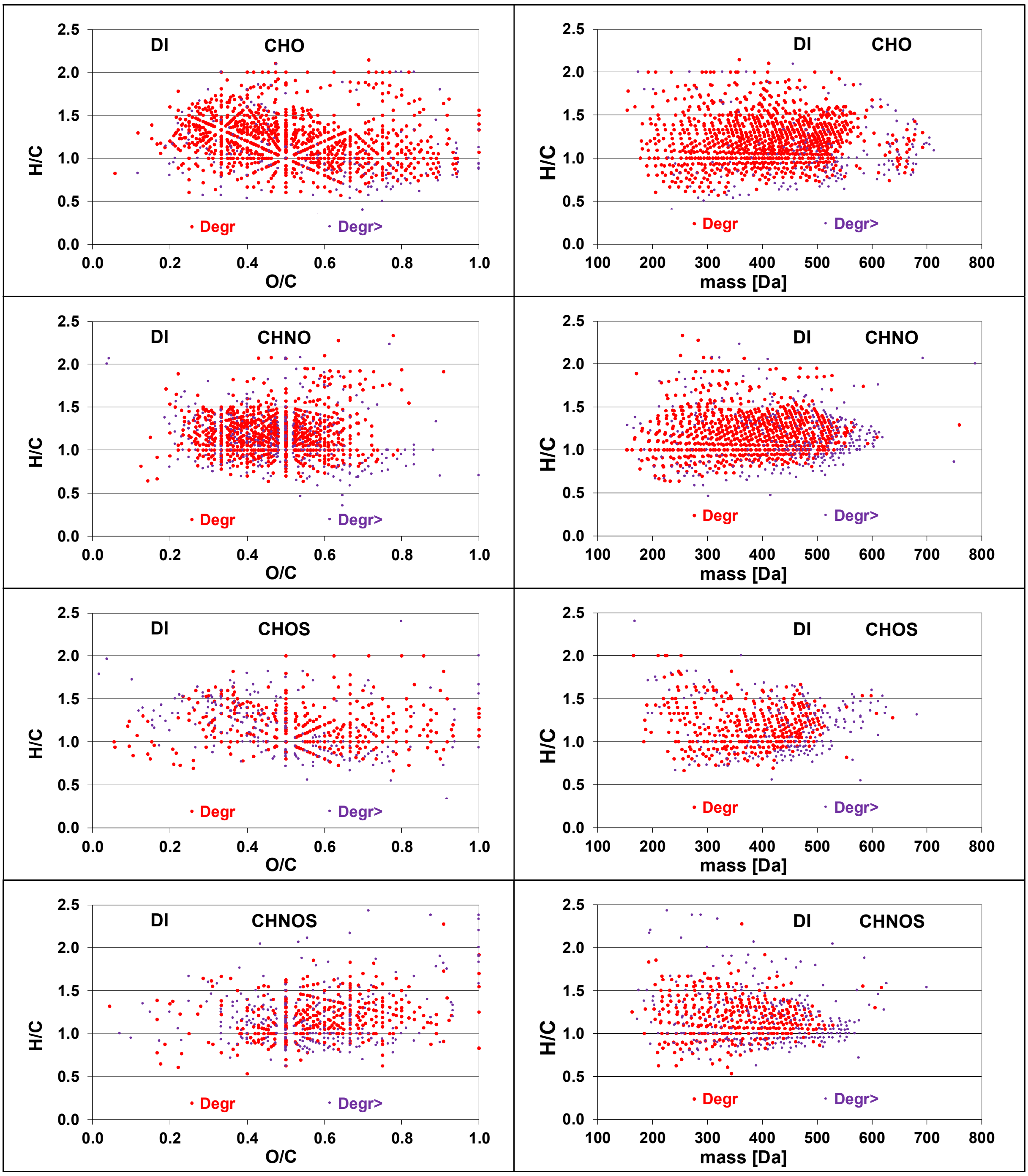

Fig. S10 Reactivity of MF, DI model, *Degr* and *Degr>* in vK diagrams

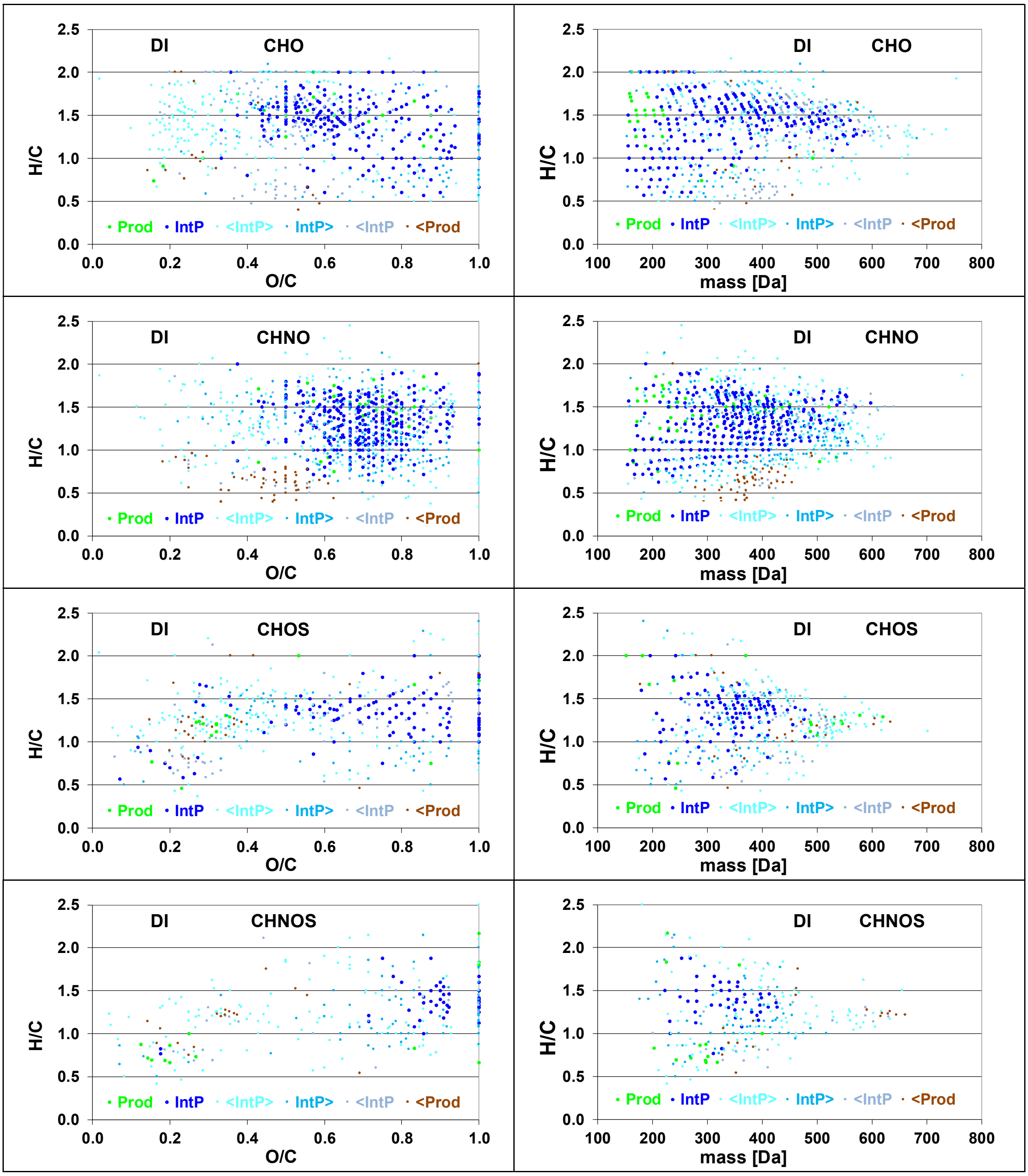

Fig. S11 **Reactivity of MF, DI model, *Prod*, *<Prod*, *IntP*, *<IntP>*, *<IntP*, *IntP>* in vK diagrams**

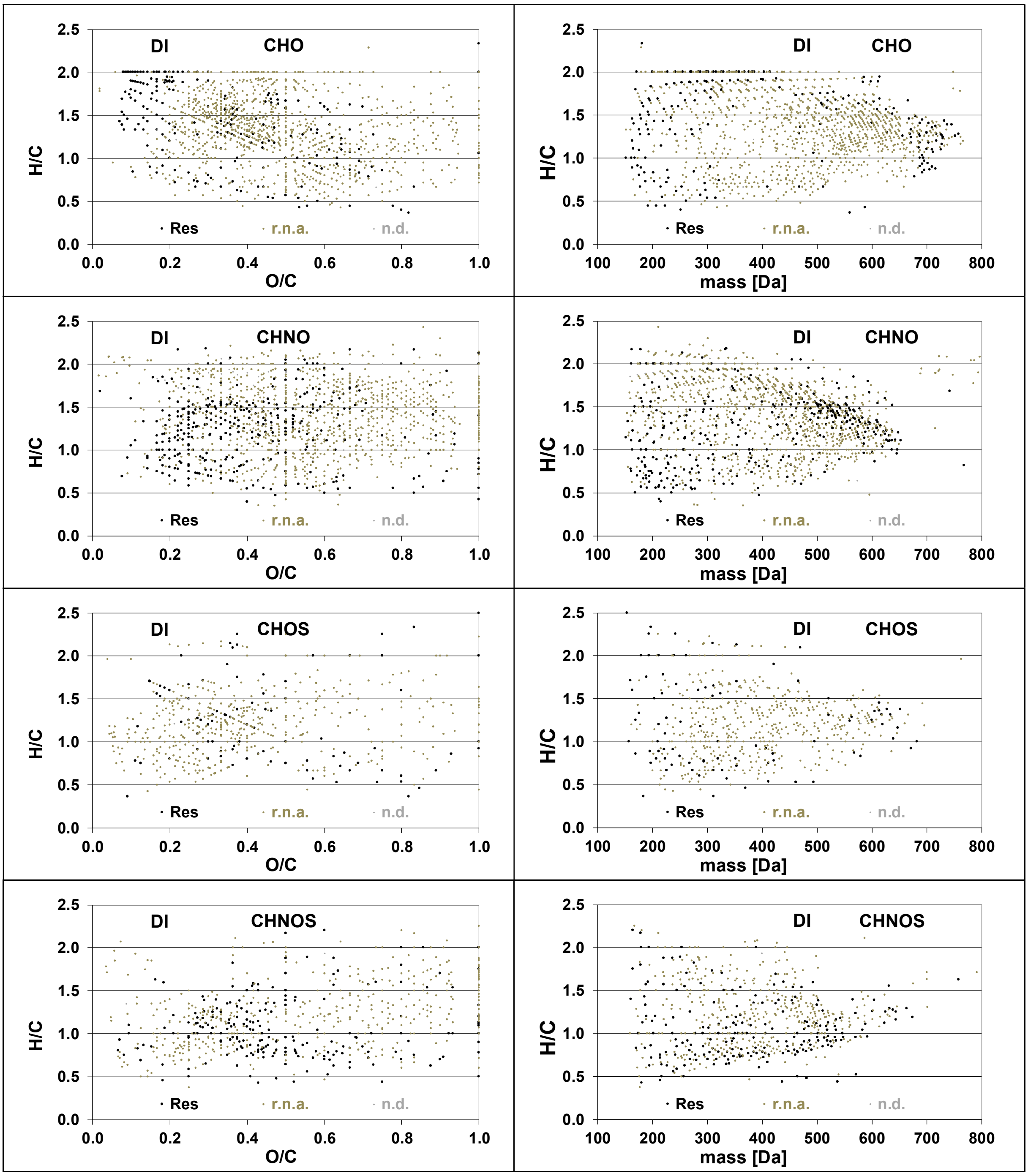

Fig. S12 Reactivity of MF, DI model, *Res*, *r.n.a.*, *n.d.* in vK diagrams

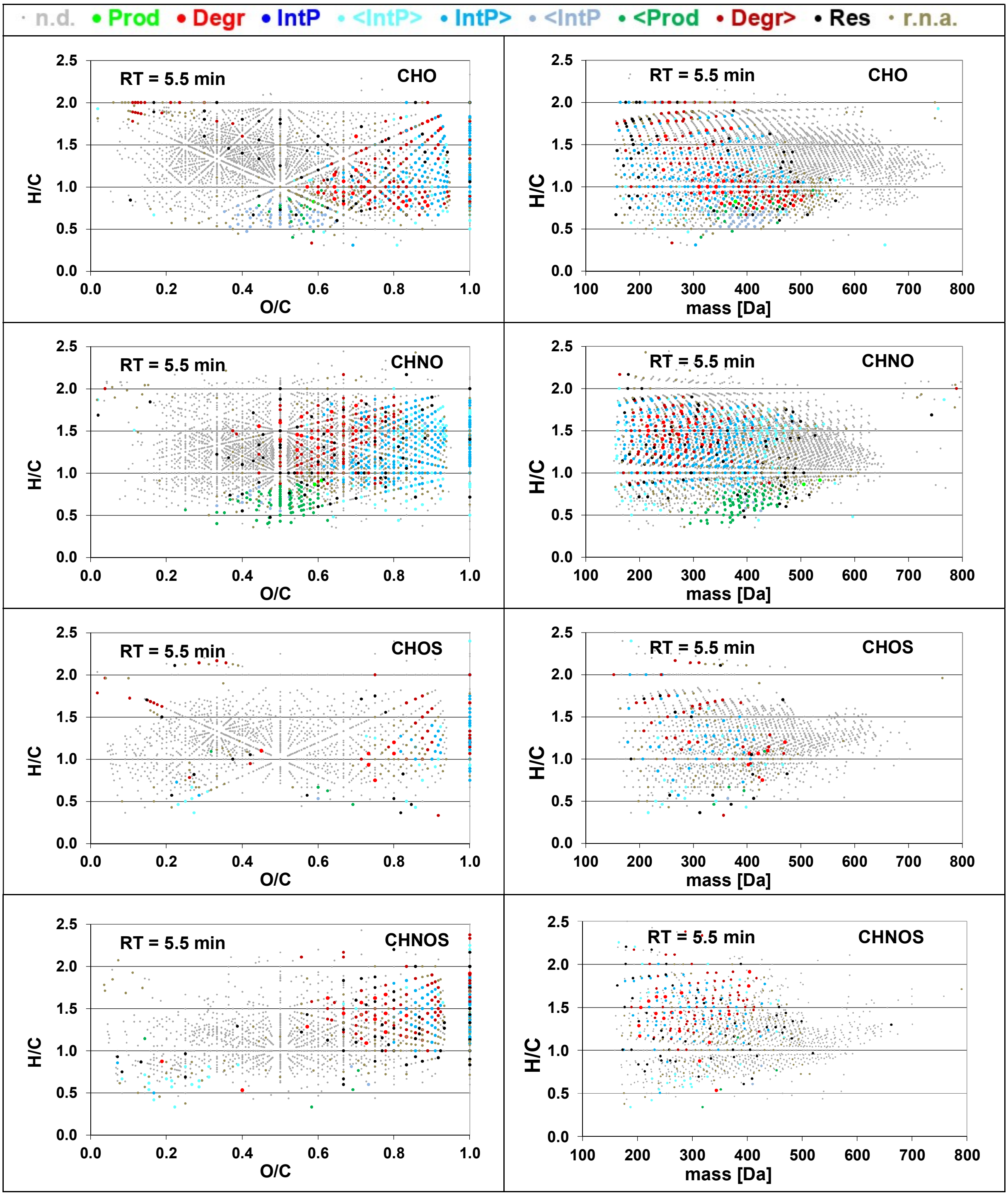

Fig. S13 Reactivity classes of MF, LC model in vK diagrams, RT = 5.5 min

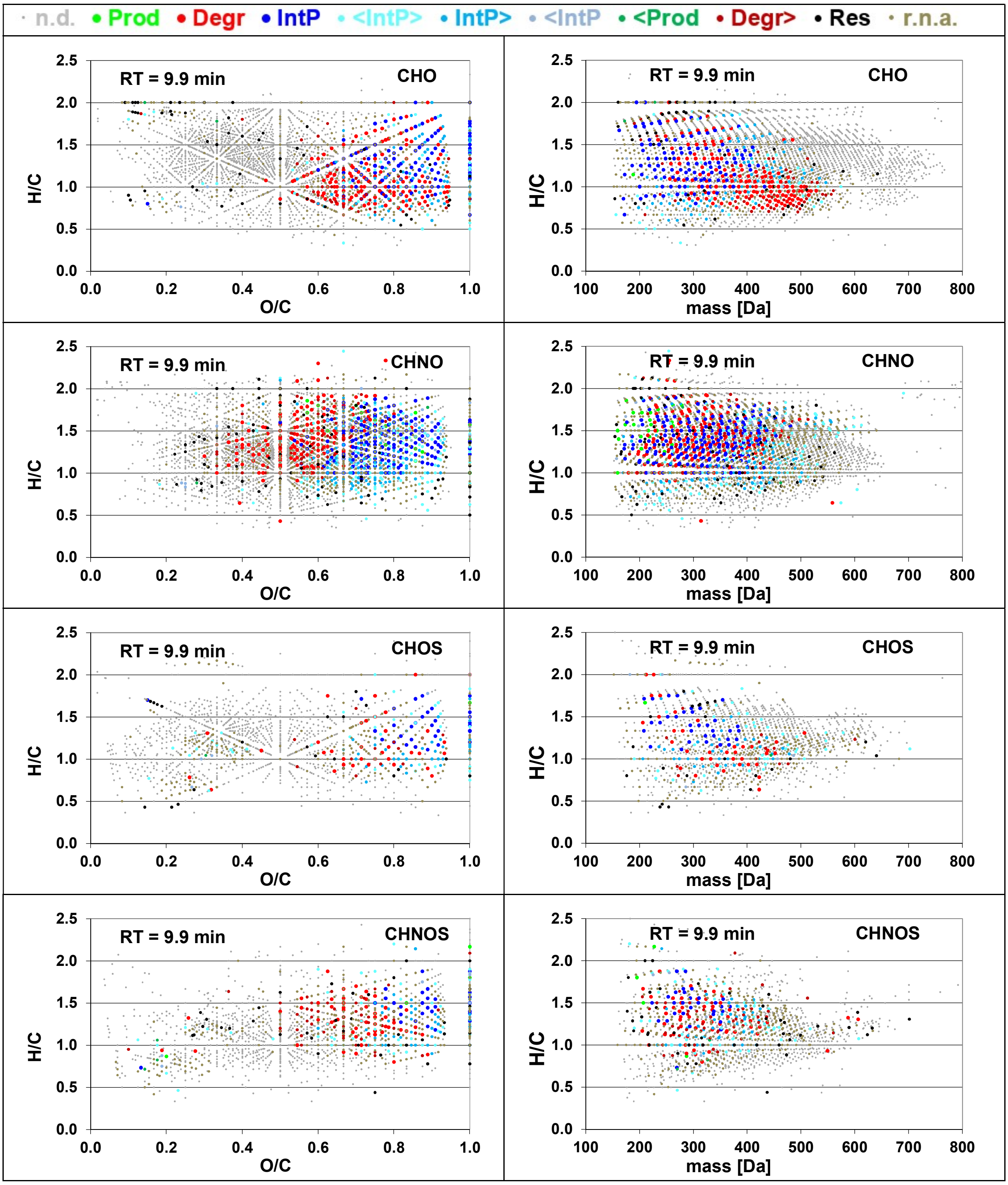

Fig. S14 Reactivity classes of MF, LC model in vK diagrams, RT = 9.9 min

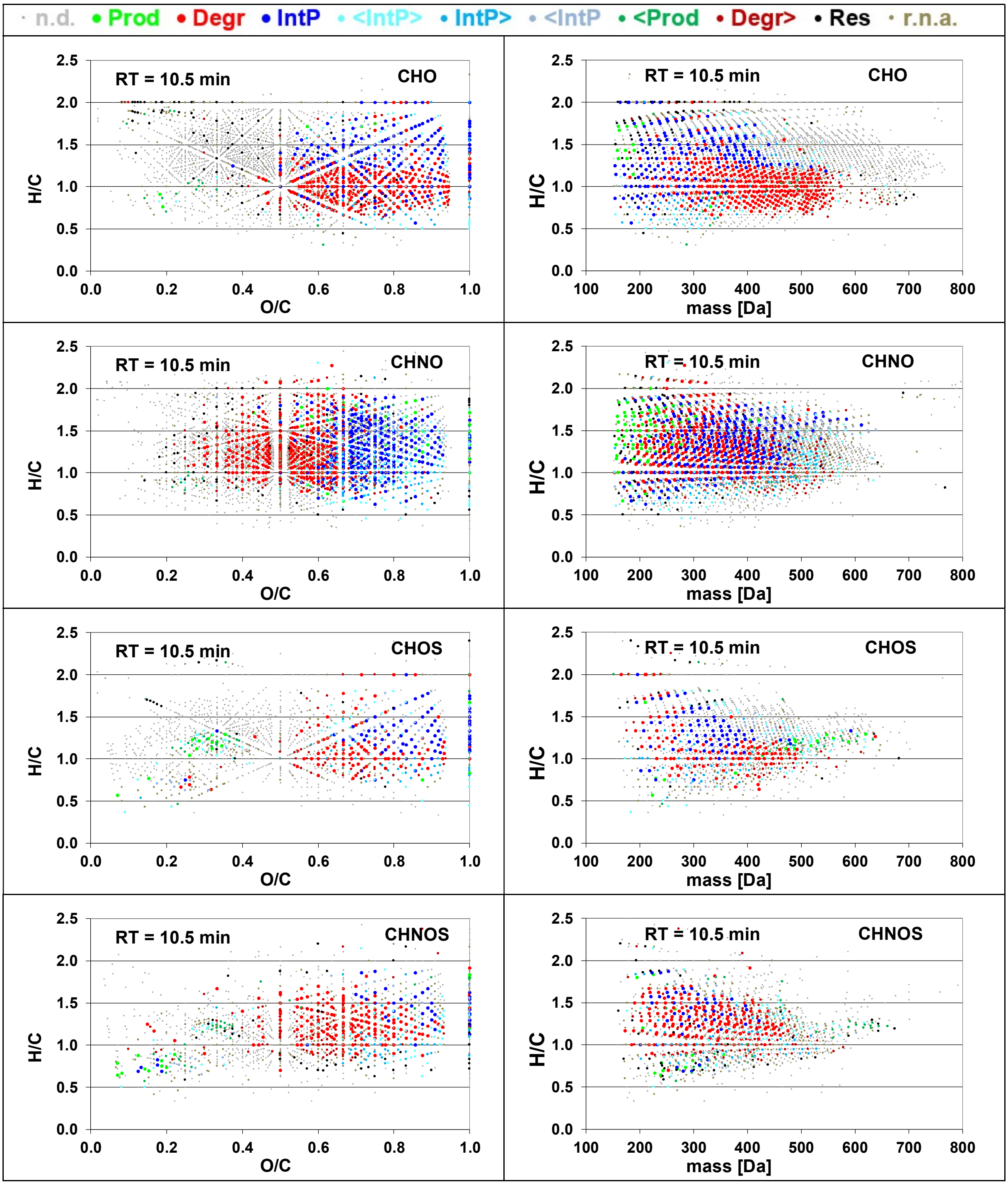

Fig. S15 Reactivity classes of MF, LC model in vK diagrams, RT = 10.5 min

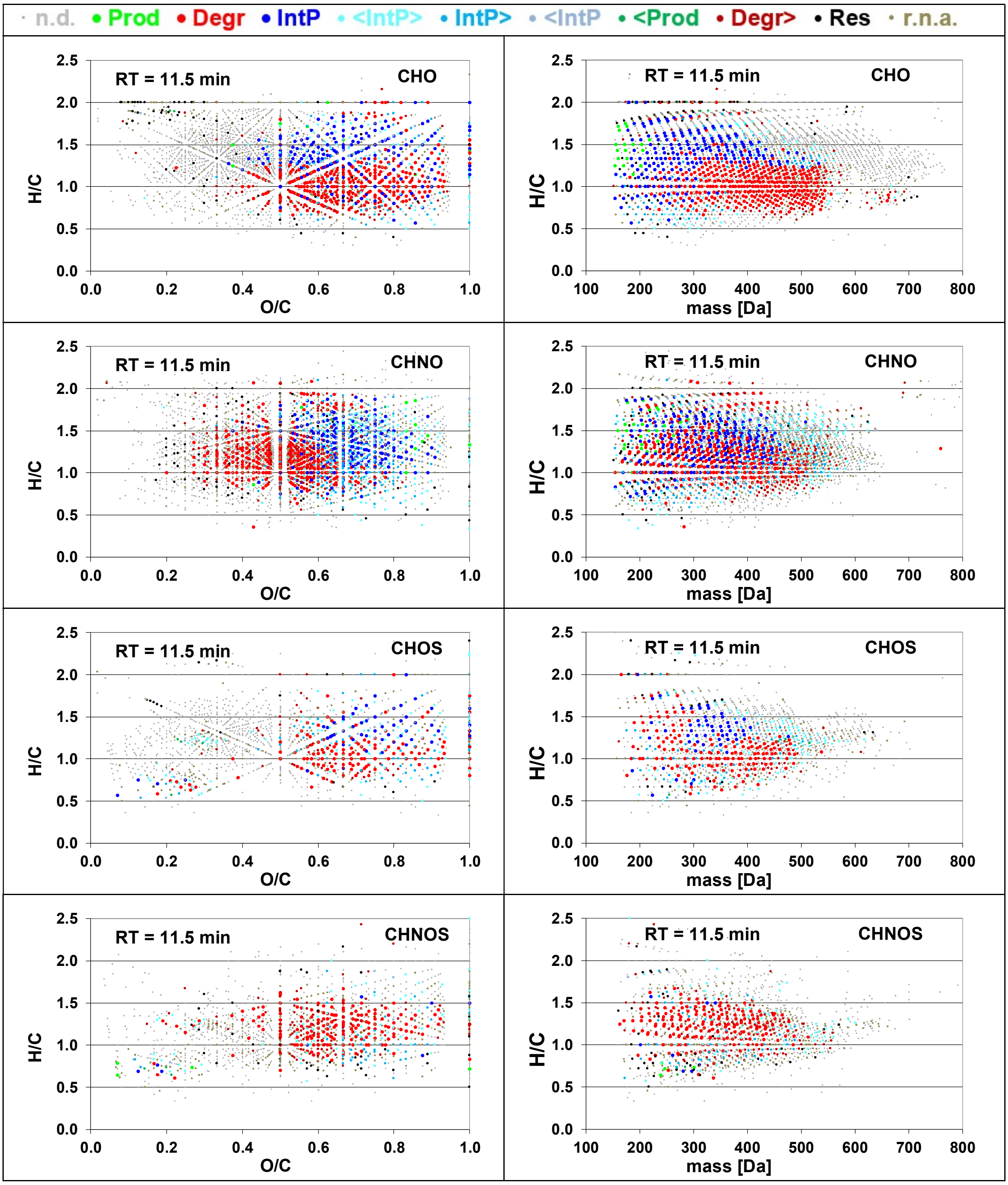

Fig. S16 Reactivity classes of MF, LC model in vK diagrams, RT = 11.5 min

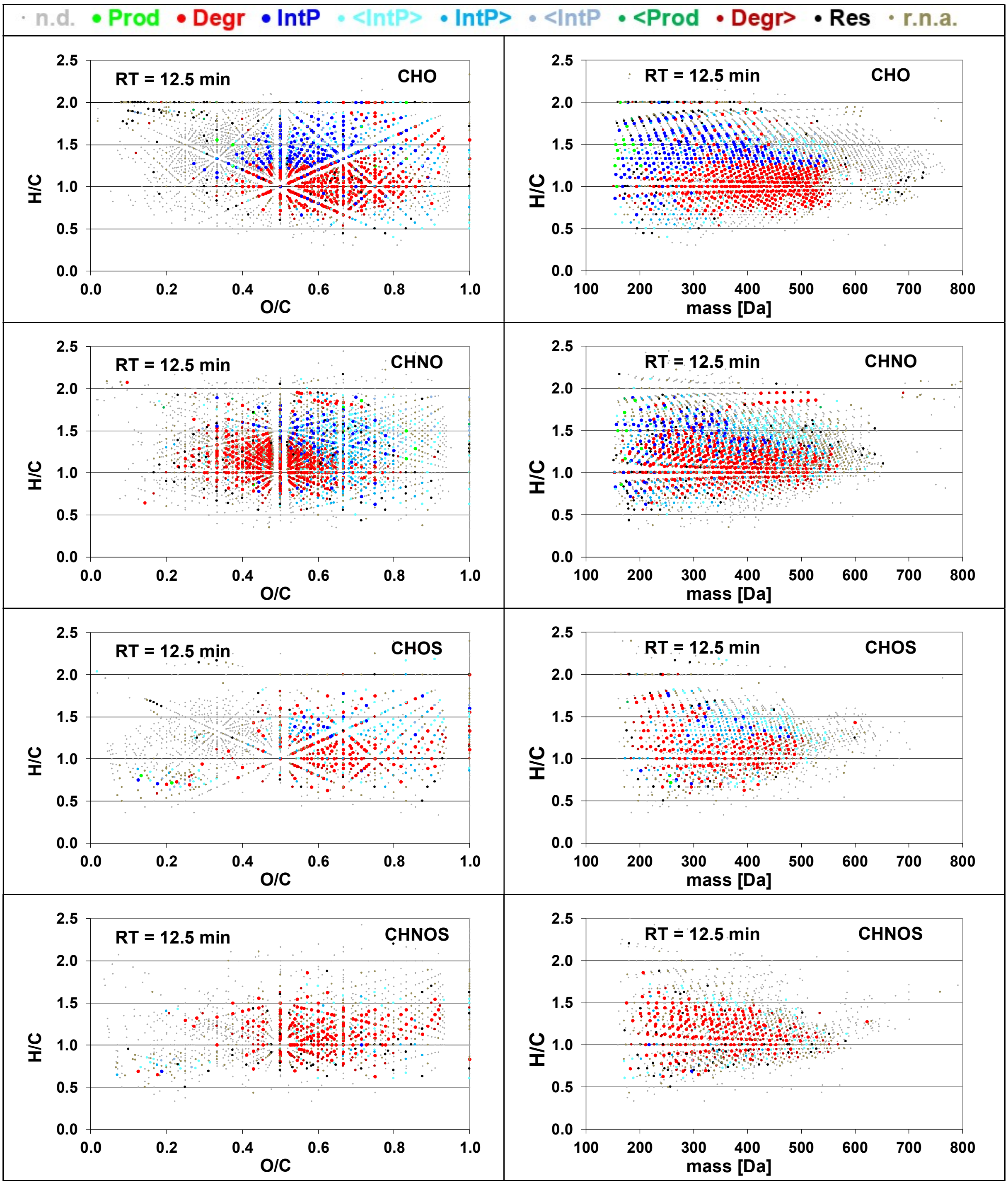

Fig. S17 Reactivity classes of MF, LC model in vK diagrams, RT = 12.5 min

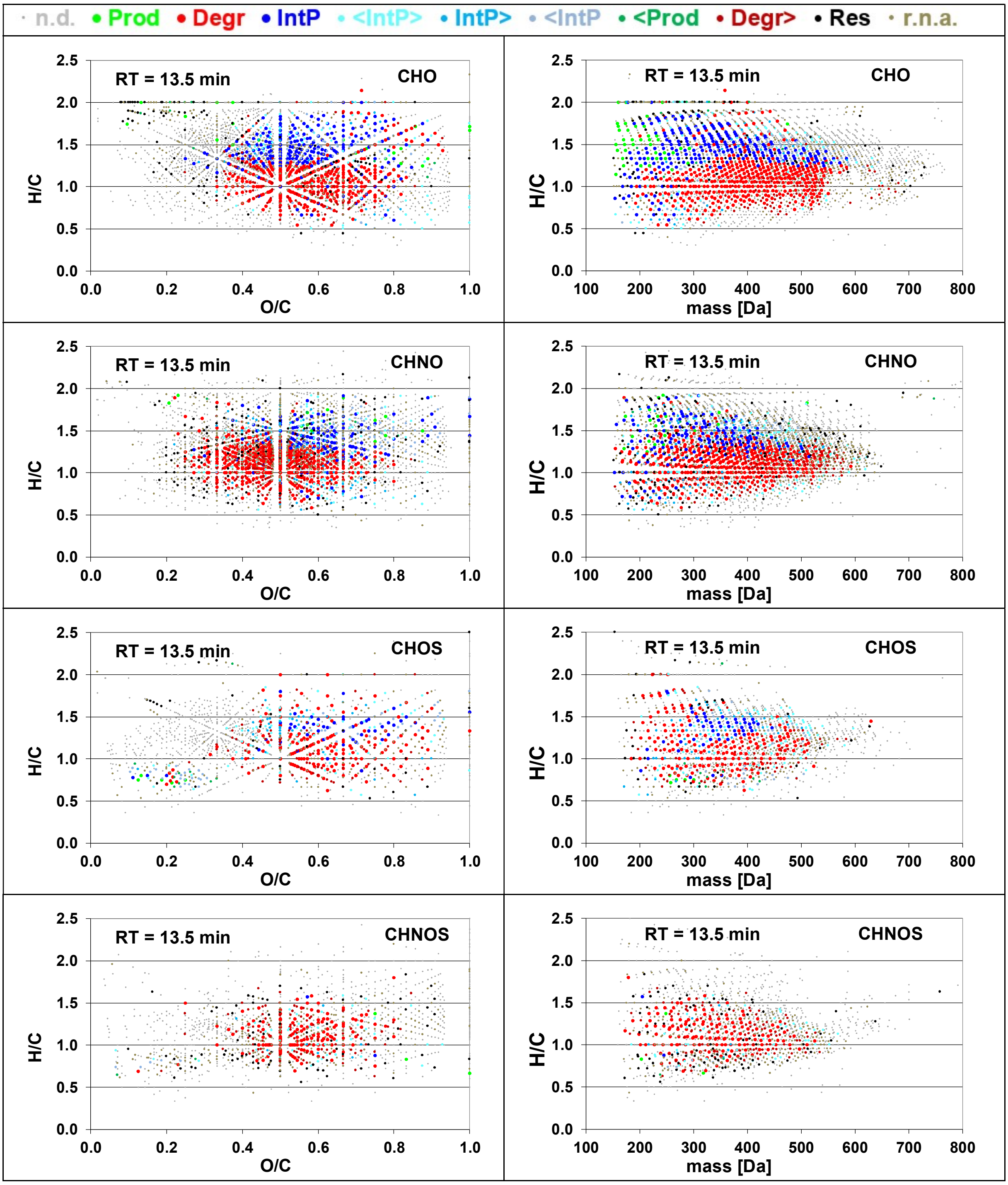

Fig. S18 Reactivity classes of MF, LC model in vK diagrams, RT = 13.5 min

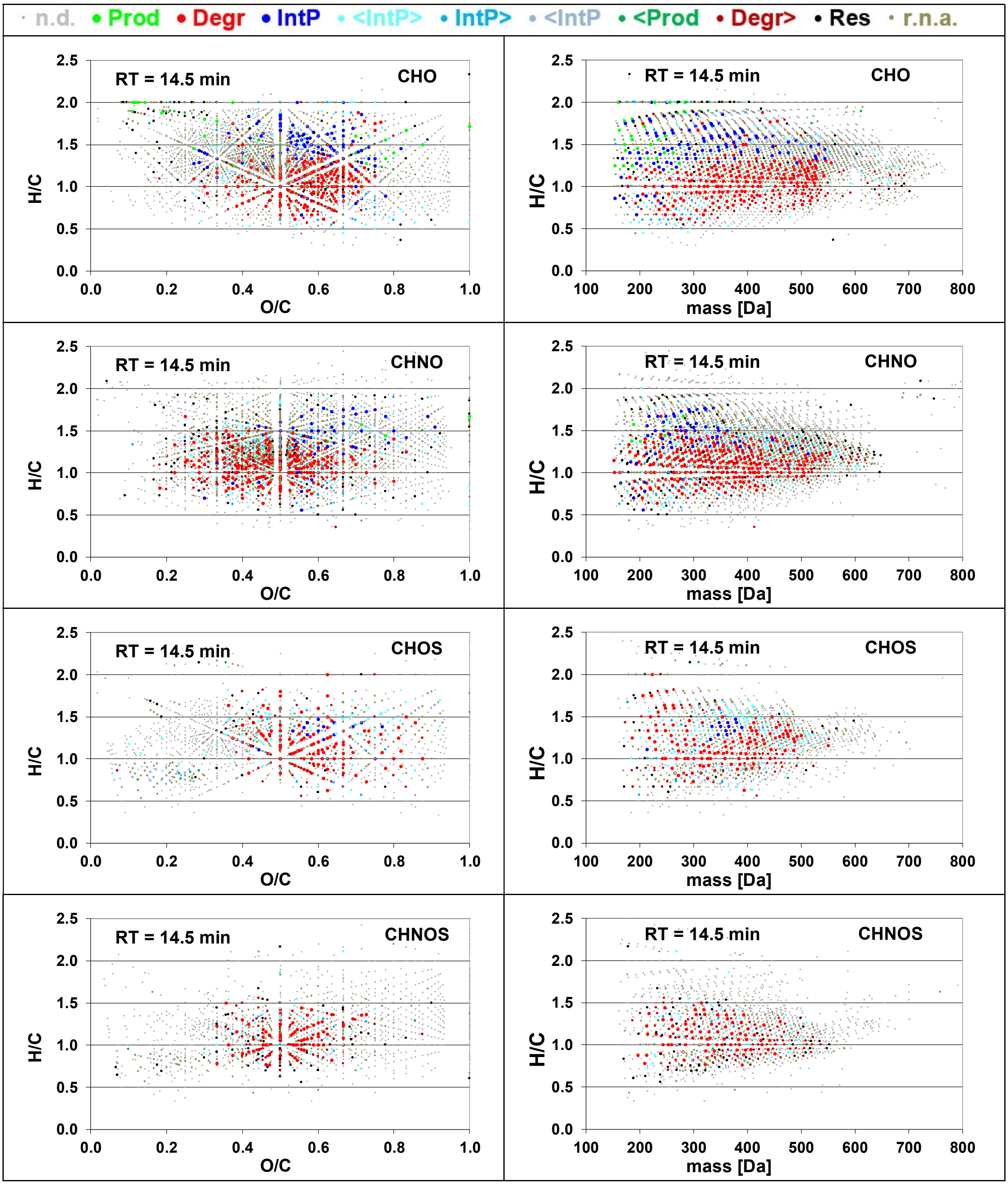

Fig. S19 Reactivity classes of MF, LC model in vK diagrams, RT = 14.5 min

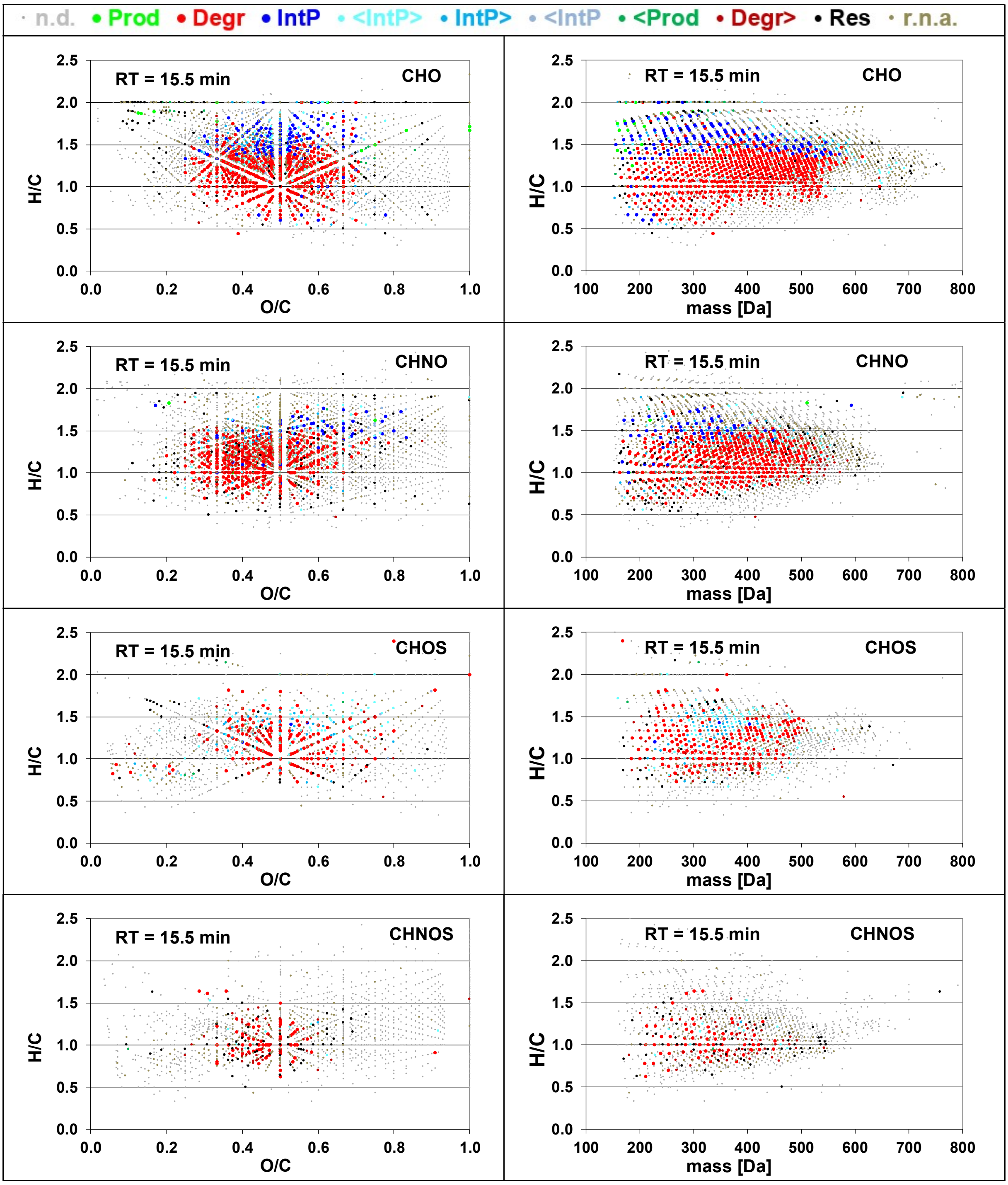

Fig. S20 Reactivity classes of MF, LC model in vK diagrams, RT = 15.5 min

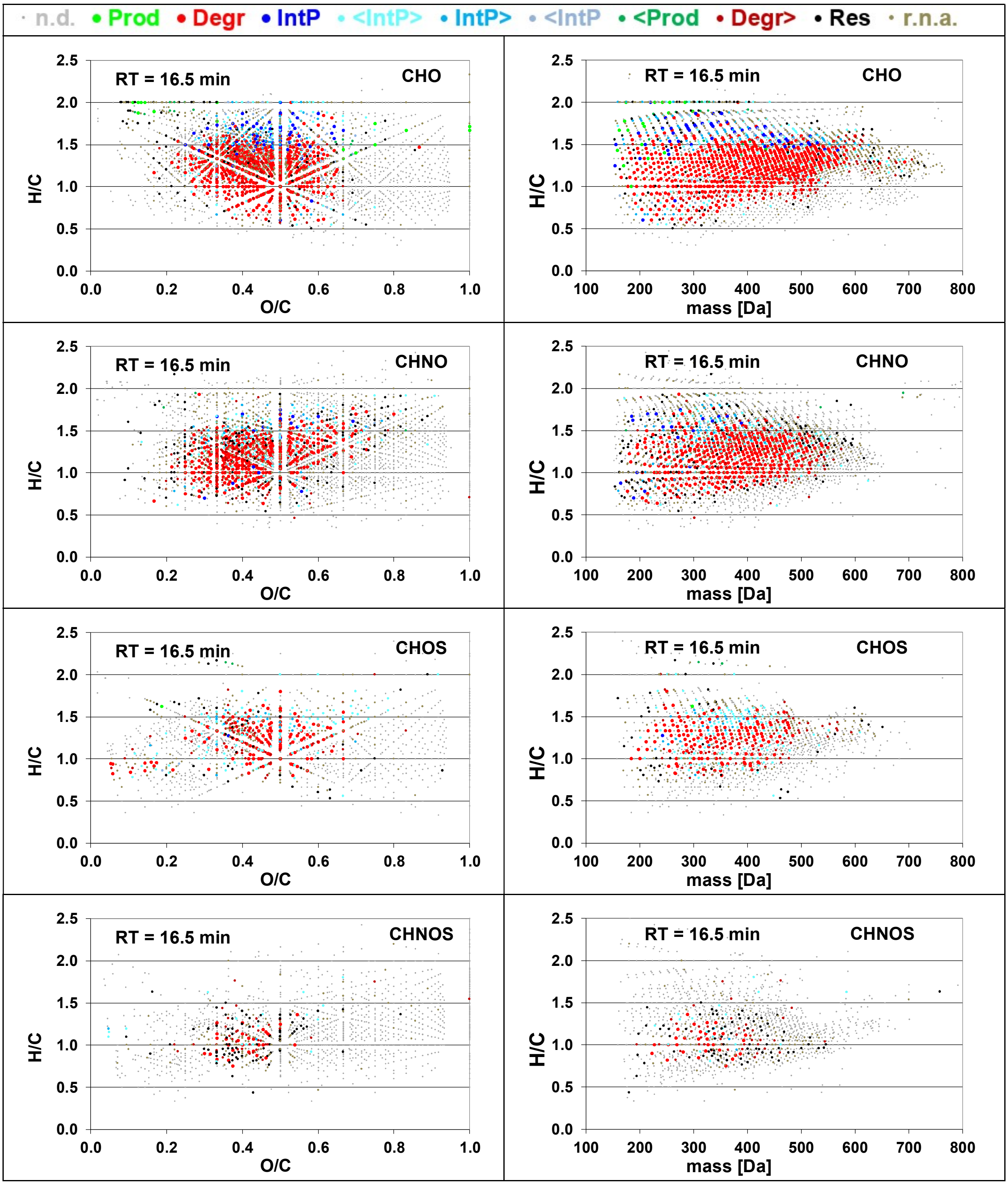

Fig. S21 Reactivity classes of MF, LC model in vK diagrams, RT = 16.5 min

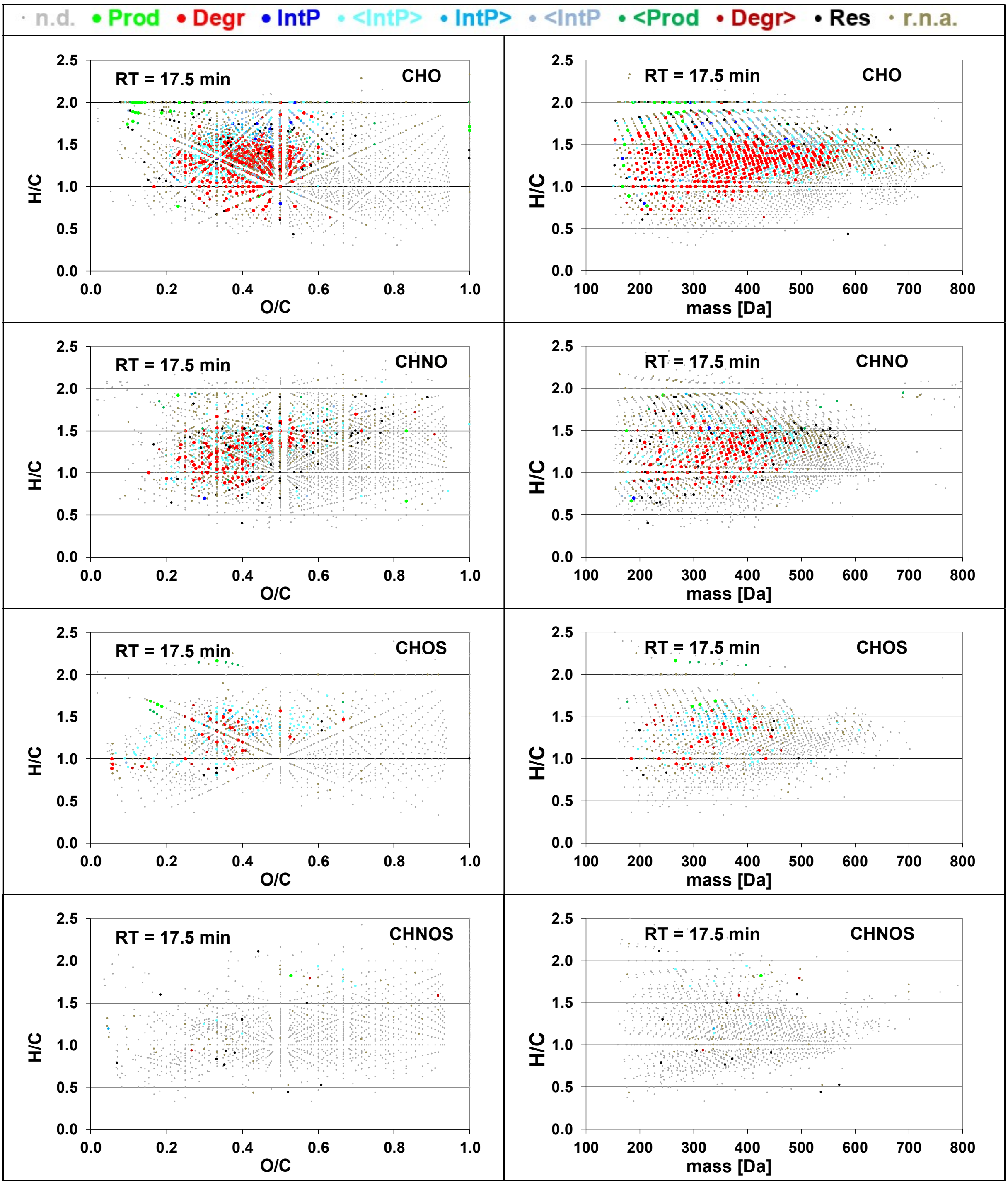

Fig. S22 Reactivity classes of MF, LC model in vK diagrams, RT = 17.5 min

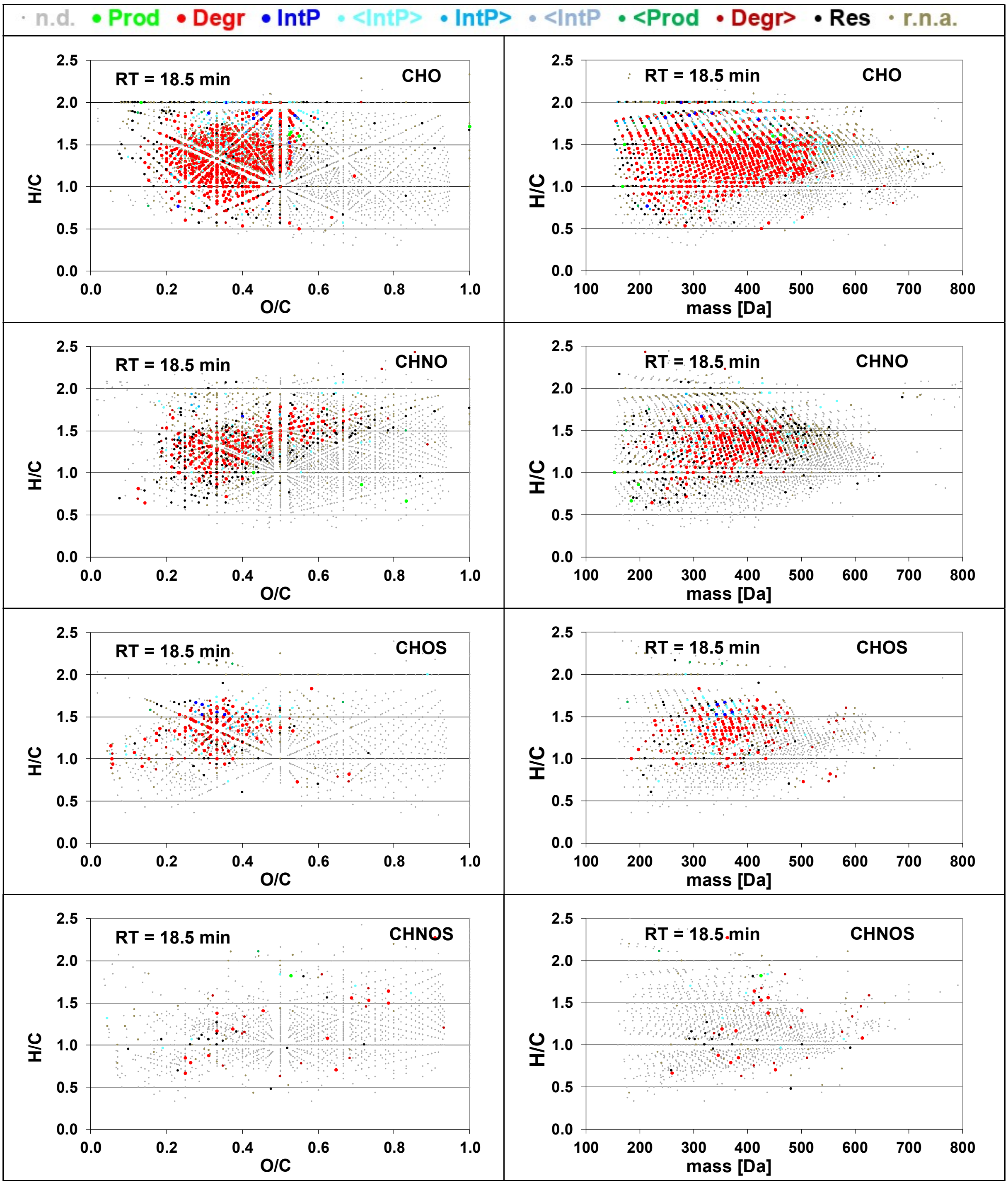

Fig. S23 Reactivity classes of MF, LC model in vK diagrams, RT = 18.5 min

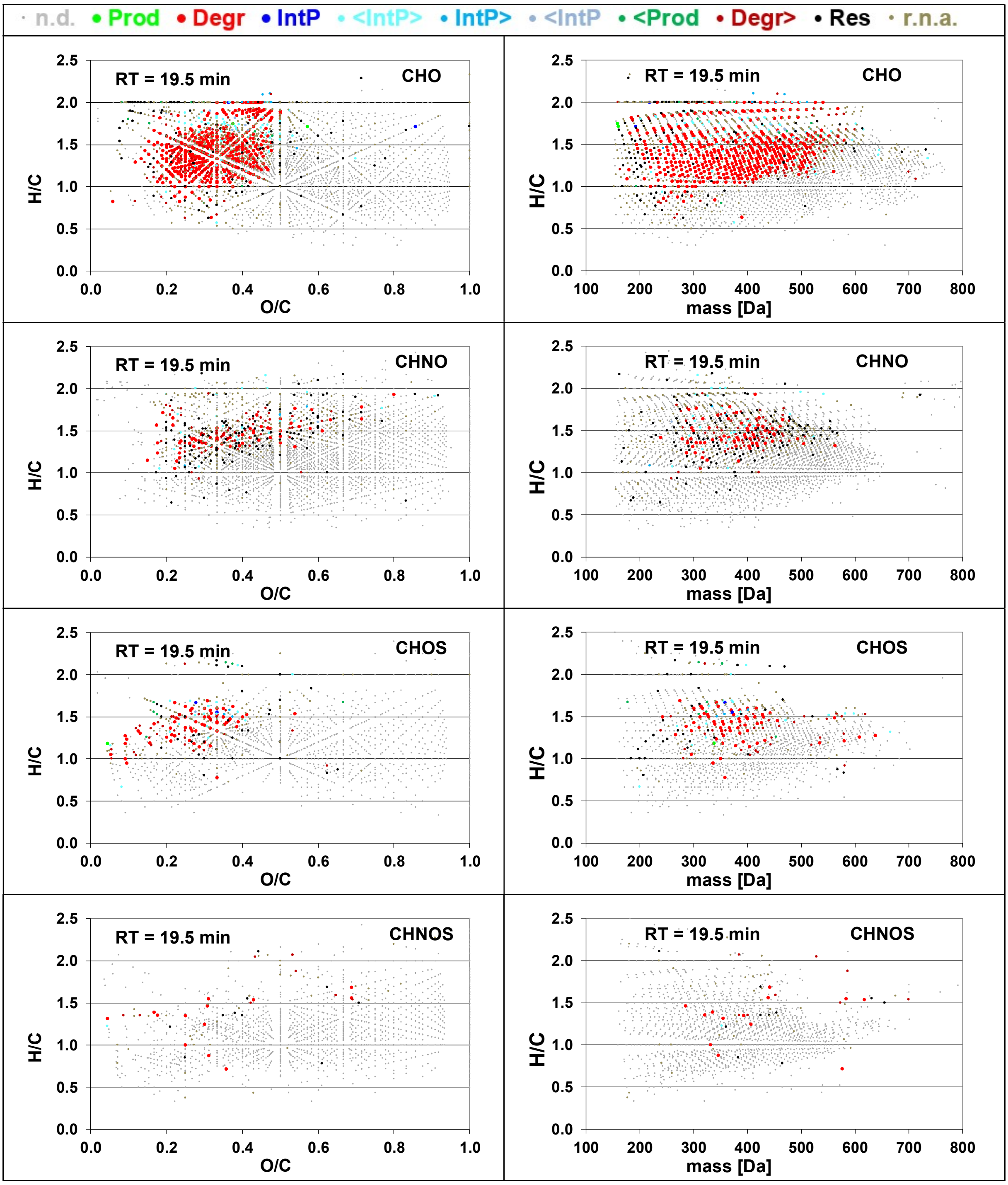

Fig. S24 Reactivity classes of MF, LC model in vK diagrams, RT = 19.5 min

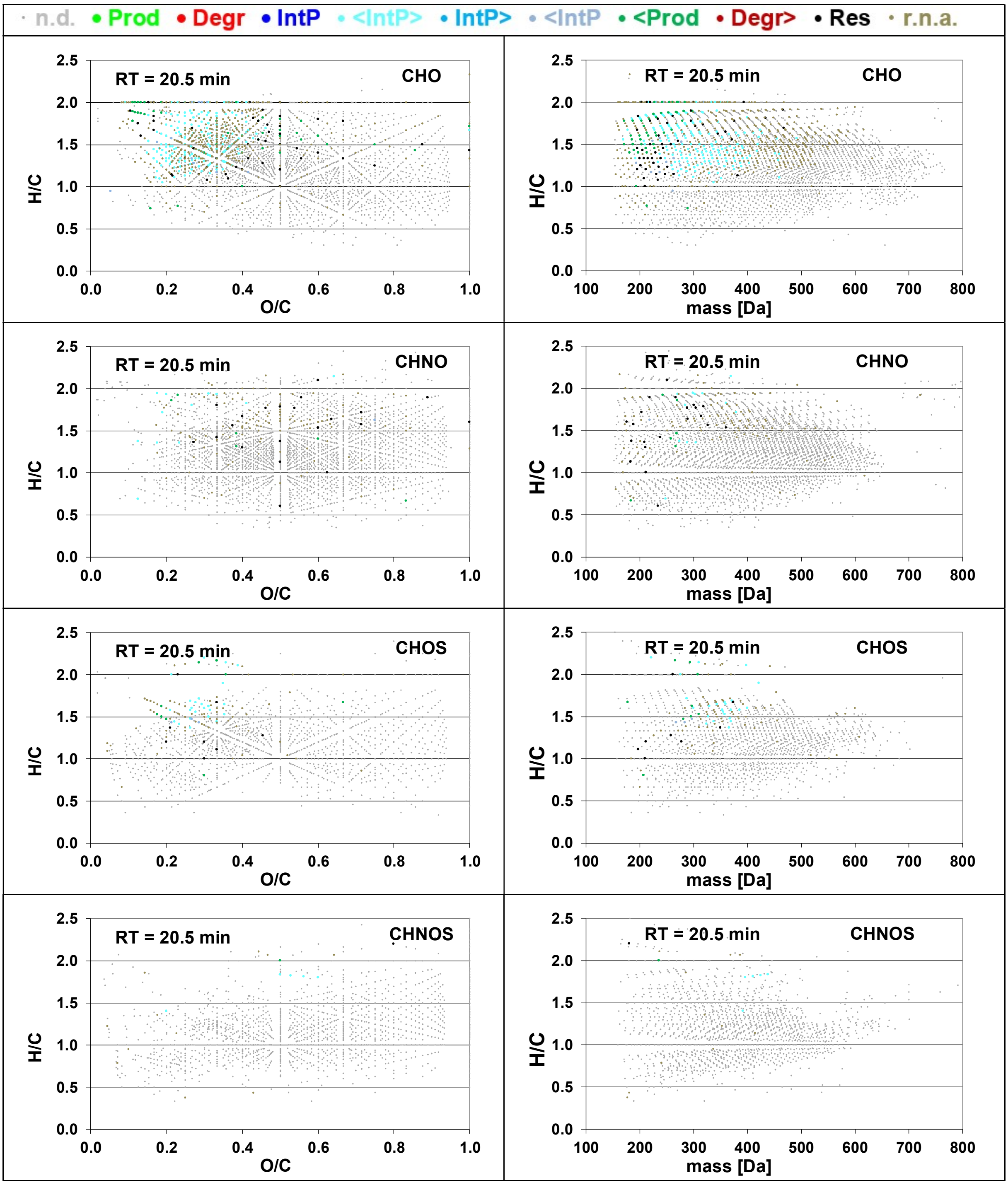

Fig. S25 Reactivity classes of MF, LC model in vK diagrams, RT = 20.5 min

SI 8 Maximum RAW for *IntP* as function of reaction time (TP)

Screenshot 13 Relation matrix between *IntP* maximum time point and different molecular descriptors

A	B	C	D	E	F	G	H	I	J	K	L	M	N	O
H/C	DI	5.51	9.86	10.5	11.5	12.5	13.5	14.5	15.5	16.5	17.5	18.5	19.5	20.5
CHO	0.1	-0.4	0.5	0.7	0.7	0.7	0.6	0.6	0.5	0.4	0.4	0.2	0.3	0.3
CHNO	0.3	-0.2	0.5	0.7	0.7	0.6	0.7	0.6	0.5	0.4	0.4	0.3	0.2	0.2
CHOS	0.0	0.1	0.4	0.5	0.3	0.2	0.0	-0.1	-0.2	0.1	0.1	0.4	0.1	-0.2
CHNOS	0.1	-0.2	0.2	0.3	0.2	-0.4	-0.3	-0.2	0.3	0.2	0.1	0.7	#####	0.6
Aimod	DI	5.5	9.9	10.5	11.5	12.5	13.5	14.5	15.5	16.5	17.5	18.5	19.5	20.5
CHO	-0.1	0.5	-0.2	-0.6	-0.7	-0.6	-0.6	-0.6	-0.5	-0.5	-0.5	-0.3	-0.4	-0.3
CHNO	-0.3	0.3	-0.4	-0.6	-0.6	-0.6	-0.7	-0.6	-0.5	-0.4	-0.3	-0.3	-0.2	-0.4
CHOS	0.1	0.1	0.0	-0.2	0.0	0.0	0.1	0.1	0.3	-0.1	-0.1	-0.2	0.1	0.2
CHNOS	0.1	0.2	0.2	0.2	0.2	0.5	0.5	0.2	-0.5	0.1	-0.1	-0.5	#####	-0.7
O/C	DI	5.5	9.9	10.5	11.5	12.5	13.5	14.5	15.5	16.5	17.5	18.5	19.5	20.5
CHO	0.1	-0.7	-0.3	-0.2	-0.2	-0.4	-0.2	0.0	0.2	0.2	0.4	0.1	0.3	0.3
CHNO	0.1	-0.4	0.1	0.2	0.2	0.2	0.3	0.6	0.4	0.2	0.3	-0.2	-0.1	0.5
CHOS	-0.1	-0.1	-0.2	-0.2	-0.3	-0.1	-0.1	-0.1	-0.4	-0.1	0.0	0.4	-0.1	0.0
CHNOS	-0.2	0.0	0.0	-0.3	-0.3	-0.5	-0.7	-0.2	0.4	-0.5	0.0	0.1	#####	0.8
mass	DI	5.5	9.9	10.5	11.5	12.5	13.5	14.5	15.5	16.5	17.5	18.5	19.5	20.5
CHO	-0.1	0.2	-0.3	-0.1	0.0	0.0	0.2	0.1	-0.2	-0.1	-0.4	0.0	-0.2	-0.2
CHNO	-0.2	0.1	-0.3	-0.1	-0.2	-0.1	0.0	-0.2	0.0	-0.3	-0.2	0.0	-0.3	-0.1
CHOS	-0.1	-0.1	0.1	0.2	0.1	0.1	0.1	0.1	0.1	-0.1	0.1	-0.3	-0.5	-0.2
CHNOS	0.0	0.2	-0.1	0.0	-0.2	-0.3	-0.3	-0.2	-0.1	0.0	0.2	-0.2	#####	-0.1
NOSC	DI	5.5	9.9	10.5	11.5	12.5	13.5	14.5	15.5	16.5	17.5	18.5	19.5	20.5
CHO	0.0	-0.3	-0.5	-0.7	-0.7	-0.7	-0.5	-0.4	-0.3	-0.2	-0.2	-0.2	-0.1	-0.1
CHNO	-0.1	-0.1	-0.1	-0.3	-0.3	-0.2	-0.3	0.1	-0.2	-0.1	0.0	-0.4	-0.2	0.2
CHOS	-0.1	-0.2	-0.5	-0.5	-0.5	-0.3	-0.1	-0.1	-0.3	-0.2	-0.2	0.0	-0.2	0.3
CHNOS	-0.2	0.0	-0.1	-0.4	-0.4	-0.3	-0.4	0.0	0.2	-0.6	-0.2	-0.3	#####	0.9
DBE	DI	5.5	9.9	10.5	11.5	12.5	13.5	14.5	15.5	16.5	17.5	18.5	19.5	20.5
CHO	-0.1	0.6	-0.3	-0.6	-0.5	-0.5	-0.3	-0.4	-0.5	-0.4	-0.6	-0.2	-0.4	-0.3
CHNO	-0.4	0.4	-0.5	-0.7	-0.7	-0.6	-0.6	-0.6	-0.6	-0.5	-0.5	-0.3	-0.3	-0.3
CHOS	0.1	-0.1	0.0	-0.1	0.0	0.0	0.2	0.2	0.4	-0.1	0.1	-0.4	-0.5	0.1
CHNOS	0.1	0.2	-0.1	0.0	0.0	0.3	0.3	0.1	-0.5	0.1	-0.1	-0.6	#####	-0.7

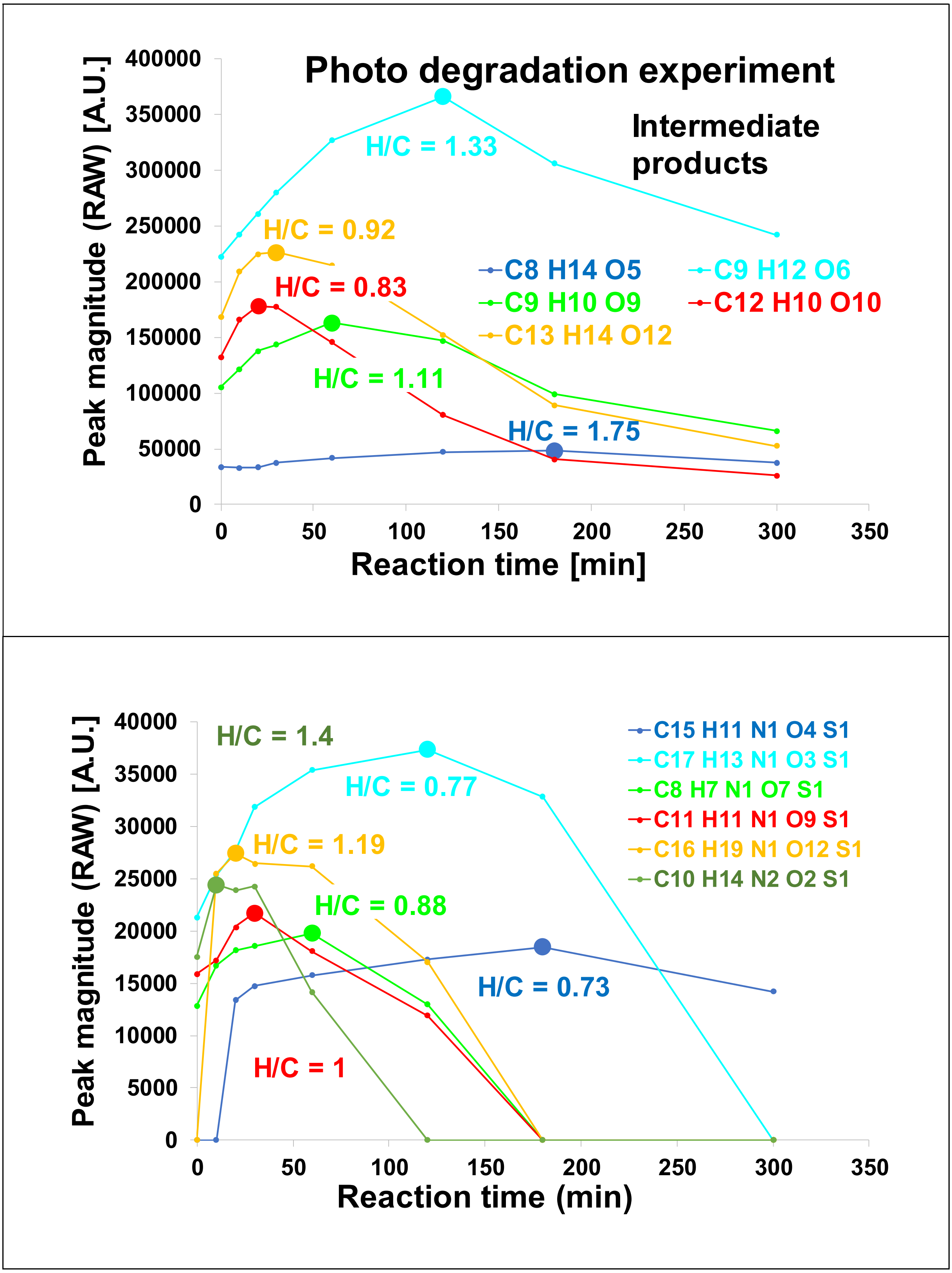

Fig. S26 ***IntP*** **maximum time point in relation to H/C, top examples for CHO components (RT = 10.5 Min), bottom examples for CHNOS (RT = 12.5 Min) components**

SI 9 Search for the opposite of *IntP*, an intermediate minimum

We postulate that

1. $(RAW(t_0) - \text{minimum } RAW(t_1 - t_6)) / \text{minimum } RAW(t_1 - t_6) > 0.265$

and
$(RAW(t_7) - \text{minimum } RAW(t_1 - t_6)) / \text{minimum } RAW(t_1 - t_6) > 0.265$
If presence count = 8
$\delta RAW_{max} = [\max RAW(t_1 - t_6) - RAW(t_1)] / RAW(t_1) > 0.265$
or

2. $S/N(p)(t_0)$ and $S/N(p)(t_7) > 12$; $S/N(p)(t_1)$ and $S/N(p)(t_6) > 4$;
   minimum $S/N(p)(t_2 - t_5) < S/N(4)$;

$S/N(t_0) > \text{maximum } S/N(p)(t_2 - t_5) < S/N(p)(t_7)$
If presence count < 8

Table S6 Number of MF showing an intermediate minimum

RT	5.51	9.86	10.5	11.5	12.5	13.5	14.5	15.5	16.5	17.5	18.5	19.5	20.5
1.	0	0	1	0	0	0	0	0	0	0	0	0	0
2.	0	0	1	1	1	1	0	1	2	0	0	1	0

RT	DI
1.	7
2.	15

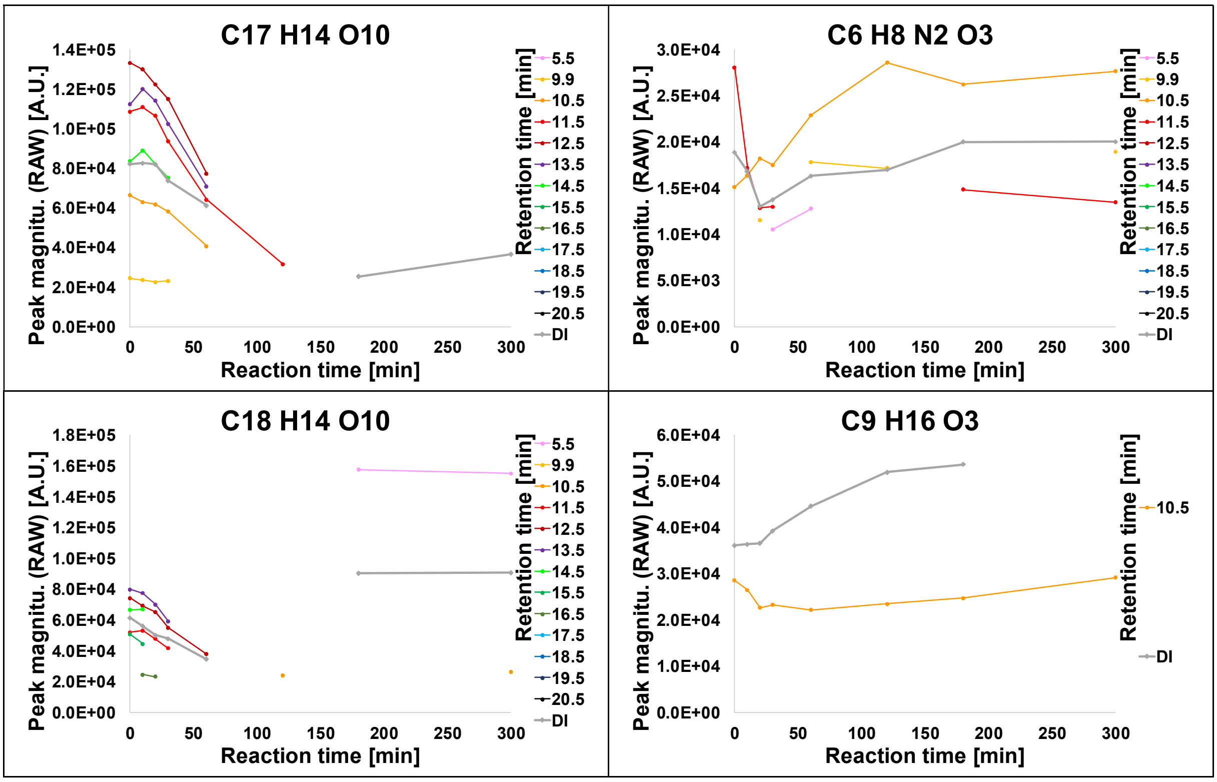

Fig. S27 MF showing intermediate minimum, DI top left, DI and RT = 11.5 min top right, DI bottom left, RT = 10.5 min bottom right

SI 10 Totaling opposite reactivity classes of the same MF

For each MF it is searched if at least one of the reactivity classes *Prod* (*Prod* + <*Prod*), *Degr* (*Degr* + *Degr*>), *IntP* (*IntP* + <*IntP*> + <*IntP* + *IntP*>), *Res* and n.r.a. was found at 12 different RTs (20.5 min was excluded from this search).

Table S7 Which reactivity classes were found for MF, a balance

found	*Prod*	*Degr*	*IntP*	*Res*	*r.n.a.*
one	84	582	446	274	1152
two				486	486
two			1353		1353
two		1480			1480
two	53				53
two			65	65	
two		94		94	
two	2			2	
two		414	414		
two	10	10			
three			296	296	296
three		368		368	368
three		1378	1378		1378
three	30			30	30
three	106		106		106
three	18	18			18
three	5		5	5	
three	8	8		8	
three	9	9	9		
four		380	380	380	380
four	57		57	57	57
four	32	32		32	32
four	40	40	40		40
four	4	4	4	4	
Five	34	34	34	34	34

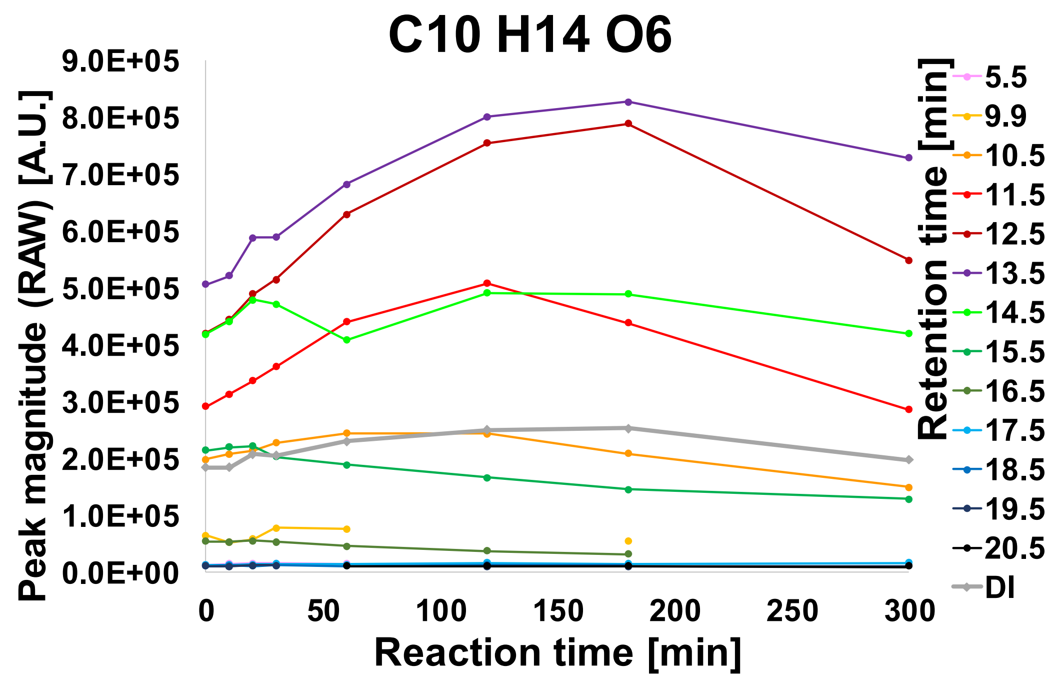

5.5	9.9	10.5	11.5	12.5	13.5	14.5	15.5	16.5	17.5	18.5	19.5	20.5
IntP>	r.n.a.	Res	IntP	IntP	Prod	Res	Degr	Degr	Res	Res	r.n.a.	Res
IntP>	r.n.a.	Res	IntP	IntP	Prod	Res	Degr	Degr	Res	Res	r.n.a.	Res
IntP>	r.n.a.	Res	IntP	IntP	Prod	Res	Degr	Degr	Res	Res	r.n.a.	Res
IntP>	r.n.a.	Res	IntP	IntP	Prod	Res	Degr	Degr	Res	Res	r.n.a.	Res
IntP>	r.n.a.	Res	IntP	IntP	Prod	Res	Degr	Degr	Res	Res	r.n.a.	Res
IntP>	r.n.a.	Res	IntP	IntP	Prod	Res	Degr	Degr	Res	Res	r.n.a.	Res
IntP>	r.n.a.	Res	IntP	IntP	Prod	Res	Degr	Degr	Res	Res	r.n.a.	Res
IntP>	r.n.a.	Res	IntP	IntP	Prod	Res	Degr	Degr	Res	Res	r.n.a.	Res

P	Q	R	S	T	U	V	W	X	Y	Z	AA	AB	AC
time 1	5.5	9.9	10.5	11.5	12.5	13.5	14.5	15.5	16.5	17.5	18.5	19.5	20.5
0	12926	64665	198641	292124	420961	506010	419039	214433	54697	13578	11669	11512	
10	15166	53269	207759	313673	443914	521120	441584	220686	54253	13058	12469	10677	
20	16590	58906	214910	337435	489045	588390	479151	221878	55401	14240	11359	12267	
30	16517	77972	228334	361564	514711	589498	471439	203398	53822	15272	12020	11938	
60	15364	76424	244467	440224	629584	682430	408523	188862	46094	14612	11798		11566
120			244351	507991	754469	801193	491190	166410	37715	17388	13158	10814	11390
180		55439	209177	438548	789088	827612	489675	146288	32363	14863	12111	10507	11354
300			150242	285979	548468	728374	420282	128729		16717		10979	11279

Fig. S28 **Example for an MF showing five different reactivity classes for different RTs**

SI 11 Degradation of potential surfactant metabolites

Linear alkyl benzene sulfonates and their coproducts dialkyl tetralin sulfonates and their metabolites sulfophenyl carboxylic acids were detected in secondary-treated wastewater (Gonsior et al.).
The degradation of some potential surfactant metabolites containing 5 oxygen atoms (sulfonic acid + carboxylic acid suggested) are presented.

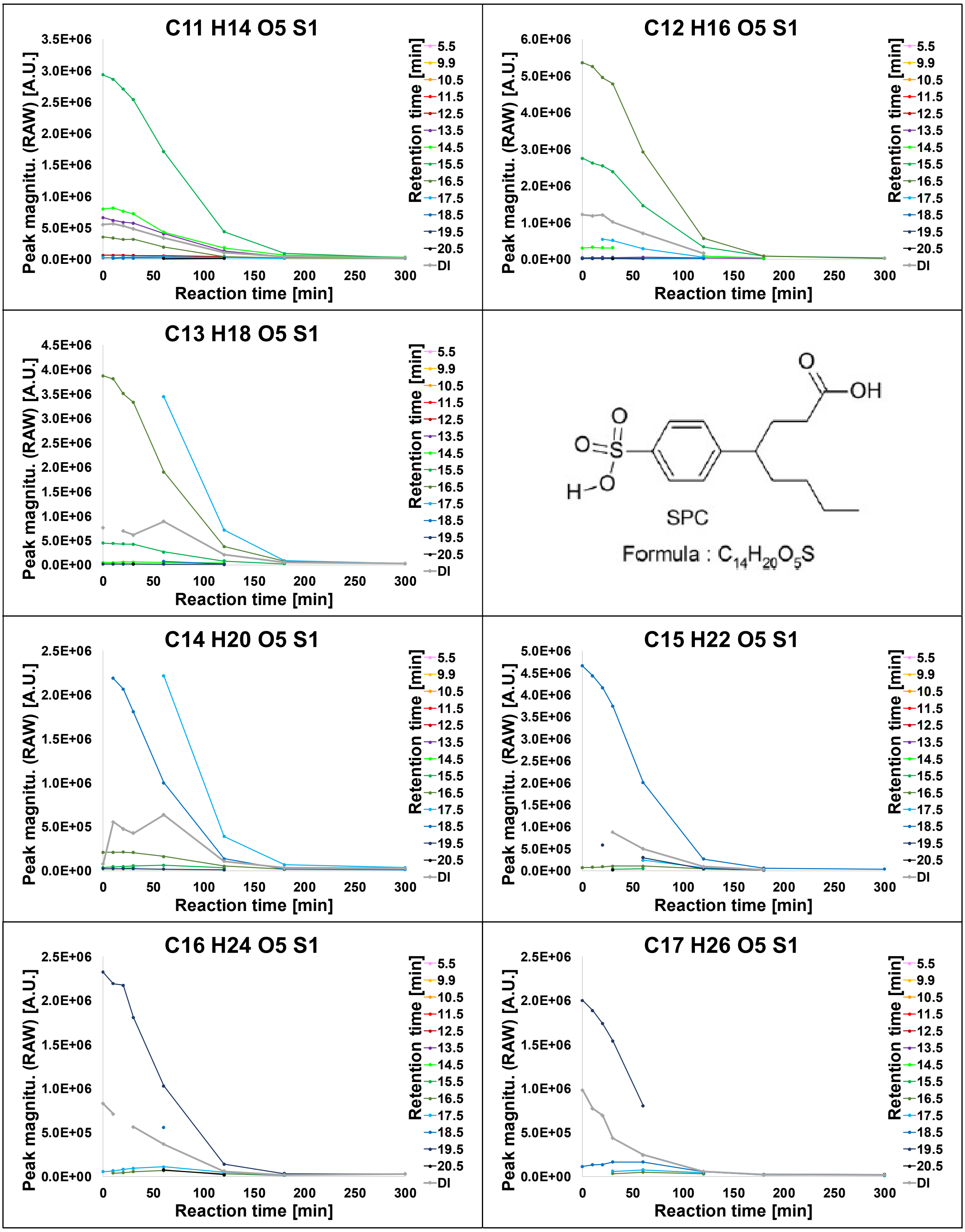

Fig. S29 **Reaction time courses for potential linear alkylsulfonate metabolites**

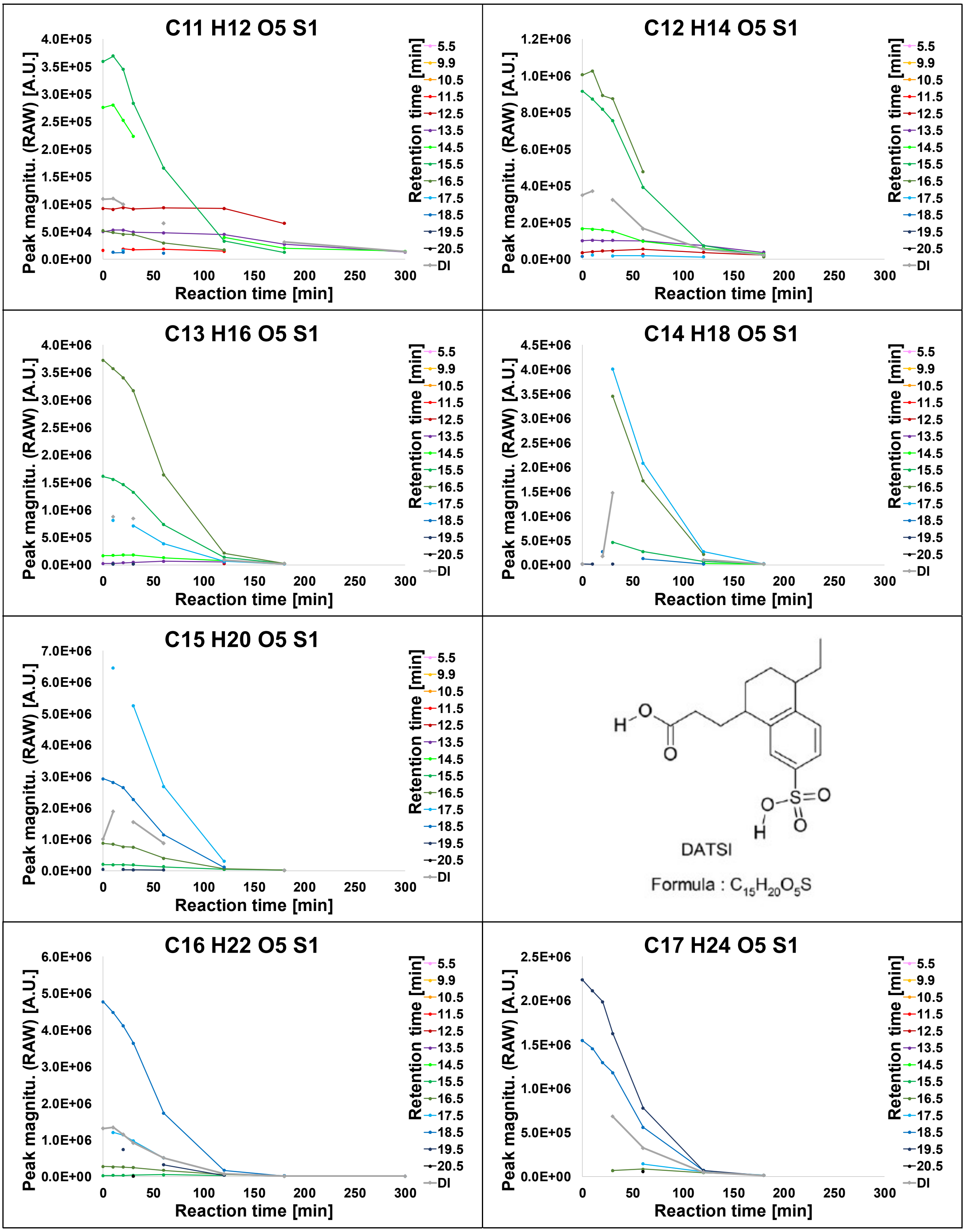

Fig. S30 **Reaction time courses for potential dialkyl tetralin sulfonates metabolites**

SI 12 Comparison to data sets from the literature

The next step of investigation in the future would be to apply the current model to different water types and / or different degradation conditions. This need not be necessarily photo (or thermal) degradation. In addition, microbial transformation experiments can be taken into account. In our data set just one waste water sample, executing two replicates of an irradiation experiment under only one selected processing condition is available. The focus was on the data evaluation strategy. However, we have access to one data set from the literature providing high time resolution. Water of a small shaded forested river, containing high amounts of humic substances, probably minor anthropogenically influenced, has been irradiated with natural sunlight for some days. The corresponding DI-FT-ICR-MS data set is available [2]. We selected eight time dependent data points (T0, T2, T3, T4, T8, T10, T11, T12) from this literature data set (providing together 13 time points, SI_data_base, sheet “median rel. intensity values”, 0.2 µm) in order to execute our data evaluation procedure. A direct comparison of found reactivity classes balances is possible with our calculated DI-FT-ICR-MS data set from the waste water sample. It is clear that limitations have to be considered due to the different process conditions.

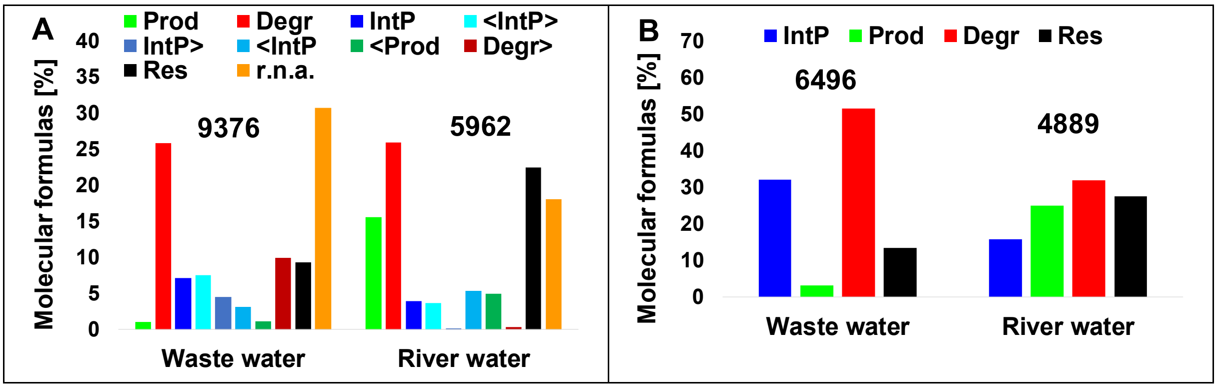

Fig. S31 Comparison of DI-FT-ICR-MS photo irradiation data from waste water and river water, A detailed and B simplified reactivity classes

The results indicate (Fig. S31) that significant differences will be found if water type and / or process conditions are changed. The waste water possibly is containing more easy photo degradable substances and in addition the process conditions are more drastic (catalysis using TiO_2 powder) compared to the river water which was irradiated only with natural sunlight. Due to this, the percentage part of degraded components was higher in the waste water and more *IntP* were found compared to the river water. The river water evidently contained more photostable MFs and more Prod were generated. Nevertheless *IntP*, which have not been addressed before in the DOM / FT-ICR-MS literature, do play a significant role also in river water. As a conclusion, *IntP* should be generally considered in future experiments.

References

[1] Kümmerer, K. Pharmaceuticals in the environment. Annual Review of Environment and Resources 35 (2010), 57-75.

[2] Wilske, C.; Herzsprung, P.; Lechtenfeld, O. J.; Kamjunke, N.; von Tümpling, W. Photochemically Induced Changes of Dissolved Organic Matter in a Humic-Rich and Forested Stream. Water 2020, 12 (2), 331.
